# Luvometinib in patients with Langerhans cell histiocytosis, Erdheim–Chester disease, and other histiocytic neoplasms: a single-arm, multicentre, phase 2 study

**DOI:** 10.1016/j.eclinm.2025.103486

**Published:** 2025-09-17

**Authors:** Xin-Xin Cao, Qi Zhu, Zhen Cai, Jie Ma, Hui Zhou, Long Chang, Lai-Ping Zhong, Zhu-Li Wu, Xingli Wang, Pu Han, Hong-Mei Lin, Zhen Wei, Jia-Yan Guo, Yang Zheng, Jian Li

**Affiliations:** a Department of Hematology, State Key Laboratory of Complex Severe and Rare Diseases, Peking Union Medical College Hospital, Chinese Academy of Medical Science & Peking Union Medical College, Beijing, China; b Department of Medical Oncology, National Cancer Center/National Clinical Research Center for Cancer/Cancer Hospital, Chinese Academy of Medical Sciences & Peking Union Medical College, Beijing, China; c Department of Oral & Maxillofacial-Head & Neck Oncology, Shanghai Ninth People's Hospital, Shanghai Jiaotong University School of Medicine, Shanghai, China; d Department of Bone Marrow Transplantation Center, The First Affiliated Hospital of Zhejiang University School of Medicine, Hangzhou, Zhejiang, China; e Department of Hematology, The First Affiliated Hospital of Zhengzhou University, Zhengzhou, China; f Department of Lymphoma & Hematology, Hunan Cancer Hospital, Changsha, Hunan, China; g Department of Oromaxillofacial Head and Neck Surgery, Center for Oromaxillofacial Head and Neck Disorders, Huashan Hospital, Fudan University, Shanghai, China; h Shanghai Fosun Pharmaceutical Industrial Development Co., Ltd., Shanghai, China; i Beijing Fosun Pharmaceutical Research and Development Co., Ltd., Beijing, China; j State Key Laboratory of Common Mechanism Research for Major Diseases, Chinese Academy of Medical Science & Peking Union Medical College, Beijing, China

**Keywords:** Histiocytic neoplasm, MEK1/2 inhibitor, Luvometinib, Langerhans cell histiocytosis, Erdheim–Chester disease

## Abstract

**Background:**

Histiocytic neoplasms are a heterogeneous group of haematologic disorders marked by a high frequency of mutations in the somatic mitogen-activated protein kinase pathway. This single-arm, multicentre, phase 2 study evaluated the efficacy and safety of the selective MEK1/2 inhibitor luvometinib in adult patients with histiocytic neoplasms.

**Methods:**

Patients (aged >16 years), regardless of tumour genotype and who were either treatment-naïve or relapse/refractory, were enrolled and received oral luvometinib, 8 mg, once daily in 28-day cycles until disease progression, death, unacceptable toxicity, withdrawal of consent, or end of the study. The primary end point was overall response rate (ORR) assessed by an independent review committee according to positron emission tomography response criteria. This trial was registered with chinadrugtrials.org.cn (CTR20221069) and chictr.org.cn (ChiCTR2300067955).

**Findings:**

Between June 27, 2022 and February 2, 2024, 30 patients were enrolled; they were followed up for a median duration of 16.2 months (range, 1.5–19.3). In 29 evaluable patients, 22 (75.9%) had Langerhans cell histiocytosis, 3 (10.3%) had Erdheim–Chester disease, and 4 (13.8%) had other subtypes. Most (86.2%) patients had previously received systemic therapy and 27.6% had received ≥3 lines. With a median follow-up of 16.2 months, the ORR was 82.8% (95% CI, 64.2–94.2), with a median time to response of 2.9 months (range, 2.6–6.0) and median duration of response not reached. The 12-month progression-free survival rate was 74.4% (95% CI, 49.8–88.2). Grade ≥3 treatment-emergent adverse events occurred in 13 (43.3%) patients, with folliculitis (10.0%), hypertriglyceridemia (10.0%), and blood creatine phosphokinase increased (6.7%) occurring in more than one patient. No treatment-emergent adverse events led to treatment discontinuation.

**Interpretation:**

Luvometinib demonstrated high and durable responses and a manageable safety profile in patients with histiocytic neoplasms.

**Funding:**

Shanghai Fosun Pharmaceutical Industrial Development Co., Ltd.

**Research in context**

**Evidence before this study**

We searched PubMed for articles published between database inception and April 07, 2025, with the terms “histiocytic neoplasms” OR “Langerhans cell histiocytosis” OR “Erdheim–Chester disease” OR “juvenile xanthogranuloma” OR “Rosai–Dorfman disease” AND “trial” OR “phase” OR “clinical study” in titles or abstracts with no language restrictions and manually screened for clinical trials of targeted therapy for these diseases. We identified 14 reports; among them, antitumour activity was shown for the BRAF V600 kinase inhibitor vemurafenib in adult patients with Erdheim–Chester disease (ECD) and Langerhans cell histiocytosis (LCH) harbouring *BRAF* V600 mutation, vemurafenib combined with cladribine and cytarabine in children with *BRAF* V600E-positive LCH with or without risk organs, the BRAF inhibitor dabrafenib as monotherapy and in combination with the MEK1/2 inhibitor trametinib in children with *BRAF* V600-mutant LCH, and the MEK1/2 inhibitor cobimetinib in adult patients with histiocytic neoplasms (LCH and ECD). Except for cobimetinib, robust evidence from clinical trials is limited to support the efficacy of other targeted agents in patients with histiocytic neoplasms across different genotypes.

**Added value of this study**

The present phase 2 study enrolled patients with histiocytic neoplasms of more diverse subtypes (with LCH being the predominant subtype) and genotypes, and the sample size (n = 30) was relatively large compared with previous studies. Luvometinib, a selective MEK1/2 inhibitor, demonstrated high and durable antitumour activity in patients with histiocytic neoplasms, regardless of subtype or genotype. Luvometinib showed a well-tolerated safety profile, with no treatment-emergent adverse events leading to treatment discontinuation.

**Implications of all the available evidence**

Targeted treatment options for patients with histiocytic neoplasms, regardless of biomarker status, are limited and not all patients globally have access to the approved agents because of regional approvals. The results from this study suggested that luvometinib could be an alternative option for patients with histiocytic neoplasms, regardless of disease subtypes and genotypes.

## Introduction

Histiocytic neoplasm is a rare, heterogeneous group of haematologic disorders characterised by the accumulation and infiltration of macrophages, dendritic cells, or monocyte-derived cells in various tissues and organs.^1^ Histiocytic neoplasms encompass disorders including Langerhans cell histiocytosis (LCH), Erdheim–Chester disease (ECD), juvenile xanthogranuloma (JXG), Rosai–Dorfman disease (RDD), histiocytic sarcoma, indeterminate cell histiocytosis, follicular dendritic cell sarcoma, and interdigitating dendritic cell sarcoma.^2^ LCH, the most common subtype, is found in 5–9 cases per million children and 1–2 cases per million adults.^3^, ^4^, ^5^

At the molecular level, genetic mutations in the mitogen-activated protein kinase (MAPK) pathway are one hallmark of histiocytic neoplasm.^2^^,^^6^ The MAPK pathway regulates cell growth, differentiation, and survival. The constitutive activation of the MAPK signalling pathway contributes to tumour cell proliferation and cell resistance to apoptosis.^7^ Approximately 50–55% of cases with LCH or ECD have *BRAF*^V600E^ mutations, and mutations of other genes in the MAPK pathway are found in 1–30% of cases.^8^

Targeting the nodes along the MAPK pathway for the treatment of histiocytic neoplasms has shown promising results in clinical trials. The *BRAF* V600 kinase inhibitor vemurafenib demonstrated clinical activity in patients with LCH or ECD harbouring *BRAF*^V600^ mutations in a phase 2 trial.^9^ Cobimetinib, a MEK1/2 inhibitor, elicited tumour response across a variety of histiocytic neoplasms, regardless of genotype, in a phase 2 study.^10^ In addition, a retrospective analysis showed antitumour activity of trametinib plus dabrafenib in patients with high-risk histiocytic neoplasms with the *BRAF*^V600^ mutation, and of trametinib monotherapy in those without the *BRAF*^V600^ mutation.^11^ Although vemurafenib and cobimetinib have been approved by the United States Food and Drug Administration for the treatment of patients with ECD harbouring the *BRAF*^V600^ mutation and those with histiocytic neoplasms regardless of the genotype, respectively, patients in the other regions do not have access to these targeted treatments.^12^^,^^13^

Luvometinib is a highly potent and selective MEK1/2 inhibitor that blocks the MAPK signalling and has proven to inhibit tumour growth in pre-clinical studies.^14^ Luvometinib exhibits binding affinity for ATP via a metastable binding site, thereby forming a triple complex with MEKase and ATP, which is analogous to the mechanism of action exhibited by trametinib. The crystal structure suggests that the binding pocket of trametinib is formed by the kinase suppressor of RAS (KSR)-MEK interaction interface.^15^ Furthermore, luvometinib can capture the “hot” spot of all key variant binding sites, suggesting its potential as a dual MEK1/2 inhibitor through efficient binding.

Luvometinib showed antitumour activity in patients with *NRAS*-mutant melanoma and those with neurofibromatosis type 1-related plexiform neurofibroma in clinical trials.^16^^,^^17^ This phase 2, multicentre, open-label, prospective trial was conducted to evaluate the efficacy and safety of luvometinib in patients with histiocytic neoplasms of various subtypes and genotypes.

## Methods

### Study design and patients

This single-arm, open-label, multicentre phase 2 study was conducted at seven sites in China. Eligible patients were >16 years old and had histologically confirmed treatment-naive or recurrent/refractory LCH, ECD, and other histiocytic neoplasms (diagnosed according to the 5th edition of the World Health Organization Classification of Haematolymphoid Tumours),^18^ evaluable lesions based on positron emission tomography (PET) response criteria (PRC), and an Eastern Cooperative Oncology Group (ECOG) performance status score of 0–2. For LCH, patients who had multisystem disease or single-system disease with more than one lesion were included. Patients who had previously received MEK1/2 inhibitors were excluded. Details of the inclusion and exclusion criteria are provided in the Supplementary Material. Patients or members of the public were not involved in the design, conduct, and reporting of the trial.

### Ethics

The trial was conducted in compliance with the principles of the Declaration of Helsinki, Good Clinical Practice guidelines developed by the International Council for Harmonisation, and local applicable regulatory requirements for clinical trials. The study protocol, amendments, and informed consent were reviewed and approved by the institutional review board committee at each study centre (ethical approval numbers are available in Supplementary Table S1). All patients provided written informed consent before their participation.

### Randomisation and masking

This was a single-arm, open-label study. Randomisation and masking were not performed.

### Procedures

Patients received oral luvometinib, 8 mg, once daily in 28-day cycles until disease progression, death, unacceptable toxicity, withdrawal of consent, or end of the study.

Tumour assessments were done using 18-F-fluoro-2-deoxyglucose positron emission tomography (^18^FDG-PET)/computed tomography (CT) and magnetic resonance imaging (MRI)/CT. Tumour response was evaluated by the independent review committee (IRC) and investigators according to PRC as the primary evaluation and per RECIST 1.1 as a complementary evaluation. Tumour assessments per PRC were performed at baseline and end of cycles 3, 6, and 12, and end of treatment. Assessments per RECIST 1.1 were conducted at baseline, end of cycles 3, 6, 9, 12, and end of treatment After cycle 12, assessments were conducted every four cycles per RECIST 1.1 if tumours were considered to be stable based on the imaging results; if tumours were not stable based on the imaging results, assessments were carried out every three to six cycles per PRC and every three cycles per RECIST 1.1. Complete response (CR) and partial response (PR) were confirmed at least 4 weeks after the first documentation.

Safety was monitored throughout the study and for 30 days after the last dose of luvometinib. Adverse events (AEs) were coded according to Medical Dictionary for Regulatory Activities Terminology (MedDRA) version 26.0 and graded per National Cancer Institute Common Terminology Criteria for Adverse Events (CTCAE) version 5.0. For patients with histiocyte tissue biopsies available for central laboratory testing, genomic analyses for MAPK pathway mutations were performed with next-generation sequencing (NGS) using the OncoScreen Plus panel at screening. Cell-free DNA (cfDNA) in blood was collected at baseline, at each time point corresponding to the RECIST version 1.1 tumour assessment, and after progressive disease (PD) for central laboratory test, using the OncoCompass Plus NGS panel (∼20000X mean sequencing depth, Burning Rock, Co. Ltd., Guangzhou, China).

### Outcomes

The primary end point was overall response rate (ORR) assessed by the IRC according to PRC, defined as the proportion of patients who had complete metabolic response (CMR; normalisation of all lesions to at or below the liver standardized uptake value [SUV_liver_] background) and partial metabolic response (PMR; ≥50% decrease from baseline in the sum of SUV of all target lesions). Secondary end points included disease control rate (DCR), clinical benefit rate (CBR), time to response (TTR), and progression-free survival (PFS), assessed by the IRC and investigators per PRC and RECIST 1.1. Additional secondary end points were investigator-assessed ORR per PRC, and IRC- and investigator-assessed ORR per RECIST 1.1, overall survival (OS), and safety. Treatment-emergent adverse event (TEAE) was defined as any adverse event occurring from the first dose of the study treatment to 30 days after the last dose of the study treatment and any treatment-related adverse event (TRAE) or serious adverse event occurring after 30 days of the last dose of the study treatment. TEAEs that were considered by the investigators to be definitely related, probably related, or possibly related to the study treatment were classified as TRAEs. The changes in variant allele frequency (VAF) of MAPK pathway mutations in cfDNA and their relationship with efficacy were assessed as a prespecified exploratory end point. PRC criteria and definitions of efficacy end points are provided in the Supplementary Material.

### Statistical analysis

Assuming an ORR of 0.80, approximately 25 evaluable patients were required to generate a two-sided 95% confidence interval (CI) with a width of 33.9%. A total of 28 patients were planned to be enrolled assuming a dropout rate of 10%. The ORRs and the corresponding 95% CIs for different numbers of responders are shown in the Supplementary Material.

Efficacy was analysed in patients who provided informed consent, had taken at least one dose of luvometinib, had a baseline tumour assessment and at least one postbaseline tumour assessment, and had no major protocol violations. Safety analysis set included all patients who had taken at least one dose of luvometinib and had at least one safety assessment.

The 95% CI for ORR was calculated using the Clopper–Pearson method. PFS and OS were estimated using the Kaplan–Meier method. Prespecified subgroup analysis of ORR was conducted based on baseline characteristics. All analyses were performed using SAS (versions 9.4; SAS Institute Inc). The date of data cutoff for the prespecified analysis was February 02, 2024. The study was registered with chinadrugtrials.org.cn (identifier: CTR20221069) and chictr.org.cn (identifier: ChiCTR2300067955).

### Role of the funding source

Shanghai Fosun Pharmaceutical Industrial Development Co., Ltd. funded the study. The sponsor collected, analysed, and interpreted data in conjunction with the authors. All authors had access to the data and participated in the reviewing of the manuscript.

## Results

**Fig. 1:**
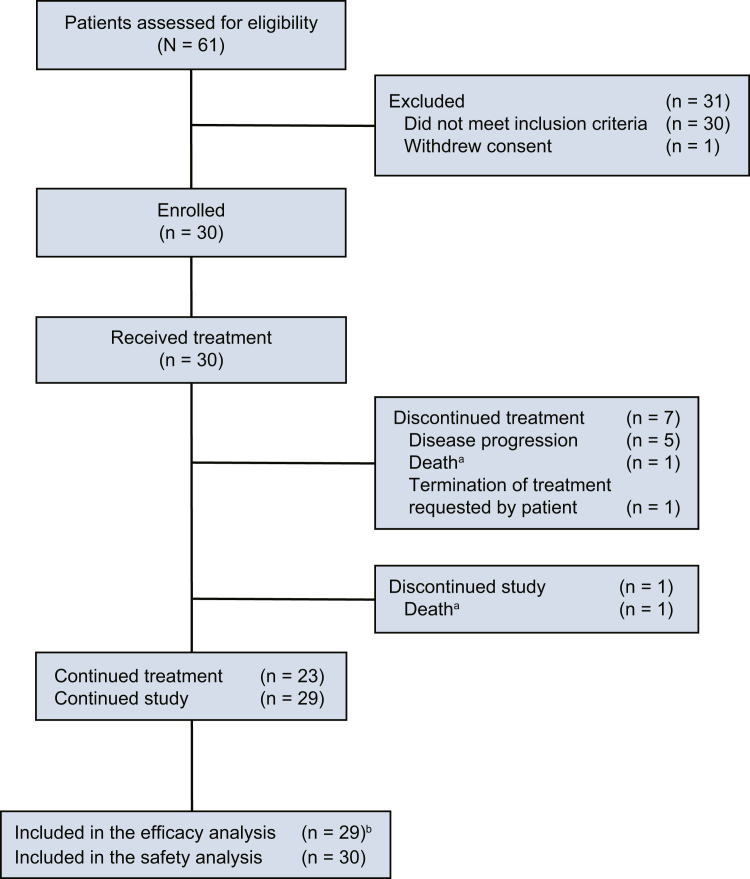
**Patient disposition.**^a^Occurred in the 1 patient with major protocol violation (misdiagnosis). ^b^1 patient was excluded from analysis due to major protocol violation (misdiagnosis) and no post-baseline tumour assessment.

From June 27, 2022 to February 2, 2024, a total of 30 patients were enrolled and received treatment (Fig. 1). The median duration of follow up was 16.2 months (range, 1.5–19.3). The median duration of treatment was 13.8 months (range, 0.9–19.4). One patient had incorrect disease diagnosis, which was considered as a major protocol violation. This patient was excluded from efficacy analysis; efficacy analysis included 29 patients and the safety set included 30 patients.

Among 29 patients analysed for efficacy, the diagnosed histiocytic neoplasm subtypes included LCH (n = 22 [75.9%]), ECD (n = 3 [10.3%]), mixed LCH/ECD (n = 1 [3.4%]), mixed LCH/JXG (n = 1 [3.4%]), and RDD (n = 2 [6.9%]) (Table 1). Most (86.2%) patients had previously received systemic anticancer treatment; six (20.7%) had received two lines and eight (27.6%) had received three or more lines. The most common prior therapy was chemotherapy (88.0%, 22/25), followed by immunosuppressive therapy (60.0%, 15/25), interferon-α (8.0%, 2/25), and BRAF inhibitors (8.0%, 2/25).

**Table 1:** Baseline demographics and disease characteristics.

	Patients included in efficacy analysis (n = 29)
Median age (IQR), years	36.0 (24.0–47.0)
Sex, n (%)^a^	
Male	16 (55.2)
Female	13 (44.8)
Race, n (%)^a^	
Asian	29 (100)
ECOG PS, n (%)	
0	22 (75.9)
1	6 (20.7)
2	1 (3.4)
Subtypes of histiocytic neoplasms, n (%)	
LCH	22 (75.9)
ECD	3 (10.3)
LCH plus ECD	1 (3.4)
LCH plus JXG	1 (3.4)
RDD	2 (6.9)
System involvement	
Multifocal single-system	3 (10.3)
Multisystem	26 (89.7)
Central nervous system involvement	
Yes	18 (62.1)
Pituitary	15 (51.7)
Brain parenchyma	4 (13.8)
Brain meninges	3 (10.3)
Other	1 (3.4)
No	11 (37.9)
MAPK pathway mutation,^b^ n (%)	
***BRAF***	18 (62.1)
V600E	5 (17.2)
*BRAF* InDel	10 (34.5)
N486_P490del	8 (27.6)
N486_T491delinsK	2 (6.9)
*BRAF* fusion	3 (10.3)
*ARRB1-BRAF*	1 (3.4)
*BICD2-BRAF*	1 (3.4)
*PICALM-BRAF*	1 (3.4)
***ARAF***	1 (3.4)
Q352_F354delinsL	1 (3.4)
***MAP2K1***	7 (24.1)
E102_I103del/L101_I103delinsM	5 (17.2)
C121S	1 (3.4)
F53_Q58delinsL	1 (3.4)
Prior treatment lines, n (%)	
0	4 (13.8)
1	11 (37.9)
2	6 (20.7)
≥3	8 (27.6)
Prior treatment types,^c^ n (%)	
Chemotherapy^d^	22 (88.0)
Immunosuppressive therapy	15 (60.0)
Interferon-α	2 (8.0)
*BRAF* inhibitors	2 (8.0)
Others	6 (24.0)

ECOG PS, Eastern Cooperative Oncology Group performance status; ECD, Erdheim–Chester disease; JXG, juvenile xanthogranuloma; LCH, Langerhans cell histiocytosis; RDD, Rosai–Dorfman disease.

a Sex and race were self-reported by patients.

b No mutations in tissue or peripheral blood were identified in 2 patients.

c Percentages were calculated using the total number of people who received at least one prior treatment as the denominator.

d Prior first-line chemotherapy regimens included cytarabine, VP (vindesine plus prednisone), MA (methotrexate and cytarabine), CEVP (cyclophosphamide, etoposide, vindesine, and prednisone), cladribine plus cytarabine, DAL-HX90 intensified regimen (vinorelbine, vincristine, etoposide, and prednisone), TCD (thalidomide, cyclophosphamide, and dexamethasone acetate), vinorelbine plus azathioprine plus prednisone, CHOP (cyclophosphamide, doxorubicin, vindesine, and prednisone), CHOEP (cyclophosphamide, epirubicin, vincristine, etoposide, and prednisone), vinorelbine plus prednisone plus dexamethasone, COP (ifosfamide, vincristine, and prednisone), and methotrexate plus vincristine.

Twenty-six (89.7%) patients had a variety of MAPK pathway mutations involving *BRAF* (n = 5 [17.2%] with *BRAF*^V600E^, n = 10 [34.5%] with *BRAF*^Indel^, and n = 3 [10.3%] with *BRAF* fusion), *ARAF* (n = 1 [3.4%]), and *MAP2K1* (n = 5 [17.2%] with *MAP2K1*^E102_I103del^ or *MAP2K1*^L101_I103delinsM^ and n = 2 [6.9%] with *MAP2K1*^C121S^ or *MAP2K1*^F53_Q58delinsL^), and one patient had *CXCR4* copy number variation (n = 1 [3.4%]). In addition, no mutation was identified in the tissues or blood of two patients.

**Fig. 2:**
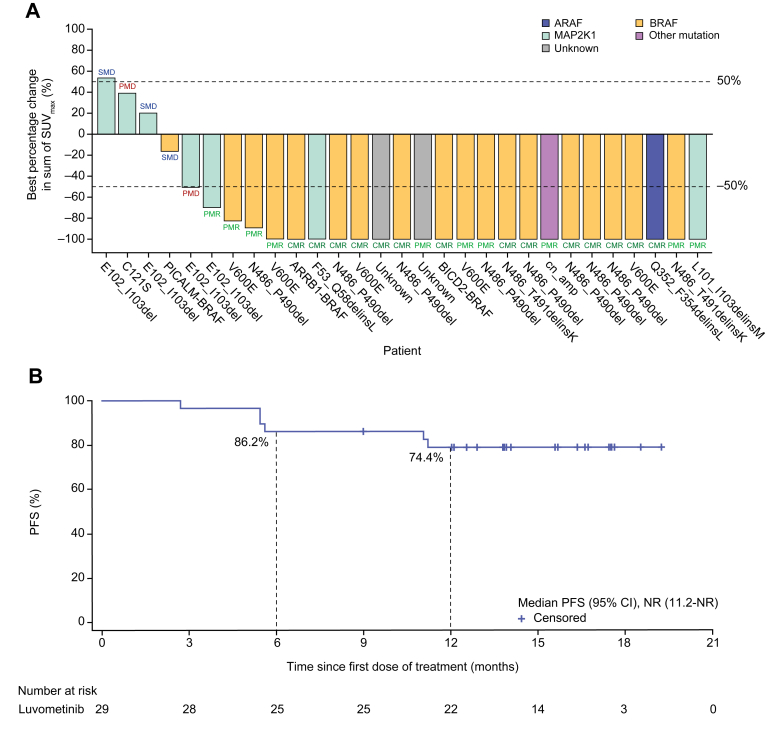
**Tumour response and progression-free survival.** (A) Waterfall plot of the maximum change from baseline in tumour metabolism according to SUVs assessed by IRC per PRC. (B) Kaplan–Meier plot of progression-free survival assessed by IRC per PRC. CMR, complete metabolic response; IRC, independent review committee; PFS, progression-free survival; PMD, progressive metabolic disease; PMR, partial metabolic response; PRC, positron emission tomography response criteria; SMD, stable metabolic disease; SUV, standardised uptake value.

The IRC-assessed ORR per PRC was 82.8% (95% CI, 64.2–94.2); 14 (48.3%) patients had CMR and 10 (34.5%) had PMR (Table 2 and Fig. 2A). The tumour response was observed across multiple subgroups defined based on tumour subtypes and genotypes in the prespecified subgroup analysis (Table 3). The ORR was 81.8% (95% CI, 59.7–94.8%) in 22 patients with LCH and 100% (95% CI, 29.2–100) in three patients with ECD. Subtypes of the mutant forms of the *BRAF*, *ARAF*, and *MAP2K1* genes demonstrated responsiveness to luvometinib according to IRC assessments per PRC. The ORR for patients carrying *BRAF* mutations (including the *BRAF*^V600E^, *BRAF*^InDel^, and *BRAF* fusion) was 94.4% (17/18), while the response rate for patients with a *MAP2K1* mutation was 42.9% (3/7), with the remaining patients demonstrating a 100% (4/4) response rate. Of five patients with *MAP2K1*^E102_I103del^ or *MAP2K1*^L101_I103delinsM^, the ORR was 40%. Among the two patients who had previously received *BRAF* inhibitors, the ORR was 100%. Subgroup analysis by prior line of treatment showed that the ORR was 100% (4/4) among patients who were treatment-naïve, 82.4% (14/17) for those who had received one to two prior lines, and 75.0% (6/8) for those who received three or more prior lines.

**Table 2:** Efficacy end points according to IRC assessments per PRC and RECIST 1.1.

	PRC	RECIST 1.1
Evaluable patients, n	29	16
Confirmed best overall response, n (%)		
CMR/CR	14 (48.3)	2 (12.5)
PMR/PR	10 (34.5)	7 (43.8)
SMD/SD	3 (10.3)	6 (37.5)
SMD/SD ≥24 weeks	1 (3.4)	5 (31.3)
PMD/PD	2 (6.9)	1 (6.3)
NE	0	0
ORR, % (95% CI)	82.8 (64.2–94.2)	56.3 (29.9–80.2)
TTR, months	2.9	3.0
DCR, % (95% CI)	93.1 (77.2–99.2)	93.8 (69.8–99.8)
CBR, % (95% CI)	86.2 (68.3–96.1)	87.5 (61.7–98.4)

CBR, clinical benefit rate; CMR, complete metabolic response; CR, complete response; DCR, disease control rate; IRC, independent review committee; NE, not evaluable; ORR, overall response rate; PD, progressive disease; PMD, progressive metabolic disease; PMR, partial metabolic response; PR, partial response; PRC, positron emission tomography response criteria; SD, stable disease; SMD, stable metabolic disease; TTR, time to response.

**Table 3:** Subgroup analysis of ORR assessed by IRC per PRC.

Subgroups	ORR patient numbers, n/N	ORR (95% CI)
Subtypes of histiocytic neoplasms		
LCH	18/22	81.8 (59.7–94.8)
ECD	3/3	100.0 (29.2–100.0)
LCH plus ECD	1/1	100.0 (2.5–100.0)
LCH plus JXG	1/1	100.0 (2.5–100.0)
RDD	1/2	50.0 (1.3–98.7)
***BRAF* mutations**	17/18	94.4 (72.7–99.9)
V600E mutation	5/5	100.0 (47.8–100.0)
*BRAF* InDel	10/10	100.0 (69.1–100)
*BRAF* fusion	2/3	66.7 (9.4–99.1)
***MAP2K1* mutations**	3/7	42.9 (9.9–81.6)
E102_I103del or L101_I103delinsM	2/5	40.0 (0.5–71.6)
Other *MAP2K1* mutations^a^	1/2	50.0 (1.3–98.7)
**Other mutations/unknown**	4/4	100.0 (39.8–100.0)

ECD, Erdheim–Chester disease; IRC, independent review committee; JXG, juvenile xanthogranuloma; LCH, Langerhans cell histiocytosis; ORR, overall response rate; PRC, positron emission tomography response criteria; RDD, Rosai–Dorfman disease.

a Other *MAP2K1* mutations include C121S and F53_Q58delinsL.

The median TTR was 2.9 months (range, 2.6–6.0) (Table 2). Median duration of response (DOR) was not reached (NR) (95% CI, NR–NR), with 2 of 24 patients with disease progressed; the 6-month DOR rate was 100% (95% CI, 100–100). DCR was 93.1% (95% CI, 77.2–99.2).

As of the data cutoff, six (20.7%) patients had progressive metabolic disease; the median PFS was NR (95% CI, 11.2–NR) (Fig. 2B). The 6-month PFS rate was 86.2% (95% CI, 67.3–94.6) and the 12-month PFS rate was 74.4% (95% CI, 49.8–88.2).

ORR per RECIST 1.1 was 56.3% (95% CI, 29.9–80.2) and DCR was 93.8% (95% CI, 69.8–99.8) (Table 2). The median PFS was also NR (95% CI, NR–NR); the 6-month and 12-month PFS rates were both 93.1% (95% CI, 75.1–98.2). Investigator-assessed ORRs are presented in Supplementary Table S2. The OS rate was 100.0% (95% CI, 100.0–100.0) at both 6 months and 12 months.

All 30 (100.0%) patients in the safety analysis set experienced at least one TEAE (Table 4). The most common TEAEs (≥30%) were upper respiratory tract infection (15/30, 50%), alanine aminotransferase increased (12/30, 40.0%), folliculitis (11/30, 36.7%), rash (11/30, 36.7%), COVID-19 (10/30, 33.3%), peripheral oedema (10/30, 33.3%), and diarrhoea (10/30, 33.3%). A total of 13 (43.3%) patients reported grade ≥3 TEAEs, with 3 folliculitis (10.0%), 3 hypertriglyceridemia (10.0%), and 2 blood creatine phosphokinase increased (6.7%) occurring in more than one patient. TRAEs at any grade were reported in 29 (96.7%) patients, and grade ≥3 TRAEs were observed in 10 (33.3%) patients, which are presented in Table 4. TEAEs and TRAEs in 29 patients excluding the patient with incorrect diagnosis are presented in Supplementary Table S3.

**Table 4:** Common TEAEs and TRAEs in patients treated with luvometinib.

n (%)	Patients included in safety analysis (n = 30)			
	TEAEs		TRAEs	
	Any grade	Grade ≥3	Any grade	Grade ≥3
Upper respiratory tract infection	15 (50.0)	0	1 (3.3)	0
Alanine aminotransferase increased	12 (40.0)	0	11 (36.7)	0
Folliculitis	11 (36.7)	3 (10.0)	11 (36.7)	3 (10.0)
Rash	11 (36.7)	1 (3.3)	10 (33.3)	1 (3.3)
COVID-19	10 (33.3)	0	0	0
Peripheral oedema	10 (33.3)	0	10 (33.3)	0
Diarrhoea	10 (33.3)	0	10 (33.3)	0
Eczema	8 (26.7)	1 (3.3)	7 (23.3)	1 (3.3)
Creatine phosphokinase increased	8 (26.7)	2 (6.7)	7 (23.3)	1 (3.3)
Paronychia	7 (23.3)	0	7 (23.3)	0
C-reactive protein increased	6 (20.0)	0	5 (16.7)	0
Constipation	6 (20.0)	0	4 (13.3)	0
Aspartate aminotransferase increased	6 (20.0)	0	6 (20.0)	0
Thrombocytopaenia	5 (16.7)	0	4 (13.3)	0
Hyperuricemia	5 (16.7)	0	3 (10.0)	0
Hypertriglyceridemia	4 (13.3)	3 (10.0)	4 (13.3)	3 (10.0)

Most common any-grade TEAEs in 15% or more of patients by systemic organ class preferred term and grade ≥3 TEAEs in more than one patient (>3.3%) are presented.

TEAEs, treatment-emergent adverse events; TRAEs, treatment-related adverse events.

Serious TEAEs were observed in four of 30 (13.3%) patients, which were peripheral neuropathy, cerebral infarction, respiratory failure, infectious pneumonia, and upper gastrointestinal bleeding (n = 1; 3.3% each); none were judged to be related to treatment. TEAEs leading to treatment interruption (most common, COVID-19 in 10%) and dose reduction (most common, folliculitis in 10%) occurred in 43.3% (13/30) and 16.7% (5/30) of patients, respectively; eight (26.7%) and five (16.7%) cases were deemed related to treatment, respectively. No TEAEs led to treatment discontinuation. One death (respiratory failure) was reported and was not related to study treatment; this was reported in the one patient with incorrect disease diagnosis. The cause of death was obstructive airway changes arising from aggravation of lung lesions.

Peripheral blood cfDNA samples were collected from 29 patients at baseline, different time points during treatment, and PD. A total of 13 (44.8%) patients were identified to have mutations in MAPK pathway genes (including *BRAF*^V600E^, *BRAF*^InDel^, *BRAF* fusion, and *MAP2K1*^mut^) in peripheral blood cfDNA at baseline (Supplementary Figure S1). The variant allele frequency (VAF%) of MAPK pathway gene mutations in cfDNA was high at baseline; a significant decrease (69.2%, 9/13) or undetected mutation (23.1%, 3/13) in cfDNA was observed after the second efficacy evaluation visit (six cycles of treatment) following luvometinib treatment, which was consistent with the confirmed best clinical response (ORR, 92.3%). The 13 patients were on treatment without disease progression. In one patient, the VAF% of *BRAF* ^*N486_P490del*^ in cfDNA dropped from 6% at baseline to undetectable following three cycles of treatment with luvometinib, in parallel with disappearance of tumour lesions after four cycles of treatment (Supplementary Figure S2).

## Discussion

In this study, luvometinib showed efficacy and a manageable safety profile in adult patients with histiocytic neoplasms. Notably, the majority of enrolled patients had LCH, addressing a critical evidence gap for a subtype that has been underrepresented in previous studies. Luvometinib demonstrated antitumour activity with an IRC-assessed ORR per PRC of 82.8%. Assessments by IRC, unlike those according to the investigators in previous studies of MEK or BRAF inhibitors in patients with histiocytic neoplasms, added to the validity of the findings in this study.^9^^,^^10^ Notably, clinical activity was observed across different subtypes and genotypes. Tumour response was sustained in most patients (22/24). The IRC-assessed median PFS per PRC was NR, with 6-month and 12-month rates of 86.2% and 74.4%, respectively. No patients in the efficacy analysis set had died.

Our study enrolled a heavily pre-treated patient population with histiocytic neoplasms, with LCH as the predominant subtype. A phase 2 trial of the MEK inhibitor cobimetinib included 18 patients, with only two (11%) patients having LCH and most having ECD (12 [67%]); 11% of patients had received three or more lines of prior treatment, and 50% had received chemotherapy.^10^ The present study enrolled patients with more diverse histological and molecular subtypes, with LCH being the predominant subtype. Furthermore, 27.6% of patients analysed for efficacy had received three or more lines of prior treatment, and 75.9% had previously received chemotherapy, indicating a difficult-to-treat patient population. A small proportion of patients who were treatment-naïve were also enrolled because no standard first-line target therapy is available for the treatment of histiocytic neoplasms in China. Luvometinib demonstrated antitumour activity across subgroups of treatment-naïve patients, those who had received one to two lines of systemic therapy, and those who had received three or more lines of systemic therapy, suggesting its potential to be used in various treatment settings. The IRC-assessed ORR per PRC in the present study was comparable with that of 89% shown in the trial of cobimetinib. The mammalian target of rapamycin (mTOR) inhibitor (sirolimus or everolimus) was also previously investigated in 20 patients with ECD that had *BRAF*^V600E^, *BRAF* wild type, or unknown *BRAF* genotypes and objective response (including either metabolic or radiologic response) was seen in 65% of patients.^19^

The clinical activity of luvometinib was observed across a variety of genotypes. The results indicated that patients with *BRAF* mutations exhibited a favourable response to luvometinib, with an ORR per PRC of 94.4% across all groups, including those with *BRAF*^V600E^, *BRAF*^indel^, or *BRAF* fusions. The antitumour activity of luvometinib in patients with *BRAF* mutations was comparable to that of the BRAF inhibitor vemurafenib, which showed an ORR per PRC of 100% in patients with LCH or ECD harbouring *BRAF*^V600^ mutations.^9^ Similarly, an observational study reported an ORR per PRC of 88% for a BRAF inhibitor (vemurafenib or dabrafenib) among patients with ECD who were predominantly *BRAF*^V600E^ positive.^20^ It is notable that both of the two patients who had progressed on BRAF inhibitors responded to luvometinib, indicating that luvometinib may be able to overcome tumour resistance to BRAF blockade. In addition, patients with mutations other than *BRAF* or *MAP2K1* also showed a good response to luvometinib (ORR, 100%). Another observation in our study was that out of the five patients carrying *MAP2K1*^E102_I103del^ or *MAP2K1*^L101_I103delinsM^ mutations, only two responded to luvometinib, with an ORR of 40%, lower than the overall ORR. Theoretically, the *MAP2K1*^E102_I103del^ mutation, as a RAF-independent mutation (class III variants), is naturally resistant to allosteric MEK inhibitors.^21^ Trametinib and luvometinib have similar MEK1/2 kinase domain-binding models and a previous in vitro study has shown that the *MAP2K1*^E102_I103del^ and *MAP2K1*^L101_I103delinsM^ mutations (class III variants) result in conformational changes in MEK1 β3-αC, and KSR remodels the metastable binding pocket of MEK1/2, leaving it in a constantly activated state.^15^^,^^22^^,^^23^ These changes may contribute to the decreased sensitivity to trametinib or luvometinib. A preclinical study of trametinib has shown that these mutations are insensitive to MEK1 inhibition.^24^ The lower response exhibited by patients with this subtype of mutation is consistent with previous studies, although further studies are needed to confirm the role of *MAP2K1* class III mutations as a driver of resistance to MEK inhibitors in patients with histiocytic neoplasms.

Luvometinib demonstrated a favourable safety profile, characterised by relatively low incidence rates of skin and gastrointestinal toxicities and a notable absence of ocular adverse events—a toxicity typically associated with MEK inhibitors. No TEAEs led to treatment discontinuation during the trial period. The safety profile of luvometinib was generally consistent with the adverse event categories reported for MEK inhibitors.^10^^,^^13^^,^^25^^,^^26^ Skin toxicity is common with MEK inhibitors, which may be attributed to the disruption of epidermal homoeostasis and inflammatory skin response under inhibition of MEK signalling.^27^ Skin toxicity is also common with BRAF inhibitors, which led to treatment discontinuation among patients who received vemurafenib in a study.^20^ Most cutaneous events with luvometinib were mild to moderate and manageable with dose modifications and treatment. Of note, skin rash, which is the most common skin toxicity for MEK inhibitors, was reported in 36.7% of patients who received luvometinib, which is much lower than that reported with cobimetinib (83%).^10^ Gastrointestinal toxicities were reported in patients receiving luvometinib, including diarrhoea in 33.3% of patients, constipation in 20.0%, and other gastrointestinal events occuring in less than 15%; however, the rates were lower than those observed with cobimetinib.^10^ Gastrointestinal toxicities reported for cobimetinib included diarrhoea (72%), nausea (39%), vomiting (28%), constipation (22%), and gastrointestinal symptoms (22%). Ocular toxicity is also seen with MEK inhibitors, with retinal vein occlusion observed in 6% of patients treated with cobimetinib, resulting in permanent treatment discontinuation,^10^ while there was no relevant event occurrence that warranted attention with luvometinib in this study. No unexpected events were detected in this study compared to other approved MEK inhibitors.^13^^,^^25^^,^^26^ Furthermore, patients with adult-onset histiocytosis harbouring *BRAF*^del^ frequently present with sclerosing cholangitis,^28^ but none of the patients had a history of sclerosing cholangitis nor developed it as an adverse event in the current study. Overall, luvometinib showed an acceptable safety profile in patients with histiocytic neoplasms and a favourable tolerability among MEK inhibitors.

A series of mutations in genes in the MAPK pathway have previously been identified in both LCH and ECD, including mutations in *BRAF*, *MAP2K1*, and *ARAF*.^8^ In this study, a broad spectrum of genotypes were identified, and the results provided additional insights into the antitumour activity of luvometinib across different genotypes and the change in gene mutation frequency under treatment. The frequency of relevant MAPK pathway-related gene mutations in peripheral blood cfDNA was significantly decreased after treatment with luvometinib. The decreased levels were consistently maintained at the lower limit of detection or were not detected on subsequent treatment of luvometinib. All the patients achieved significant clinical benefit, which was assessed as CMR, PMR, or stable metabolic response.

Furthermore, our data indicated that cfDNA levels at baseline and after six cycles of treatment were associated with clinical outcomes. Monitoring of cfDNA levels of *MAPK* pathway-related mutated genes during treatment may provide valuable insights into the response to therapy.

There are several limitations in this study. Firstly, the study was designed as a single-arm as there is no standard of care for patients with histiocytic neoplasms globally. Secondly, the trial was carried out exclusively in a Chinese population, which restricts the generalisability of evidence to other ethnic or racial groups. Thirdly, there were only a few patients with conditions such as ECD and other histiocytic neoplasms, apart from LCH. Lastly, the current duration of follow-up for this study was relatively short. Mature median PFS and OS data will be updated in the future with longer follow-up.

This study showed that luvometinib resulted in durable responses in patients with histiocytic neoplasms of various subtypes and genotypes, and the safety profile was manageable. In summary, the clinical results suggested that luvometinib could be an alternative option for patients with histiocytic neoplasms, regardless of disease subtypes.

## Contributors

JL, XC, ZWu, ZWei, JG, XW, and HL designed the study. JL, XC, QZ, ZC, JM, HZ, LC, and LZ were responsible for recruiting patients and collecting data. ZWei, PH, and YZ analysed and interpreted data. JL and XW directly accessed and verified the underlying data. All authors reviewed and revised the manuscript. All authors had full access to all the data in the study and had final responsibility for the decision to submit for publication.

## Declaration of interests

XW, ZWei, JG, HL, and YZ are employees of Fosun Pharmaceutical Development Co., Ltd. ZWu and PH were employees of Fosun Pharmaceutical Development Co., Ltd at the time of the study and manuscript preparation. All other authors have no competing interests.

## Data Availability

The deidentified individual participant data that support the findings of this study are available from the corresponding author and sponsor upon reasonable request. Study protocol and statistical analysis plan are available in the Supplementary Material.

## Appendix A Supplementary data

Supplementary Materials

Statistical Analysis Plan

Clinical Study Protocol
