# Supplementary material for: Luvometinib in patients with Langerhans cell histiocytosis, Erdheim–Chester disease, and other histiocytic neoplasms: a single-arm, multicentre, phase 2 study
Source: eClinicalMedicine. 2025 Sep 17;88:103486. doi: 10.1016/j.eclinm.2025.103486 (PMC12481021; doi:10.1016/j.eclinm.2025.103486)
Supplement: Supplementary Materials [file mmc1.docx]

**Supplementary Appendix**

**Luvometinib in Patients With Langerhans Cell Histiocytosis, Erdheim-Chester Disease, and Other Histiocytic Neoplasms: A Single-Arm, Multicentre, Phase 2 Study**

Xin-xin Cao, Qi Zhu, Zhen Cai, Jie Ma, Hui Zhou, Long Chang, Lai-ping Zhong, Zhu-li Wu, Xingli Wang, Pu Han, Hongmei Lin, Zhen Wei, Jia-yan Guo, Yang Zheng, and Jian Li

**Table of content**

[Supplementary Figure 1. Dynamic changes of MAPK pathway variant allele frequency (VAF%) in cfDNA 2](#_Toc205374023)

[Supplementary Figure 2. Imaging results and change in the allele frequency of *BRAF* N486_P490del in cfDNA for one case 3](#_Toc205374024)

[Supplementary Table 1. Ethical approval number at each study site 4](#_Toc205374025)

[Supplementary Table 2. Efficacy endpoints assessed by investigator per PRC and RECIST 1.1 5](#_Toc205374026)

[Supplementary Table 3. Common TEAEs and TRAEs in patients treated with luvometinib 6](#_Toc205374027)

[Inclusion and exclusion criteria 7](#_Toc205374028)

[Positron emission tomography response criteria for FDG-PET/CT response assessments 9](#_Toc205374029)

[Definitions of efficacy endpoints 10](#_Toc205374030)

[Sample size calculation 11](#_Toc205374031)

# Supplementary Figure 1. Dynamic changes of MAPK pathway variant allele frequency (VAF%) in cfDNA

# Supplementary Figure 2. Imaging results and change in the allele frequency of *BRAF* N486_P490del in cfDNA for one case

**
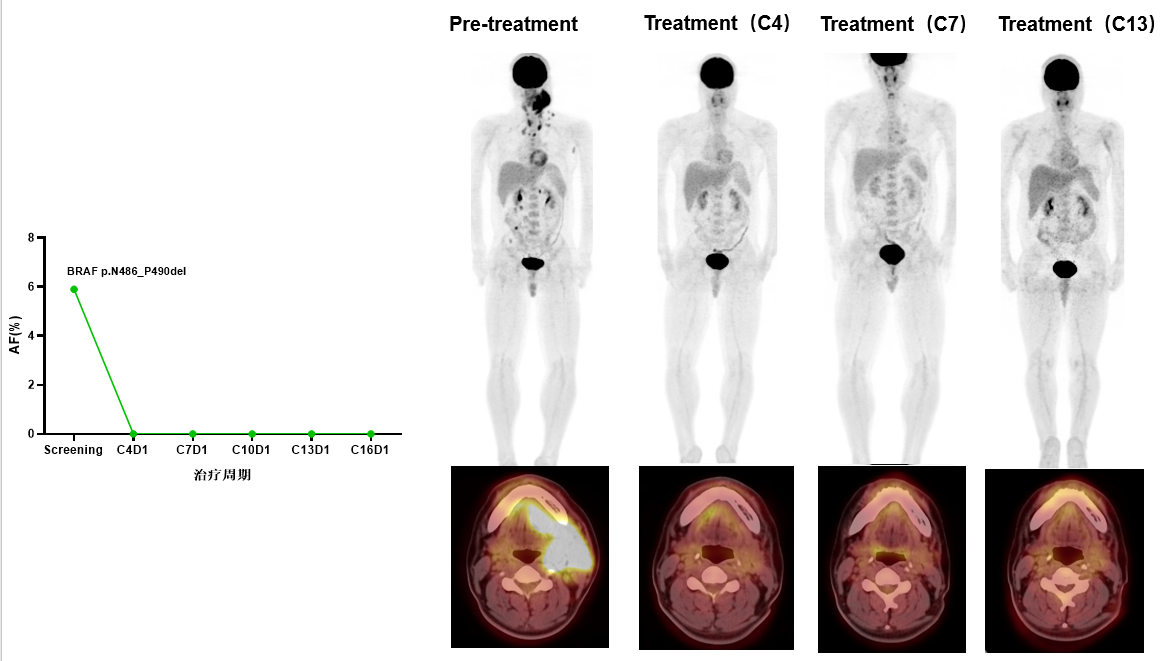
**

One enrolled patient diagnosed with LCH presented with subcutaneous nodule, left cervical and supraclavicular lymph node, and right ilium at baseline. Genetic testing confirmed the presence of *BRAF* N486_P490del in tumour tissues. 18FDG-PET/CT imaging revealed that after 4 cycles of treatment with luvometinib, the lesions disappeared and remained undetectable by cycle 13. The VAF% of *BRAF* N486_P490del in cfDNA was 6% at baseline, which subsequently became undetectable following three cycles of treatment.

# Supplementary Table 1. Ethical approval number at each study site

| Study site | Ethical approval number |
| --- | --- |
| Peking Union Medical College Hospital, Chinese Academy of Medical Sciences | KS2022085 |
| The First Affiliated Hospital of Zhejiang University School of Medicine | 2022伦审第（209）号 |
| The First Affiliated Hospital of Zhengzhou University | L2022-Y031-002 |
| West China Hospital of Sichuan University | 2022年临床试验（西药）审（210）号 |
| Hunan Cancer Hospital | 2022年快审[723]号 |
| Shanghai Ninth People’s Hospital, Shanghai Jiaotong University School of Medicine | SH9H-2022-C35-2 |
| Xiangya Hospital of Central South University | 伦审GCP第（202205117）号 |
| The Fourth Hospital of Hebei Medical University | 2022040 |

# Supplementary Table 2. Efficacy endpoints assessed by investigator per PRC and RECIST 1.1

| **Efficacy endpoint** | **PRC** | **RECIST 1.1** |
| --- | --- | --- |
| Evaluable patient numbers, n | 29 | 13 |
| Confirmed best overall response, n (%) |  |  |
| CMR/CR | 12 (41.4) | 0 |
| PMR/PR | 10 (34.5) | 6 (46.2) |
| SMD/SD | 5 (17.2) | 6 (46.2) |
| SMD/SD ≥24 weeks | 2 (6.9) | 5 (38.5) |
| PMD/PD | 2 (6.9) | 1 (7.7) |
| NE | 0 | 0 |
| ORR, n (%) | 22 (75.9) | 6 (46.2) |
| 95% CI | 56.5–89.7 | 19.2–74.9 |
| TTR, months |  |  |
| Median | 2.9 | 3.4 |
| DCR, n (%) | 27 (93.1) | 12 (92.3) |
| 95% CI | 77.2–99.2 | 64.0–99.8 |

ORR: the proportion of patients with confirmed CR+PR.

DCR: the proportion of patients with confirmed CR+PR+SD.

CBR: the proportion of patients with confirmed CR+PR+SD ≥24 weeks.

TTR: the time from the date of first dose to the first confirmed CR or PR (whichever was recorded first). Only patients who achieved a confirmed CR or PR during the study period were included in the TTR analysis.

IRC=independent review committee; ITT=Intent-to-Treat; ORR=overall response rate; CMR=complete metabolic response; PMR=partial metabolic response; SMD=stable metabolic disease; NE=not evaluable; ORR, objective response rate; CBR=clinical benefit rate; CI, confidence interval; DCR, disease control rate; TTR, time to response; PRC=positron emission tomography response criteria.

# Supplementary Table 3. Common TEAEs and TRAEs in patients treated with luvometinib

|  | **Patients included in safety analysis (n = 29)^a^** | | | |
| --- | --- | --- | --- | --- |
|  | **TEAEs** | | **TRAEs** | |
| **n (%)** | **Any grade** | **Grade ≥3** | **Any grade** | **Grade ≥3** |
| Upper respiratory tract infection | 15 (51.7) | 0 | 1 (3.4) | 0 |
| Alanine aminotransferase increased | 12 (41.4) | 0 | 11 (37.9) | 0 |
| Folliculitis | 11 (37.9) | 3 (10.3) | 11 (37.9) | 3 (10.3) |
| Rash | 11 (37.9) | 1 (3.4) | 10 (34.5) | 1 (3.4) |
| COVID-19 | 10 (34.5) | 0 | 0 | 0 |
| Peripheral oedema | 10 (34.5) | 0 | 10 (34.5) | 0 |
| Diarrhoea | 10 (34.5) | 0 | 10 (34.5) | 0 |
| Eczema | 8 (27.6) | 1 (3.4) | 7 (24.1) | 1 (3.4) |
| Creatine phosphokinase increased | 8 (27.6) | 2 (6.9) | 7 (24.1) | 1 (3.4) |
| Paronychia | 7 (24.1) | 0 | 7 (24.1) | 0 |
| C-reactive protein increased | 6 (20.7) | 0 | 5 (17.2) | 0 |
| Constipation | 6 (20.7) | 0 | 4 (13.8) | 0 |
| Aspartate aminotransferase increased | 6 (20.7) | 0 | 6 (20.7) | 0 |
| Thrombocytopenia | 5 (17.2) | 0 | 4 (13.8) | 0 |
| Hyperuricemia | 5 (17.2) | 0 | 3 (10.3) | 0 |
| Hypertriglyceridemia | 4 (13.8) | 3 (10.3) | 4 (13.8) | 3 (10.3) |

NOTE. Most common any-grade TEAEs in 15% or more of patients by systemic organ class preferred term and grade ≥3 TEAEs in more than one patient (>3.4%) are presented.

^a^1 patient with incorrect disease diagnosis was excluded from the analysis

# Inclusion and exclusion criteria

Inclusion criteria

1. Age >16 years.
2. Patients with histologically confirmed Langerhans cell histiocytosis (LCH), Erdheim-Chester disease (ECD), or other histiocytic tumours.
3. Can provide a sufficient number of histopathological sections or tumour tissue samples and peripheral blood for central laboratory testing of the following biomarkers: including but not limited to ERBB3, RAF-1, BRAF, ARAF, HRAS, KRAS, NRAS, MEK (MAP2K1 and MAP2K2) and other MEK upstream genes.
4. Patients with LCH were required to have lesions involving multiple systems (more than 1 system) or multiple lesions involving a single system (more than 1 lesion).
5. There were lesions evaluable based on PET response criteria (PRC).
6. Patients with newly treated or relapsed or refractory LCH, ECD or other histiocytic tumours can be included.
7. Expected survival was at least 3 months.
8. ECOG performance status was 0-2 points.
9. Able to understand and voluntarily sign written informed consent.
10. Premenopausal women who had a negative serum human chorionic gonadotropin (HCG) pregnancy test within 7 days before starting medication; did not need serum human chorionic gonadotropin (HCG) for women with surgical infertility or infertile women who were ≥1 year postmenopausal ( HCG) pregnancy test.
11. For patients of childbearing potential: Patients should agree to use effective contraception during treatment and for at least 90 days after the last dose of study treatment, and accept double barrier contraceptive methods, condoms, oral or injectable contraceptives, intrauterine devices and other contraceptive methods. Male patients should agree to refrain from donating sperm for at least 90 days after the last dose.
12. Adequate bone marrow function: without blood transfusion, blood products, or granulocyte colony-stimulating factor, the absolute value of neutrophils was ≥1.5×10^9^/L, haemoglobin was ≥90g/L, and platelets were ≥100×10^9^/L. Patients whose blood cell reduction caused by the disease itself was lower than these thresholds could be enrolled based on the comprehensive judgment of the investigator.
13. Adequate liver and kidney function: aspartate aminotransferase (AST), alanine aminotransferase (ALT) ≤ 2.5 × upper limit normal (ULN), if liver invasion occurred, ≤ 5 ×ULN; serum total bilirubin ≤1.5×ULN, in patients with Gilbert syndrome or liver invasion, ≤5.0×ULN; alkaline phosphatase (AKP) ≤2.5×ULN, in case of liver invasion, ≤10 ×ULN; serum creatinine ≤1.5×ULN. If serum creatinine was >1.5×ULN, creatinine clearance (CCr) ≥50mL/min (based on Cockcroft-Gault formula); albumin ≥3g/dL.
14. Coagulation function: international normalized ratio (INR) and activated partial thromboplastin time (APTT) ≤ 1.5ULN.

Exclusion criteria

1. Patients who had received one of the following prior treatments:
2. Received chemotherapy, targeted therapy, immunotherapy, biological therapy, experimental treatment, or herbal anti-tumour treatment for histiocytic tumours within 4 weeks or <5 half-lives (whichever was longer) before starting study drug treatment.
3. Had received strong inhibitors or inducers of CYP3A4, CYP2C8, or CYP2C9 within 14 days before starting study drug treatment, except for external use on the skin.
4. Used growth factors that promote platelet or white blood cell count or function within 7 days before starting study drug treatment.
5. Had received radiotherapy or surgery within 4 weeks before starting study drug treatment.
6. Patients who had participated in other interventional clinical trials within 4 weeks before starting study drug treatment.
7. Previous treatment with MEK 1/2 inhibitors.
8. High-dose chemotherapy and salvage stem cell transplantation (autologous stem cell transplantation) or allogeneic stem cell transplantation were performed within 90 days before enrolment. Patients receiving anti-Graft Versus Host Disease (GVHD) drugs after transplantation, such as cyclosporine, tacrolimus, or other drugs to prevent GVHD after bone marrow transplantation, could not participate in this trial.
9. Patients with brain tumours (intracranial masses) who had used anticoagulants within 7 days before starting study drug treatment.
10. Prednisone therapy <0.5 mg/kg/day (or equivalent doses of other corticosteroids) in the month prior to enrolment was permitted but must be discontinued 14 days before initiation of study medication. Patients with brain lesions receiving corticosteroids to treat cerebral oedema must maintain a stable dose for 14 days prior to enrolment. Hormone replacement therapy was permitted for patients with pituitary insufficiency due to primary disease involvement.
11. Patients who have a history of other malignant tumours or suffered from other malignant tumours at the same time (excluding cured non-melanoma basal cell carcinoma of the skin, carcinoma in situ of the breast or carcinoma in situ of the cervix, and other malignant tumours with no evidence of disease within 5 years).
12. Uncontrolled hypertension (with drug treatment): systolic blood pressure >160mmHg and/or diastolic blood pressure >100mmHg.
13. Patients who had difficulty swallowing, active digestive system diseases, malabsorption syndrome, or other conditions that affected the absorption of study drugs.
14. Eye examinations for past or current retinal vein occlusion (RVO), retinal pigment epithelial detachment (RPED), glaucoma and other significant abnormalities.
15. Interstitial pneumonia, including clinically significant radiation pneumonitis. Interstitial pneumonia due to primary lung involvement was excluded.
16. Heart function or combined diseases that met one of the following conditions were excluded:
17. During the screening period, three 12-lead electrocardiogram (ECG) measurements were performed at the research centre, and the average of the three times was calculated according to the QTcF formula of the instrument. QTcF>470 milliseconds; for patients with risk of prolonged QTcF, such as uncorrectable hypokalaemia, hereditary long QT syndrome; or receiving drugs that prolong the QTcF interval (mainly Class Ia, Ic, and III antiarrhythmic drugs). For drugs that potentially prolong the QTcF interval.
18. Congestive heart failure classified as New York Heart Association (NYHA) grade ≥ 3.
19. Clinically significant arrhythmias, including but not limited to complete left bundle branch conduction abnormality, II degree atrioventricular block.
20. Known to be complicated by clinically significant coronary heart disease, cardiomyopathy, and severe valvular disease.
21. Echocardiography showed that the left ventricular ejection fraction (LEFV) was <50%.
22. With active bacterial, fungal or viral infection, including active hepatitis B (positive hepatitis B virus surface antigen and hepatitis B virus DNA exceeding 1000 IU/ml or meeting the research centre’s diagnostic criteria for active hepatitis B infection) or hepatitis C (hepatitis C viral RNA positive), human immunodeficiency virus infection (HIV positive).
23. Pregnant or lactating women.
24. Known allergy to study drugs, other MEK1/2 inhibitors or other excipients.
25. Clinically significant conditions identified by the investigator that would hinder study participation or prevent adherence to safety requirements.

# Positron emission tomography response criteria for FDG-PET/CT response assessments

| Response classification | Criteria based on the sum of standardized uptake values (SUVs) of up to 5 target lesions |
| --- | --- |
| Complete Metabolic Response (CMR) | All lesions (target and non-target lesions) demonstrated SUV_max_ normalization, which was either equal to or lower than the background (SUV_max_ of liver/mediastinal blood pool for extracranial/skull lesions, , and SUV_max_ of white matter for intracranial/skull lesions) |
| Partial Metabolic Response (PMR) | The sum of SUVs for all target lesions decreased by ≥50% compared to baseline |
| Progressive Metabolic Disease (PMD) | The sum of SUVs for all target lesions increased by ≥50% from the lowest point, with a minimum absolute increase of 3 units for each target lesion (for example, from SUV 3 to SUV 6); or the presence of new evaluable lesions that were considered indicative of clear disease progression. |
| Stable Metabolic Disease (SMD) | Did not meet other criteria |

Up to 5 target lesions were selected, with no more than 2 lesions for each organ. Target lesions must meet the following criteria: the SUV_max_ for extracranial or non-skull lesions must be >1.5 times the sum of liver mean standard uptake value (SUV_mean_) and 2 times the liver SUV_mean_ standard deviation; if liver was involved, SUV_max_ must be >2 times of the sum of SUV_mean_ and 2 times the SUV_mean_ standard deviation for the mediastinal blood pool. The SUV_max_ of intracranial or skull lesions must be >1.5 times the sum of SUV_mean_ and 2 times the standard deviation of the SUV_mean_ of the brain white matter.

SUV was calculated using the following methods:

1. For extracranial/non-skull target lesions, the SUV was calculated as target lesion SUV_max_ – liver SUV_max_ (if the liver was affected by a lesion, this was calculated as target lesion SUV_max_ – mediastinal blood pool mean SUV_max_)
2. For intracranial/skull target lesions, the SUV was calculated as the target lesion SUV_max_ – brain white matter SUV_max_.
3. If the result was negative, it was defined as 0.

# Definitions of efficacy endpoints

| Endpoint | Evaluation criterion | Definition |
| --- | --- | --- |
| ORR | PRC | Proportion of patients who achieved CMR and PMR |
| ORR | RECIST 1.1 | Proportion of patients who achieved confirmed CR and PR |
| DCR | PRC | Proportion of patients who achieved confirmed CMR, PMR, and SMD |
| DCR | RECIST 1.1 | Proportion of patients who achieved confirmed CR, PR, and SD |
| CBR | PRC | Proportion of patients with confirmed CMR, PMR, and SMD lasting ≥24 weeks |
| CBR | RECIST 1.1 | Proportion of patients with confirmed CR, PR, and SD lasting ≥24 weeks |
| TTR | PRC | Time from the first dose of study treatment to the first occurrence of a CMR or PMR |
| TTR | RESIST 1.1 | Time from the first dose of study treatment to the first occurrence of a CR or PR |
| PFS | PRC | Time from the first dose of study treatment to progressive metabolic disease (PMD) or death |
| PFS | RECIST 1.1 | Time from the first dose of study treatment to PD or death |
| OS | - | Time from the first dose of study treatment to death from any cause |

CBR, clinical benefit rate; CMR, complete metabolic response; CR, complete response; DCR, disease control rate; OS, overall survival; ORR, overall response rate; PFS, progression-free survival; PMR, partial metabolic response; PR, partial response; SD, stable disease; SMR, stable metabolic response; TTR, time to response.

# Sample size calculation

A total of 28 patients would be enrolled in this study; assuming a drop out rate of 10%, there would be 25 evaluable patients.

The assumed ORRs and 95% CIs are as follows.

| Number of patients with a response | 18 | 19 | 20 | 21 | 22 |
| --- | --- | --- | --- | --- | --- |
| ORR | 72% | 76% | 80% | 84% | 88% |
| 95% CI | (50.6, 87.9) | (54.9, 90.6) | (59.3, 93.2) | (63.9, 95.5) | (68.8, 97.5) |
| 95% confidence interval width | 37.3 | 35.7 | 33.9 | 31.6 | 28.7 |
