# Supplementary material for: Luvometinib in patients with Langerhans cell histiocytosis, Erdheim–Chester disease, and other histiocytic neoplasms: a single-arm, multicentre, phase 2 study
Source: eClinicalMedicine. 2025 Sep 17;88:103486. doi: 10.1016/j.eclinm.2025.103486 (PMC12481021; doi:10.1016/j.eclinm.2025.103486)
Supplement: Statistical Analysis Plan [file mmc2.pdf]

# Statistical Analysis Plan

Investigational Product: FCN-159 Tablets

Protocol Name: A multicenter, open-label, single-arm Phase II study to evaluate the efficacy, safety, and pharmacokinetic characteristics of FCN-159 in patients with histiocytic tumors

Clinical stage: Phase II

Sponsor: [redacted]

Protocol Version/Date: version 2.1/09Mar2023

SAP Version/Date: version 1.0/30 AUG 2023

Author of this plan: [redacted]

# Statistical Analysis Plan Signature Page

This document has been approved and signed as follows:

| Signatory | Signature |
|---|---|
| Author | [redacted] Project Responsibilities: project Statistician |
| | Signature<br>Date |
| Reviewer | [redacted] Project Responsibilities: review Statistician |
| | Signature<br>Date |
| | [redacted] Project Responsibilities: principal Statistical Programmer |
| | Signature<br>Date |
| | [redacted] Project Responsibility: medical Monitoring |
| | Signature<br>Date |
| Approved by | [redacted] Project Responsibilities: sponsor Representative |
| | Signature<br>Date |

# TABLE OF CONTENTS

Table of Contents ..... 3
List of Abbreviations ..... 5
1 Overview ..... 7
2 Study Objectives ..... 7
2.1 Primary Objectives ..... 7
2.2 Secondary Objectives ..... 7
2.3 Exploratory Objectives ..... 7
3 Study Endpoints ..... 7
3.1 Primary Endpoints ..... 7
3.2 Secondary Endpoints ..... 7
3.3 Exploratory Endpoints ..... 8
4 Study Design ..... 8
4.1 Overall Design ..... 8
4.2 Sample Size ..... 8
5 ANALYTICAL DEFINITIONS AND RULES ..... 9
5.1Dividing of Analysis Sets ..... 9
5.2 Handling Principles of Missing Dates ..... 9
5.3 Definitions and Derivatives ..... 12
6 Statistical Analysis Methods ..... 13
6.1Subject Characteristics ..... 14
6.1.1 Subject Disposition ..... 14
6.1.2 Protocol deviations ..... 14
6.1.3 Demographics and Baseline Characteristics ..... 15
6.1.4Prior Anti-tumor Therapies ..... 15
6.1.5Prior/concomitant Medications ..... 15
6.1.6Prior/concomitant non-drug therapies ..... 16
6.1.7 Subsequent anti-tumor therapy ..... 16
6.2 Efficacy Analysis ..... 16
6.2.1 Primary Endpoints ..... 16
6.2.2 Secondary Endpoints ..... 17
6.2.3 Exploratory Endpoints ..... 22
6.3 Safety Analysis ..... 23
6.3.1 Study Drug Exposure and Compliance ..... 23
6.3.2 Adverse Events ..... 23
6.3.3 Laboratory Tests ..... 26
6.3.4 Vital Signs ..... 26
6.3.5 Physical Examination ..... 27
6.3.6 12Lead ECG ..... 27
6.3.7 Other Safety Tests ..... 27
6.4 Pharmacokinetic Analyses ..... 28
6.5 Interim Analyses ..... 28

7 Amendments to Planned Analyses in the Protocol ........ 28
8 References ........ 28
9 REVISION RECORD ........ 30

## LIST OF ABBREVIATIONS

| Abbreviations | Chinese translation |
|---|---|
| AE | Adverse Events |
| ATC | Anatomical Therapeutic Chemical Classification |
| BMI | Body mass index |
| BQL | Below lower limit of quantification |
| CBR | Clinical Benefit Rate |
| CI | Confidence interval |
| CMR | Complete metabolic response |
| CR | Complete response |
| CRF | Case Report Form |
| DCR | Disease control rate |
| DOR | Duration of response |
| ECD | Erdheim-Chester disease |
| ECG | Electrocardiogram |
| ECOG | Eastern Cooperative Oncology Group |
| IRC | Independent Review Committee |
| ITT | Intent-to-treat Analysis Set |
| LCH | Langerhans cell histiocytosis |
| mITT | Modified Intent-to-Treat Analysis Set |
| NCI-CTCAE | National Common Toxicity Criteria for Adverse Events in Cancer Research |
| ORR | Objective response rate |
| OS | Overall survival |
| PFS | Progression-free survival |
| PK | Pharmacokinetics |
| PMD | Increased metabolism of lesions |
| PMR | Partial metabolic response |
| PopPK | Population Pharmacokinetics |
| PR | Partial response |
| PRC | PET Response Assessment Criteria |
| PT | Preferred Name |

| | |
|---|---|
| QOL | Quality of life |
| RECIST | Response Evaluation Criteria in Solid Tumors |
| SAE | Serious Adverse Events |
| SAP | Statistical Analysis Plan |
| SD | Stable disease |
| SMD | Stable metabolic response |
| SUV | Standard Uptake Value |
| TEAE | Treatment-emergent adverse events |
| TTP | Time to progression |
| TTR | Time to Response |

# 1 OVERVIEW

This statistical analysis plan is developed in accordance with the protocol "A multicenter, open-label, single-arm Phase 2 study to evaluate the efficacy, safety and pharmacokinetic profile of FCN-159 in patients with histiocytic tumors"(Protocol No.: fCN-159-005, Version 2.1 dated 09 MAR 2023) and described the statistical methods required for data analysis and summary. This statistical analysis plan focuses only on the efficacy analysis and safety analysis part of the study. Population PK analyses will not be included in this statistical analysis plan and will be attached to the clinical trial report in the form of an independent analysis plan.

# 2 STUDY OBJECTIVES

## 2.1 Primary Objective

To evaluate the efficacy of FCN-159 tablets in patients with Langerhans cell histiocytosis (LCH), Erdheim-Chester disease (ECD), and other histiocytic tumors.

## 2.2 Secondary Objective

To evaluate the safety of FCN-159 tablets in patients with LCH, ECD and other histiocytic tumors.

To further evaluate the efficacy of FCN-159 tablets in patients with LCH, ECD, and other histiocytic tumors in addition to ORR assessed by IRC based on PET Response Criteria (PRC).

To further evaluate the PK profile of FCN-159 tablets.

To evaluate the improvement in quality of life of patients with LCH, ECD, and other histiocytic tumors compared with baseline examinations with FCN-159 tablets.

## 2.3 Exploratory Objectives

To analyze the relationship between MAPK pathway gene mutation and therapeutic effect.

# 3 STUDY ENDPOINTS

## 3.1 Primary endpoint

Objective response rate (ORR) assessed by an Independent Review Committee (IRC) based on PET Response Assessment Criteria (PRC).

## 3.2 Secondary endpoints

ORR assessed by the investigator based on PRC response assessment criteria;

ORR, DCR, CBR, TTR,

PFS□OS□

To evaluate the frequency and severity of adverse events, serious adverse events, deaths, and safety test abnormalities (e.g., laboratory tests, vital signs, physical examinations, electrocardiograms, ECOG, etc.) according to the National cancer institute common terminology criteria for adverse events (NCI-CTCAE) version 5.0; proportion of patients with dose modification or discontinuation due to drug toxicity.

Changes in Quality of Life (QOL) EORTC-QLQ-C30 (Version 3.0).

Population PK parameters of FCN-159 tablets.

## 3.3 Exploratory Endpoints

To evaluate MAPK pathway gene mutations and to analyze the correlation with efficacy.

# 4 STUDY DESIGN

## 4.1 Overall design

This is a single-arm, open-label, multicenter Phase 2 clinical study of rare diseases. A total of approximately 28 patients with LCH, ECD, and other histiocytic tumors will be included, and the diagnosis will be reviewed by the central pathology of the leading unit. FCN-159 will be administered at a dose of 8 mg/day orally once daily as a single agent in a 28-day treatment cycle until disease progression, death, intolerable toxicity, patient withdrawal of consent, or end of study.

Tumor response will be evaluated by the IRC and the investigator once at screening, at the end of 3rd, 6th, 12th treatment cycles (± 7 days), and at the end of treatment according to PET response assessment criteria (PRC). Patients evaluated as CMR or PMR had confirmed response by repeated assessments at least 4 weeks later. Tumor response will be evaluated once at screening, at the end of 3rd, 6th, 9th, 12th treatment cycles (± 7 days), and at the end of treatment by IRC and investigator according to RECIST v1.1. If the imaging results are considered to be consistently stable by the Principal Investigator after 12 treatment cycles, tumor assessments will be performed every 4 treatment cycles (± 14 days) according to RECIST 1.1, if the imaging results do not reach sustained stability in the opinion of the investigator after 12 treatment cycles, tumor assessments will be performed every 3-6 treatment cycles (± 14 days) according to PRC criteria, and tumor assessments will be performed every 3 treatment cycles (± 7 days) according to RECIST 1.1. FCN-159 tablets will be treated for up to 2 years. Quality of life and MAPK pathway gene mutation detection will be assessed at the same time as RECIST 1.1 tumor assessment.

## 4.2 Sample Size

Approximately 25 evaluable patients are planned to be enrolled in the study, taking into account a

dropout rate of 10%, and a total of approximately 28 patients will be enrolled. Assuming that the proportion of patients achieving CMR and PMR is 80%, the following table presents the ORR and 95% confidence interval corresponding to the different number of responders.

ORR with 95% CI in relation to number of responders in 25 evaluable patients.

| Number of responders | 18 | 19 | 20 | 21 | 22 |
|---|---|---|---|---|---|
| Estimated ORR | 72% | 76% | 80% | 84% | 88% |
| 95% confidence interval (%) | (50.6, 87.9) | (54.9, 90.6) | (59.3,93.2) | (63.9, 95.5) | (68.8,97.5) |
| 95% Confidence Interval Width (%) | 37.3 | 35.7 | 33.9 | 31.6 | 28.7 |

# 5 ANALYSIS DEFINITIONS AND RULES

## 5.1 Analysis Set Division

Intent-to-Treat (ITT): patients who signed informed consent and took at least one dose of FCN-159 tablets.

Modified Intent-to-Treat (mITT): patients who took at least one dose of FCN-159 tablets, had baseline tumor assessment data and at least one post-baseline tumor assessment data, and had no major protocol violations.

Safety Analysis Set: any patient who took at least one dose of FCN-159 tablets and had at least 1 safety assessment.

PK analysis set: includes patients who have taken at least one dose of FCN-159 tablets and have at least 1 PK blood sample collected as planned and have plasma concentration data of study drug.

## 5.2 Handling Principles for Missing Dates

Unless specifically stated below, any missing data will be treated as missing without imputation of any hypothetical values. All imputation will only be used for judgment or derivation, and the data listings will be listed according to the actual situation filled in the CRF and will not be imputed.

**Missing adverse event start date: the following three scenarios:**

1 The start date of adverse event (AE) is completely missing;

2 The year of the start date is known and the same as the date of the first dose of study drug;

3 The year and month of the start date is known and the same as the date of the first dose of study drug;

The start date of the AE will be imputed using the date of first dose of study drug.

Any other missing "day" will be imputed as "1 day" and any "month" missing will be imputed as "January".

**Missing Adverse Event End Date:**

The AE end date is missing, and if only "day" is missing or only "month and day" missing, the imputed date is not later than the date of the subject ’ s death, otherwise the death date will be used for imputation. The remaining missing cases will not be imputed.

If only "day" is missing, impute as the last day of the month;

If "month and day" is missing, impute as "December 31" ;

**Missing date of first diagnosis of tumor history: if only day is missing (year/month only), replace with 15 days; if day and month are missing and only year, replace with July 1; if the imputed date is not later than the start date of prior tumor therapy history, otherwise impute with the start date; the remainder will be considered missing.**

**Missing dates of prior/concomitant medications: refer to the table below. In cases where a drug cannot be defined as prior, concomitant, or subsequent, the drug will be categorized by worst case, i.e., defined as pooling.**

| **Start date** | **End date** | **Imputation Rules** | **Prior/Combined/Subsequent Judgment Rules** |
|---|---|---|---|
| Known | Known | Not applicable. | If the end date is < the date of the first dose of study drug, it is defined as "prior" ;<br>Defined as "pooled" if end date > = date of first dose of study drug and start date < = end of observation (i.e. date of last dose + 30 days, or start of new anti-tumor therapy, whichever occurs first); |
| Known | Partially missing | If only "day" is missing, the last day of the month will be imputed; the missing "month and day" will be imputed as "31 December". | |

| Start date | End date | Imputation Rules | Prior/Combined/Subsequent Judgment Rules |
|---|---|---|---|
| Partially missing | Known | If only "day" is missing, the first day of the month will be imputed; the missing "month and day" will be imputed as "January 1". | If end date > = date of first dose of study drug and start date > observation end date, it is defined as "subsequent". |
| Partially missing | Partially missing | Start date: if only "day" is missing , the first day of the month will be imputed; the missing "month and day" imputed as "January 1". End date: if only "day" is missing , the last day of the month will be imputed; the missing "month and day" imputed as "31 December". | |
| | | | |
| Known | Completely missing | End date not imputed. | Defined as "pooled" if start date < = observation end date; If start date > observation end date, it is defined as "Subsequent". |
| Partially missing | Completely missing | Start date: if only "day" is missing , the first day of the month will be imputed; the missing "month and day" imputed as "January 1". End date will not be imputed. | |
| | | | |
| Completely missing | Known | Start date will not be imputed. | If the end date is < the date of the first dose of study drug, it is defined as |

| Start date | End date | Imputation Rules | Prior/Combined/Subsequent Judgment Rules |
|---|---|---|---|
| Completely missing | Partially missing | Start date will not be imputed. End date: only "day" is missing and imputed as the last day of the month; missing "month and day" imputed as "31 December". | "prior" ; Defined as "pooled" if end date > = date of first dose of study drug. |
| Completely missing | Completely missing | None will not be imputed. | All defined as "pooled" |

Handling of AEs with missing relationship to study drug: if the relationship to study drug is missing, the relationship of the adverse event to study drug will be considered as possibly related in the correlation tables and summarized.

Missing safety endpoints: in general, missing safety endpoint values will not be handled. Adverse events are missing in severity and will be considered Grade 3 for pooled analysis.

**Missing date of death:** when derived time-to-event endpoint variables are handled as follows, if only "day" is missing, impute as "1 day" ; if "month and day" is missing, impute as "January 1" ; if the imputed date is not earlier than the latest follow-up date of assessment/examination/awareness of survival recorded in the subject ' s entire EDC database, the latest follow-up date of assessment/examination/knowledge of survival + 1 will be used as the date of death (including when the date of death is missing in "years").

## 5.3 Definition and Derivation

**Baseline**: baseline is defined as the last non-missing measurement before the subject takes the study drug, if not otherwise specified. When the last non-missing measurement cannot be determined by time and the corresponding scheduled visit measurement is not missing, the corresponding scheduled visit measurement is used. If the last non-missing measurement coincides with the start date of study drug use, the measurement will be considered pre-baseline, but adverse events and drug therapies that start on the start date of study drug will be considered post-baseline.

**Change from Baseline = Measurements at Visit X-Baseline.**

Time to onset of tumor assessments or duration of event endpoints are based on the actual date of imaging rather than the date of assessment/reading. Tumor assessment data recorded at all scheduled and

unscheduled visits will be summarized.

**Study Follow-up**: defined as the time from the date of the first dose of study drug to the end of the study date (for subjects who have withdrawn from the study prior to the data cutoff date) or the data cutoff date (for subjects still on treatment/follow-up).

**Time from first diagnosis to enrollment (months)** = (date of first dose of study drug-date of first diagnosis + 1) ÷ 30.4375 to 1 decimal place.

**Study Days**: is the number of days when the response assessment is used relative to the reference date. The date of the first dose of study drug will be used as the reference date, and the reference date will be recorded as Day 1. Study days will be calculated as follows:

If assessment/examination date ≥ reference date: study day (day) = assessment/examination date – reference date + 1;

If assessment/examination is before the reference date: study day (day) = assessment/examination date – reference date.

If the assessment/examination date is partially or completely missing, the date will appear partially or completely missing in the listings, while the study day and any corresponding duration will be estimated according to the rules for handling missing dates.

Interval (weeks) = Study days (days) ÷ 7 to 1 decimal place;

Interval (months) = Study days (days) ÷ 30.4375 to 1 decimal place;

Interval (years) = Study days (dayss) ÷ 365.25 to 1 decimal place.

Unless otherwise specified, variables in the interval class will be calculated in months.

# 6 STATISTICAL ANALYSIS METHODS

Descriptive statistical analysis will be predominant used in this study. All statistical analyses will be performed using SAS version 9.4 or higher.

In general, continuous variables will be quantitatively described using the number of subjects, mean, median, Q1, Q3, standard deviation, minimum and maximum; categorical variables will be qualitatively described using frequencies and percentages for each category, unless otherwise specified, and percentages will be calculated using the number of analysis sets as the denominator. Unless otherwise stated, all confidence intervals (CIs) will have two-sided 95% confidence.

Unless otherwise specified, all postdose unscheduled visits will not be included in the statistical

analysis by visit but will be included for the calculation of maximum postbaseline severity or maximum change from baseline. Data from unscheduled visits will be presented as a listing.

The number of decimal places for statistical analysis will be retained as appropriate according to the following rules:

| Statistics | Decimal places |
|---|---|
| Min, Max | Same digits as raw data |
| Arithmetic mean, geometric mean, median, Q1, Q3, 95% CI | 1 more decimal place than raw data |
| Standard Deviation | 2 more decimal places than raw data |
| Number | 0 |
| Percentage | 1 |
| P-value | 4, when less than 0.0001, expressed as < 0.0001 |

Note: derived data are rounded to two decimal places except for special instructions and displayed as two significant figures when values are less than 0.01.

## 6.1 Subject Characteristics

### 6.1.1 Subject Disposition

All subjects who sign informed consent will be included in the analysis.

The number of subjects screened, the number of subjects who failed screening, and the reasons for screening failure will be described, and subjects who failed screening will not be included in other analyses.

The number of subjects enrolled, received study drug, discontinued from treatment, discontinued from the study, as well as the reason for discontinuation, reason for discontinuation from the study, and the study follow-up time (months) will be summarized, described and tabulated. The number of subjects in each analysis set will be summarized and the disposition for each analysis set will be tabulated.

### 6.1.2 Protocol deviations

Protocol deviations will be categorized as minor and major. At a data review meeting prior to database lock, the investigator and the sponsor will assess and agree on protocol deviations as "minor" or "significant". Final major protocol deviations will be listed in detail and documented prior to database lock.

Major protocol deviations will be summarized for subjects based on the analysis of enrolled subjects and details of all major protocol deviations will be presented in a listing form.

### 6.1.3 Demographics and Baseline Characteristics

The following demographic and baseline characteristics will be tabulated based on the ITT and mITT analyses, respectively, and all demographic and baseline characteristics data will be presented in tabular form.

Demographics: including age, sex, ethnicity, smoking status, smoking amount, alcohol consumption status, alcohol consumption, height (cm), weight (kg), BMI (kg/m2), and baseline ECOG score.

Past medical history (other than neoplasms): the subject ' s past medical history other than tumor will be coded using the Medical Dictionary for Regulatory Activities (MedDRA 26.0 or later) and summarized by system organ class (SOC) and preferred term (PT).

Tumor history: including time from first diagnosis to enrollment (months), time from last treatment of disease under study to enrollment (months), tumor diagnosis name, oncology classification, site of central invasion and central invasion, and MAPK pathway-related gene mutations.

### 6.1.4 Prior anti-tumor therapy

Based on ITT and mITT analyses.

History of prior tumor drug therapy: number of subjects who have received at least one tumor drug therapy, number of prior lines of drug therapy, type of drug therapy, and reason for end of drug treatment.

Prior tumor radiotherapy history: descriptive statistics will be performed for the number of subjects who have received at least one prior tumor radiotherapy.

Prior tumor surgery/procedure history: descriptive statistics will be performed for the number of subjects who have undergone at least one tumor surgery for the purpose of surgery/procedure.

Prior anti-tumor therapy (including tumor drug therapy history, radiotherapy history, tumor surgery/procedure history) data for all subjects will be presented in a listing form.

### 6.1.5 Prior/concomitant medication

Based on ITT and mITT analyses.

Prior/concomitant medications will be coded using the World Health Organization Drug Dictionary (1 September 2022 or higher) and summarized categorically by ATC2 and PT, and the number and percentage of subjects will be calculated.

Prior medications will be defined as medications that stopped before the date of the first dose of study

drug, and concomitant medications will be defined as medications taken while on study drug treatment (within 30 days after the first dose of the last dose, or before the initiation of new anti-tumor therapy). Specific judgment rules for prior/concomitant medications are detailed in the section "Principles for Handling Missing Dates".

Prior/concomitant medications will be listed for all subjects.

### 6.1.6 Prior/concomitant non-drug therapies

Based on ITT column listings.

Prior/concomitant non-drug therapies will be listed for all subjects.

### 6.1.7 Subsequent anti-tumor therapy

Based on ITT column listings.

Subsequent anti-tumor therapies for all subjects will be presented in a listing form.

## 6.2 Efficacy Analysis

Efficacy endpoints will be analyzed based on ITT and mITT, with mITT as the primary efficacy analysis set. Tumor assessment and survival follow-up data for all enrolled subjects will be presented in a listing form.

### 6.2.1 Primary endpoint

#### 6.2.1.1 Objective Response Rate (ORR) Based on PRC Assessed by IRC

Objective response rate (ORR) assessed by the Independent Review Committee (IRC) based on PET Response Assessment Criteria (PRC) is the primary efficacy endpoint specified in the protocol. Objective response rate (ORR) is defined as the proportion of patients achieving confirmed CMR and PMR as assessed by the IRC based on the PRC response evaluation criteria.

The order of best response will be : complete metabolic response (CMR) > partial metabolic response (PMR) > stable metabolic response (SMD) > increased lesion metabolism (PMD)/not evaluable (NE). Patients evaluated as CMR or PMR will confirme response by repeated assessments at least 4 weeks later. When the SMD is considered to be the best overall response, it needs to be evaluated for a minimum SMD duration criterion (6 weeks).

Table 1 Response Confirmation Judgment Rules

| Confirmed Response by CMR and PMR | | |
|---|---|---|
| Response at first | Efficacy at Subsequent | Efficacy assessment |

| time point | Time Points | |
|---|---|---|
| CMR | CMR | CMR |
| CMR | PMR | SMD, PMD or PMR *<br>In general, if the criteria for the shortest SMD period are met at the first time point, it is determined as SMD, otherwise it is PMD<br>PMR will be determined only if CMR should be modified to PMR at the first point of clinical re-evaluation. |
| CMR | SMD | SMD if defined criteria for minimum SMD duration are met, otherwise PMD |
| CMR | PMD | SMD if defined criteria for minimum SMD duration are met, otherwise PMD |
| CMR | NE | SMD if defined criteria for minimum SMD duration are met, otherwise NE |
| PMR | CMR | PMR |
| PMR | PMR | PMR |
| PMR | SMD | SMD |
| PMR | PMD | SMD if defined criteria for minimum SD duration are met, otherwise PMD |
| PMR | NE | SMD if defined criteria for minimum SD duration are met, otherwise NE |

Note: the minimum duration of SMD is 6 weeks; the duration of SMD will be calculated as: time to response assessment at the first time point-date of first dose + 1 (days).

If a tumor is assessed as PMR again after ≤ 2 tumor assessments (including NE) are missing, it can be used as a confirmed PMR, otherwise it cannot be used as a confirmed PMR; an SMD (i.e., PMR-SMD-PMR) can be used as a confirmed PMR when two tumor assessments are PMRs, otherwise it cannot be considered as a confirmed PMR.

After confirmation of response response, the best response assessed by tumor at all time points will be taken as the best overall response.

The number and percentage of confirmed objective responses (CMR and PMR) will be calculated; the best response evaluation will be determined based on the efficacy evaluation results of each cycle, and the 95% confidence interval (CI) of ORR will be calculated based on the Clopper-Pearson method.

A waterfall plot of the best percentage change from baseline in the sum of SUV of target lesions and a spider plot of the percentage change from baseline in the sum of SUV of target lesions will be plotted.

The analysis methods for unconfirmed objective response (CMR and PMR) are the same as above.

### 6.2.2 Secondary endpoints

#### 6.2.2.1 Objective Response Rate (ORR)

ORR based on RECIST 1.1 response evaluation is defined as the proportion of patients achieving

confirmed CR and PR based on RECIST 1.1 response evaluation.

ORR assessed by the investigator based on PRC and ORR assessed by IRC and investigator per RECIST 1.1 will be assessed using the same analytical method as the primary efficacy evaluation.

The best order of response based on RECIST 1.1 will be : complete response (CR) > partial response (PR) > stable disease (SD) > no evaluable disease (NED) > progressive disease (PD) > not evaluable (NE).

The summary of best overall response and ORR based on RECIST 1.1 will be analyzed using subjects with baseline target lesion data.

A waterfall plot of the best percentage change from baseline in the sum of diameters of target lesions and a spider plot of the percentage change from baseline in the sum of diameters of target lesions will be plotted.

#### 6.2.2.2 Disease Control Rate (DCR) and Clinical Benefit Rate (CBR)

Disease control rate (DCR) is defined as the proportion of patients who meet confirmed CMR + PMR + SMD as assessed by PRC response evaluation criteria and the proportion of patients who meet confirmed CR + PR + SD according to RECIST v1.1 response evaluation criteria, respectively.

Clinical benefit rate (CBR) is defined as the proportion of patients with confirmed CMR + PMR + SMD for ≥ 24 weeks as assessed by PRC criteria and confirmed CR + PR + SD for ≥ 24 weeks as assessed by RECIST v1.1 Response Evaluation Criteria.

DCR and CBR assessed by IRC/investigator: percentage of DCR/CBR and its two-sided 95% confidence interval will be calculated according to the Clopper-Pearson method, respectively.

Summaries of DCR and CBR based on RECIST 1.1 will be analyzed using subjects with baseline target lesion data.

#### 6.2.2.3 Progression-free survival (PFS)

Progression-free survival (PFS) is defined as the time from the first dose of study drug until disease progression (PMD or PD) or death, whichever occurs first.

Patients without an event (no progression or death) will be censored at the date of the last tumor assessment. If other anti-tumor therapy is received prior to disease progression, the previous radiographic date of the other anti-tumor therapy will be used as the censoring date. The specific progression and censoring definitions of PFS are shown in the following table:

| PFS status | PFS Events and Censoring Description | PFS Events and Censoring Dates |
|---|---|---|
| Censored | No baseline tumor assessments and no deaths reported prior to new anti-tumor therapy | Date of first dose |
| Censored | No postbaseline tumor assessments and no deaths reported prior to new anti-tumor therapy | Date of first dose |
| Event | No postbaseline tumor assessments and no more than 2 tumor assessments interval between the date of death and the date of first dose | Date of Death |
| Censored | No disease progression or death prior to new anti-tumor therapy or withdrawal from the trial before the start of new anti-tumor therapy, with no evidence of disease progression or death | Date of last imaging prior to start of new anti-tumor therapy |
| Event | Disease progression/death documented at or between adjacent scheduled visits (including missing 1 scheduled visit) | Date of first documentation of disease progression/date of death, whichever occurs first |
| Censored | Death or progression after ≥ 2 consecutive missing scheduled visits | Date of last imaging prior to consecutive missing |
| Note: tumor assessments will be performed at screening, at the end of treatment cycles 3, 6, 12 (± 7 days), and at the end of treatment based on PRC criteria per protocol. Therefore, the criterion for missing ≥ 2 consecutive scheduled visits is: maximum interval between two swelling assessments = (3 + 6) × 28 days/Treatment Period + 2 × 7 days = 266 days. If, in the opinion of the investigator, the imaging results do not reach sustained stability after 12 treatment cycles, tumor assessments will be performed every 3-6 treatment cycles (± 14 days) according to PRC criteria, with a maximum interval between the two tumor assessments = (6 + 6) × 28 days/treatment period + 2 × 7 days = 350 days<br>Tumor assessments will be performed every 3 treatment cycles (28-day treatment cycle) prior to 12 treatment cycles (±7 days) with a maximum interval of 2 ×3 ×28 days/treatment period + 2 ×7 days = 182 days based on RECIST 1.1 criteria. After 12 treatment cycles, the Principal Investigator considered the imaging results to be consistently stable, and tumor assessments will be performed every 4 treatment cycles (±14 days) according to RECIST 1.1, then the maximum interval between the two tumor assessments = 2 ×4 ×28 days/treatment period + 2 ×7 days = 238 days. After 12 treatment cycles, the investigator considered the imaging results to be not consistently stable, and tumor assessments will be performed every 3 treatment cycles (±7 days) according to RECIST 1.1, with a maximum interval between the two tumor assessments = 2 ×3 ×28 days/treatment period + 2 ×7 days = 182 days. | | |

PFS per PRC criteria or RECIST 1.1 as assessed by IRC/investigator: PFS curves will be constructed using the Kaplan-Meier method to estimate median PFS and its 95% confidence interval will be estimated using the Brookmeyer and crowley method. Progression-free survival, median time, and its 95% confidence interval, such as 6 and 12 months, will be evaluated at 6-month intervals.

#### 6.2.2.4 Time to Response (TTR)

Time to response (TTR) is defined as the time from the date of first dose to the first CMR and PMR (whichever is recorded first recorded) based on the PRC Response Evaluation Criteria and the first CR or PR (whichever is recorded first) assessed based on RECIST v1.1 Response Evaluation Criteria, respectively. The number, minimum, maximum, median, mean and 95% CI of TTR will be estimated.

#### 6.2.2.5 Duration of response (DOR)

Duration of response (DOR) based on PRC assessment: the time between the first achievement of CMR and PMR, whichever is recorded first, and the date of first documented increased lesion metabolism (PMD) or death due to any cause, whichever occurs first.

Duration of response (DOR) based on RECIST 1.1: the time from the first CR or PR, whichever is recorded first, to the date of first documented disease progression (PD) or death due to any cause.

The analysis population will be the objective response population with confirmed response. The censoring rules and statistical analysis methods for DOR are similar to PFS.

#### 6.2.2.6 Time to Progression (TTP)

Time to progression (TTP) is defined as the time from the first dose of study drug to disease progression. No disease progression will be considered as censored, and the TTP censoring rules and statistical methods are similar to PFS.

#### 6.2.2.7 Overall survival (OS)

Overall survival (OS): OS is defined as the time from the date of first dose of study drug to death from any cause. For patients in whom no event of death is observed, the date of last patient survival information will be obtained as the censoring date.

OS curves will be constructed using the Kaplan-Meier method to estimate median OS and its 95% CI will be estimated using the Brookmeyer and crowley method. Survival rates such as 6, 12, 24 months (i.e., the proportion of patients alive at 6, 12, 24 months after the first dose of study drug) and 95% CI will be evaluated at 6-month intervals.

#### 6.2.2.8 Subgroup Analysis

Subgroup analyses of the primary efficacy evaluation variables will be performed on the following selected baseline characteristics to explore the potential impact of these factors.

- Age (≤ 35 years/> 35 years)
- Sex (M/F)

- Tumor Classification (Multisystem Lesions/Unisystem Multifocal)
- Histiocytic tumor type (LCH/ECD/mixed/other)
- Gene mutations (BRAF mutation: BRAF V600E mutation, other mutation; MAP2K1 mutation: e102 _ I103del and L101 _ I103delinsM mutation, other MAP2R1 mutation; other mutations/unknown)
- Central Invasion (Yes/No)
- Number of prior lines of therapy (0/1-2/≥ 3)
- Prior kinase inhibitor use (Yes/No)

#### 6.2.2.9 Quality of life assessment

The QLQ-C30 (V3.0) QLQ-C30 (V3.0) of EORTC is a core scale for all cancer patients with a total of 30 items. Items 29 and 30 are divided into 7 grades, which are scored from 1 to 7 according to their answer options; other entries are graded into 4 grades: none, somewhat, comparable, and very, scored as 1 to 4.

The 30 items of the QLQ-C30 (V3.0) scale are divided into 15 domains, including 5 functional domains (physical, role, cognitive, emotional, and social functioning), 3 symptom domains (fatigue, pain, nausea and vomiting), 1 global health status/quality of life domain, and 6 single items (shortness of breath, insomnia, appetite loss, constipation, diarrhea, financial difficulties).

1) Item Score Calculation

The domain Score (RS, Raw Score) is obtained by adding and dividing the item scores included in each domain, i.e. RS = (Q1 + Q2 +... + Qn)/n. The specific scoring methods for each domain are detailed in the table below.

Scoring Method for EORTC QLQ-C30 (V3.0) Domains (Crude Score RS)

| Domain (dimension) | Properties | Number of entries | Score Full Range (R) | Scoring method |
|---|---|---|---|---|
| Physical function | Functional | 5 | 3 | (Q1+ Q2+ Q3+ Q4+ Q5)/5 |
| Role Function | Functional | 2 | 3 | (Q6+Q7)/2 |
| Emotional function | Functional | 4 | 3 | (Q21+ Q22+ Q23+ Q24)/4 |
| Cognitive function | Functional | 2 | 3 | (Q20+ Q25)/2 |
| Social functioning | Functional | 2 | 3 | (Q26+ Q27)/2 |
| General health status | | 2 | 6 | (Q29+ Q30)/2 |

| | | | | |
|---|---|---|---|---|
| Tiredness | Symptomatic | 3 | 3 | (Q10+ Q12+ Q18)/3 |
| Nausea and vomiting | Symptomatic | 2 | 3 | (Q14+ Q15)/2 |
| Pain | Symptomatic | 2 | 3 | (Q9+ Q19)/2 |
| Shortness of breath | Symptomatic | 1 | 3 | Q8 |
| Insomnia | Symptomatic | 1 | 3 | Q11 |
| Appetite loss | Symptomatic | 1 | 3 | Q13 |
| Constipation | Symptomatic | 1 | 3 | Q16 |
| Diarrhoea | Symptomatic | 1 | 3 | Q17 |
| Financial difficulties | Symptomatic | 1 | 3 | Q28 |

2) Calculation of Normalized Score

In order to make the scores of each domain comparable to each other, a linear transformation will be carried out using the polarization method to convert the crude Score into a standardized Score (SS) with values within 0 to 100. In addition, the transformation has a purpose to change the direction of the score. The QLQ-C30 scale is a reverse item (the greater the value is, the worse the quality of life) except for items 29 and 30, while it is clearly stated in the scoring rules: higher scores in the functional and general health domains indicate better functional status and quality of life, and higher scores for symptom domains indicate more symptoms or problems (the worse quality of life). Therefore, the normalized time-sharing in the domain of computational function needs to change direction. The specific calculation formula is as follows:

Functional Area: SS = [1- (RS-1)/R] * 100

Symptom Domain and Global Health Status Domain: SS = [(RS-1)/R] * 100

Where R is the full range of scores for each domain. If the domain entry has a minimum score of 1 and a maximum score of 4, then R = 3 (maximum score-minimum score).

3) Analytical Procedures

The original and standardized scores and their changes from baseline will be quantitatively described separately for each domain. Plots of standardized scores (Mean ± SD) for each domain will be plotted separately by visit.

### 6.2.3 Exploratory Endpoints

To analyze the relationship between MAPK pathway gene mutation and therapeutic effect.

The frequency of MAPK pathway gene mutations (AF%) in cfDNA will be plotted dynamically.

## 6.3 Safety Analysis

Analyses will be performed based on the Safety Analysis Set.

### 6.3.1 Study Drug Exposure and Compliance

Extent of exposure to study drug will be summarized by:

Number of cycles planned = total duration of exposure (days)/28;

The actual number of cycles is the cumulative number of dosing visit cycles prior to the end-of-treatment visit, whichever is the visit actually recorded in the CRF;

Total duration of exposure (days) = date of last dose – date of first dose + 1;

Total prescribed dose (mg) = sum of planned doses recorded in the CRF;

Actual total dose (mg) = sum of the actual administered doses recorded in the CRF;

Intensity of prescribed administration (mg/day) = total prescribed dose (mg)/total duration of exposure (days);

Actual dose intensity (mg/day) = actual total dose (mg)/total duration of exposure (days);

Relative dose intensity (%) = actual dose intensity (mg/day)/8 (mg/day) × 100%. The relative dose intensity (%) will be further classified into < 80%, 80%-120%, and > 120%, and the number and percentage of subjects will be calculated.

Compliance (%) = actual dose intensity (mg/day)/prescribed dose intensity (mg/day) × 100%. The compliance (%) will be further classified into < 80%, 80%-120%, and > 120%, and the number and percentage of subjects will be calculated.

Dose modifications will be categorized and the number and percentage of subjects will be calculated.

### 6.3.2 Adverse Events

Adverse events will be coded using the Medical Dictionary for Regulatory Activities (MedDRA) version 26.0 or later. NCI-CTCAE Version 5.0 will be used for grading evaluation.

AES will be categorized by time of onset as:

- Pretreatment-emergent adverse events: adverse events that occur before the first dose of study drug after signing the informed consent.

➢ Treatment-emergent adverse events (TEAEs): adverse events that occur from the first dose of study drug to 30 days after the last dose, and AEs/SAEs judged to be related to the investigational product after 30 days after the last dose.

An adverse event is considered a TEAE if the time relative to the first dose of study drug cannot be determined. TEAEs will be summarized and analyzed throughout the study (categorized below).

#### 6.3.2.1 Summary Table of TEAEs

A summary table of TEAEs is presented, with the number and percentage of subjects reporting at least 1 TEAE:

- All TEAEs
- Study drug-related TEAEs
- TEAEs of CTCAE Grade 3 or Higher
- Study drug-related TEAEs of CTCAE Grade 3 or higher
- CTCAE Grade 3 TEAEs
- Study drug-related CTCAE Grade 3 TEAEs
- CTCAE Grade 4 TEAEs
- Study drug-related CTCAE Grade 4 TEAEs
- TEAEs Leading to Dose Reduction
- Study drug-related TEAEs leading to dose reduction
- TEAEs Leading to Dose Interruption
- Study drug-related TEAEs leading to dose interruption
- TEAEs Leading to Discontinuation
- Study drug-related TEAEs leading to discontinuation
- Treatment-emergent SAEs
- Study drug-related SAEs

- TEAEs Leading to Death
- Study drug-related TEAEs leading to death

#### 6.3.2.2 Analysis of TEAEs by SOC and PT

The number and percentage of subjects with TEAEs will be reported by MedDRA SOC and PT for the following TEAEs.

- All TEAEs
- Study drug-related TEAEs
- TEAEs of CTCAE Grade 3 or Higher
- Study drug-related TEAEs of CTCAE Grade 3 or higher
- Treatment-emergent SAEs
- Study drug-related SAEs
- TEAEs Leading to Dose Reduction
- Study drug-related TEAEs leading to dose reduction
- TEAEs Leading to Dose Interruption
- Study drug-related TEAEs leading to dose interruption
- TEAEs Leading to Discontinuation
- Study drug-related TEAEs leading to discontinuation
- TEAEs Leading to Death
- Study drug-related TEAEs leading to death

In addition, the number and percentage of subjects with TEAEs and study drug-related TEAEs will be summarized by MedDRA SOC, PT, and severity (CT CA E Grade 1-5) for all TEAEs. If a subject experiences more than one TEAE within the same SOC or PT, the subject will be summarized only once at the corresponding SOC or PT level by the highest CT CA E grade.

TEAEs related to study medication: adverse events judged as "definitely related", "probably related",

"possibly related" and "not judged" as related to the study drug. Missing relationship determination will be classified as an adverse event possibly related to the study drug.

Subject data for all TEAEs, SAEs, study drug-related TEAEs, CT CAE ≥ Grade 3 TEAEs, TEAEs leading to death, TEAEs leading to dose interruption/study drug-related TEAEs leading to dose interruption, TEAEs leading to dose reduction/related to study drug, and TEAEs leading to discontinuation/study drug-related TEAEs leading to discontinuation will be provided in a listing form.

#### 6.3.2.3 Death

The number and percentage of subjects with death and the cause of death will be summarized and described, and the death information of all subjects who died will be presented in a listing form.

### 6.3.3 Laboratory tests

The subject's laboratory tests included hematology, blood chemistry, and coagulation tests. Quantitative laboratory variables will be summarized descriptively by visit and change from baseline, and the number of subjects, mean, standard deviation, median, quartiles (Q1 and Q3), minimum and maximum will be calculated.

Laboratory results will be graded according to NCI-CT CA E Version 5.0 and a shift table of the worst on-study laboratory values from baseline will be provided; a summary of ≥ 2 grades worsening in severity of change from baseline will be provided.

Changes in laboratory results will be graded according to NCI-CT CA E (Version 5.0) criteria, and changes from baseline to maximum toxicity grade will be summarized, and parameters without NCI-CT CA E (Version 5.0) grading will be listed only and not summarized.

The number and percentage of subjects with the most severe post-treatment changes from baseline in hematology, coagulation, and urinalysis (as judged by the investigator for clinical significance) will be qualitatively described in the form of a pre-treatment and post-treatment cross-table.

In addition, each laboratory test result (including hematology, blood chemistry, coagulation, and urinalysis) will be listed in a listing form.

### 6.3.4 Vital signs

Descriptive statistics will be performed for vital signs parameters (including systolic blood pressure, diastolic blood pressure, heart rate, respiration, and temperature) and changes from baseline at each visit, and

the number of subjects, mean, standard deviation, median, quartiles (Q1 and Q3), minimum and maximum will be calculated.

Details of vital signs are presented in a list.

### 6.3.5 Physical examination

The change from baseline in the worst post-treatment physical examination (as judged by the investigator for clinical significance) will be qualitatively described in the form of a pre-treatment and post-treatment cross-tabulation, and the number and percentage of subjects will be calculated, and all physical examinations will be listed in a listing form.

### 6.3.6 12-lead ECG

The 12-lead ECG data (QT interval, HR, RR interval, QTcF interval) of the subjects will be summarized (analyzed after the mean of the values measured at each test point), and the number, mean, standard deviation, median, quartiles (Q1 and Q3), minimum and maximum will be calculated. The number and percentage of subjects in the worst post-treatment QTcF category change from baseline will be qualitatively described in the form of a pre-treatment and post-treatment cross-table

All 12-lead ECGs will be listed in a listing format.

### 6.3.7 Other safety tests

The number and percentage of subjects will be calculated by qualitatively describing the change from baseline in the worst post-treatment ECOG performance status score using a pre-treatment and post-treatment cross-tabulation.

Quantitative ophthalmic examination variables (visual acuity test, intraocular pressure test) will be quantitatively described for subjects, and the number, mean, standard deviation, median, quartiles (Q1 and Q3), minimum and maximum will be calculated for each visit.

The number, mean, standard deviation, median, quartiles (Q1 and Q3), minimum, and maximum will be calculated for subjects ' weight at baseline, visit of study treatment, and change from baseline at the end of treatment.

Results for all ECOG performance status scores, ophthalmologic examinations, and body weight will be listed in a listing form.

## 6.4 Pharmacokinetic Analysis

All PK analyses will be performed in the PK Analysis Set. For pre-dose blood sampling points, if no drug is administered on the day before blood collection, the time point will be not included in the PK analysis; for time points after dosing, if no dose is administered on the day of PK blood collection or the day before blood collection, the time point will be not included in the PK analysis.

The plasma concentration determination data of each subject at each scheduled sampling time point will be tabulated and described. The number of subjects with valid data (n), arithmetic Mean (Mean), standard deviation (SD), coefficient of variation (CV%), geometric Mean (Geomean), Median (Median), minimum (Min) and maximum (Max) at each scheduled sampling time point will be statistically described.

The mean trough concentration (±SD) time curves of FCN-159 tablets at different visits (linear scale) and the mean plasma concentration (±SD) time curves of FCN-159 tablets on C2D1 will be plotted with the planned sampling time as the horizontal axis and the mean plasma concentration of subjects at each scheduled sampling time point as the longitudinal axis.

Plasma concentrations below the LLOQ will be expressed as "BQL". For descriptive statistics of plasma concentrations, all BQLs will be processed as 0, indicating the number of BQLs per time point. If the mean is 0, the geometric mean, coefficient of variation and geometric coefficient of variation will be processed as "NA".

Individual subject PK concentration data will be tabulated in detail.

Plasma concentration data of FCN-159 tablets obtained in this study and data from previous studies will be included in the population PK analysis. The population PK model will be described in a separate population PK analysis plan and a separate summary report will be provided and will not be included in the clinical report for this study.

## 6.5 Interim Analysis

There will be no interim analyses in this study.

# 7 AMENDMENTS TO PLANNED ANALYSES IN THE PROTOCOL

None

# 8 REFERENCES

1. ........................................................................................................... NMPA

Guidelines for Planning and Reporting of Data Management and Statistical Analysis of Drug Clinical Trials, 2016.

2. ........................................................................................................................ NMPA Guidelines for General Considerations in Drug Clinical Trials, January 2017

# 9 REVISION RECORD

| Signature Version Number | Issue Date | Reason for Change/Amendment | Supersedes |
|---|---|---|---|
| Version 1.0 | 30-Aug-2023 | Initial version | N/A |
