# Supplementary material for: Luvometinib in patients with Langerhans cell histiocytosis, Erdheim–Chester disease, and other histiocytic neoplasms: a single-arm, multicentre, phase 2 study
Source: eClinicalMedicine. 2025 Sep 17;88:103486. doi: 10.1016/j.eclinm.2025.103486 (PMC12481021; doi:10.1016/j.eclinm.2025.103486)
Supplement: Clinical Study Protocol [file mmc3.pdf]

Clinical Study Protocol

**Protocol Title:**	A multicenter, open-label, single-arm Phase 2 study to evaluate the efficacy, safety, and pharmacokinetic profile of FCN-159 in patients with histiocytic neoplasms
**Protocol Number:**	FCN-159-005
**Investigational Product:**	FCN-159
**Sponsor:**	[redacted]
**Protocol Version:**	Version 2.1
**Version Date:**	09-Mar-2023

CONFIDENTIALITY STATEMENT

All information contained in this protocol is confidential and is the property of Shanghai Fosun Pharmaceutical Industry Development Co., Ltd.

Table of Contents

Sponsor Signature Page 7
Investigator Signature Page 8
Protocol Synopsis 9
Abbreviations 17
1.Study Background 21
1.1 Medical Background 21
1.1.1 Epidemiology of LCH: 22
1.1.2 Clinical manifestations of LCH: 22
1.1.3 Epidemiology of ECD: 23
1.1.4 Clinical manifestations and diagnosis of ECD: 23
1.1.5 Treatment of LCH and ECD in Adults 23
1.2 Study Mechanism 24
1.3 Introduction of Study Drug 25
1.4 Preclinical Studies of FCN-159 26
1.4.1 Pharmacodynamic Study Results of FCN-159 26
1.4.2 Safety Study Results of FCN-159 27
1.4.3 Pharmacokinetic Study Results of FCN-159 30
1.5 Clinical Studies of FCN-159 31
1.5.1 FCN-159-001 Study 31
1.5.2 FCN-159-002 Study 32
1.5.3 FCN-159 Food Effect Study 32
1.6 Theoretical Basis of this Clinical Study 33
2 Study Objectives and Study Endpoints 34
2.1 Study Objectives 34
2.2.1 Primary Objective 34
2.2.2 Secondary Objectives 34
2.2.3 Exploratory Objectives 35
2.2 Study Endpoints 35
2.2.1 Primary Endpoints 35
2.2.2 Secondary Endpoints 35

2.2.3 Exploratory Endpoints 35
3 Study Plan 35
3.1 Overall Study Design........ 35
3.2 Tumor Assessment 37
3.3 End of Study 38
4 Selection of Study Subjects 38
4.1 Inclusion Criteria 38
4.2 Exclusion Criteria 39
4.3Treatment Discontinuation and Patient Withdrawal from the Study 41
4.3.1 Reasons for Discontinuation 41
4.3.2 Management of Patient Discontinuation 41
4.3.3 Replacement of patients who discontinued study treatment 42
4.3.4 Withdrawal of Subjects from the Study 42
5 Study Procedures 42
5.1 Screening/Baseline Period (Day -28 to -1) 42
5.2 Continuous Dose Period (CXD1 ± 3 days) 44
5.3 End of Treatment (± 7 days) 46
5.4 Safety Follow-up 46
5.5 Survival Follow-up 47
6 Study Drug and Study Methods 47
6.1 Study Drug........ 47
6.2 Packaging and Labeling of Study Drug........ 47
6.3 Provision and Storage of Study Drug 47
6.4 Drug Compliance Assessment 47
6.5 Doses and Cycles Administered 48
6.6Method of Administration 48
6.7Concomitant Medications........ 49
6.8 Permitted and Prohibited Medications and Non-pharmacotherapies 49
6.8.1 Permitted Medicinal and Non-pharmacological Therapies........ 49
6.8.2 Prohibited Medications and Non-pharmacotherapies 49
6.8.3 COVID-19 Treatment and Vaccination 50
6.9Treatment Compliance........ 51
6.10Management Principles of Adverse Reactions Occurring During the Study 51

6.10.1 Dermal toxicity....51
6.10.2Serious Hepatotoxicity....54
6.10.3 Gastrointestinal Events....54
6.10.4 Decreased Left Ventricular Ejection Fraction....55
6.10.5 Other cardiac events....56
6.10.6 Proteinuria....56
6.10.7Acute hypertension....57
6.10.8 Pneumonia/Interstitial Pneumonia (ILD)....57
6.10.9 Ocular toxicity....58
6.10.10 CK increased....58
7 Study Assessments....59
7.1Safety Assessments....59
7.1.1 Definition of Adverse Events....59
7.1.2 Collection and Recording of Adverse Events....61
7.1.3 Assessment of Adverse Events....62
7.1.4 Serious Adverse Event Reporting....64
7.1.5 Follow-up of Adverse Events....64
7.1.6 Outcome of Adverse Events....65
7.1.7 Pregnancy Reporting....65
7.1.8 Overdose....66
7.1.9 Disease progression....66
7.1.10 New Cancer....66
7.2 Efficacy Assessment....67
7.2.1PRC Criteria....67
7.2.2RECIST Criteria....68
7.2.3 Definition of Tumor Assessment Endpoints....69
7.2.4Independent Imaging Assessments....69
7.3Patient-Reported Outcomes....70
7.4 Pharmacokinetic Evaluation....70
7.4.1 Blood Sample Collection....70
7.4.2 Blood Sample Processing and Transportation....70
8 Data Management....70
8.1 Data Management System....70

8.2 Data Management Quality Control 70
8.3 Structure and Specification of Database 71
8.4Database Testing and Review 71
8.5 Data Completion Requirements 71
8.6 Query Generation and Answer 71
8.7Medical Review of Data 72
8.8 Data Locking 72
9 STATISTICAL AND STATISTICAL ANALYSIS 72
9.1 Sample Size Calculation 72
9.2Analysis Set 73
9.3Safety Analyses 73
9.4 Efficacy Analysis 73
9.5 Pharmacokinetic Analyses 74
9.6 Analysis of Clinical Outcomes 74
9.7 Interim Analyses 74
10 Study Management 74
10.1 Ethical Considerations 74
10.2 Informed Consent 75
10.3 Protocol Amendments 76
10.4 Protocol deviations 76
10.5Patient Confidentiality and Privacy 77
10.6 Monitoring 77
10.7 Quality Assurance and Quality Control 78
10.8 Direct Access 78
10.9 Data Recording and Storage 79
10.10 Insurance and Patient Compensation 79
10.11 Storage and Use of Biological Specimens 79
10.12Study Interruption and Early Termination 79
10.13 Study Summary Report 80
10.14 Information Disclosure and Data Publishing Policies 80
10.15 Conflict of Interest Statements 80
11 References 81
Attachment 1 Pharmacokinetic Sample Collection Schedule 84

Attachment 2 Study Flow Chart 85
Attachment 3 Quality of Life Inventory for Oncology Patients EORTC QLQ-C30 (V3) 89
Annex 4 ECOG Performance Status 91
Annex 5 New York Heart Association functional class (NYHA) 92
Attachment 6 COCKCROFT-GAULT Formula 93
Appendix 7 Response Evaluation Criteria in Solid Tumors (RECIST version 1.1) 94
Attachment 8 Drugs that May Prolong QTc 109
Attachment 9 COVID-19 110

Sponsor Signature Page

Protocol Title:	A multicenter, open-label, single-arm Phase 2 study to evaluate the efficacy, safety, and pharmacokinetic of FCN-159 in patients with histiocytic neoplasms
Protocol Number:	FCN-159-005
Version Date and Version Number:	March 09, 2023, Version 2.1
Sponsor Name:	[redacted]
Sponsor Address:	[redacted]
Approver Name:	Zhuli Wu
Title:	[redacted]
Approver Signature: _	Signature Date: _

Investigator Signature Page

Protocol Title:	A multicenter, open-label, single-arm Phase 2 study to evaluate the efficacy, safety, and pharmacokinetic of FCN-159 in patients with histiocytic neoplasms
Protocol Number:	FCN-159-005
Version Date and Version Number:	March 09, 2023, Version 2.1

I have read this protocol and agree to conduct the clinical study in accordance with all the provisions of the protocol, current regulations and the ethical principles in the Declaration of Helsinki.

Principal Investigator Name:

Signature of Principal Investigator: _	Date: _

Protocol Synopsis

**Protocol Number:**	FCN-159-005
**Protocol Title:**	A multicenter, open-label, single-arm Phase 2 study to evaluate the efficacy, safety, and pharmacokinetic of FCN-159 in patients with histiocytic neoplasms
**Study drug:**	FCN-159
**Clinical Study Phase:**	Phase 2
**Number of patients:**	Approximately 28 patients in total
**Study Period:**	• Screening period: day -28-Day -1;<br>• Dosing period: 28 consecutive days in a 28-day cycle until disease progression, death, intolerable toxicity, patient withdrawal of consent, or end of study;<br>• Their safety and survival status will be followed up after the last treatment.<br>The end of study is defined as 2 years after the last patient ’ s first dose of FCN-159 or the last patient ’ s last dose of FCN-159, whichever occurs earlier.
**Study Objectives:**	**Primary Objective:**<br>• To evaluate the efficacy of FCN-159 in patients with Langerhans cell histiocytosis (LCH), Erdheim-Chester disease (ECD), and other histiocytic tumors.<br>**Secondary objectives:**<br>• To evaluate the safety of FCN-159 in patients with LCH, ECD, and other histiocytic tumors.<br>• To further evaluate the efficacy of FCN-159 in patients with LCH, ECD, and other histiocytic tumors in addition to ORR assessed by IRC based on PET Response Criteria (PRC).<br>• The PK profile of FCN-159 will be further evaluated.<br>• To evaluate the improvement of FCN-159 on quality of life in patients with LCH, ECD, and other histiocytic tumors compared with baseline examinations.<br>**Exploratory Objective:**<br>• To analyze the relationship between MAPK pathway related gene mutation and therapeutic effect.
**Study Endpoints:**	**Primary endpoint:**<br>• Objective Response rate (ORR) assessed by the Independent Review Committee (IRC) based on PET response Criteria (PRC).<br>**Secondary endpoints:**<br>• ORR assessed by the investigator based on PET Response Assessment Criteria (PRC); ORR, Disease control rate (DCR), Clinical benefit rate (CBR), Time to Response (TTR), progression-free survival (PFS), and Overall survival

	(OS) assessed by IRC and investigators based on RECIST v1.1.<br>• Safety endpoints: the number and severity of adverse events, serious adverse events, deaths, and safety test abnormalities (e.g., laboratory tests, vital signs, physical examinations, electrocardiograms, ECOG, etc.) are evaluated according to the National cancer institute common terminology criteria for adverse events (NCI-CTCAE) version 5.0; proportion of patients with dose modification or discontinuation due to drug toxicity.<br>• Changes in the Quality of Life (QOL) EORTC-QLQ-C30 (Version 3.0) scale.<br>• Population PK parameters of FCN-159.<br>**Exploratory endpoints:**<br>• To evaluate MAPK pathway gene mutations in patients and to analyze the correlation with efficacy.
**Study Design:**	**Study Design:**<br>This is a single-arm, open-label, multicenter Phase 2 clinical study to evaluate the efficacy, safety, and PK profile of FCN-159 as a single agent in patients with Langerhans cell histiocytosis (LCH), Erdheim-Chester disease (ECD), and other histiocytic tumors.<br>It is recommended that Patients provide a sufficient number of histopathological slides or tumor tissue samples and peripheral blood for central laboratory testing of the following biomarkers: including but not limited to ERBB3, RAF-1, BRAF, ARAF, HRAS, KRAS, NRAS, MEK (MAP2K1 and MAP2K2), and other MEK upstream genes.<br>A total of 28 patients with LCH, ECD, and other histiocytic tumors will be included in this study, and the diagnosis will be reviewed by the central pathology of the leading unit. FCN-159 will be administered at a dose of 8 mg/day, orally as a single agent, once daily, continuously every 28 days as a treatment cycle, until disease progression, death, intolerable toxicity, and withdrawal of informed consent.<br>Tumor Response will be evaluated once at screening, at the end of 3rd, 6th, 12th treatment cycles (± 7 days), and at the end of treatment by IRC and investigator according to PET Response Criteria (PRC). Tumor assessments will be performed by the IRC and the investigator at screening, at the end of the 3rd, 6th, 9th, 12th treatment cycles (± 7 days), and at the end of treatment according to RECIST 1.1 criteria. If the imaging results are considered to be consistently stable by the Principal Investigator after 12 treatment cycles, tumor assessments will be performed every 4 treatment cycles (± 14 days) according to RECIST 1.1, if the imaging results do not reach sustained stability in the opinion of the investigator after 12 treatment cycles, tumor assessments will be performed every 3-6 treatment cycles (± 14 days) according to PRC criteria, and tumor assessments will be performed every 3 treatment cycles (± 7 days) according to RECIST 1.1. FCN-159 treatment lasted up

	to 2 years. The quality of life and the detection of MAPK pathway gene mutations in plasma free DNA (cfDNA) will be assessed at the same time as those assessed by RECIST 1.1 criteria. The study will also evaluate patient safety, mainly including monitoring and recording of AEs and SAEs, performing protocol-specified laboratory tests (including hematology, blood biochemistry, urinalysis, etc.), 12-lead ECG, cardiac ultrasound or MUGA, ophthalmology, vital signs, physical examination, etc. The follow-up period includes safety follow-up and survival follow-up.<br>This study will also further evaluate the PK profile of FCN-159 in patients with histiocytic tumors.<br>**Dose Modification:**<br>Dose modifications are allowed based on toxicities and side effects. When toxicities return to Grade 0-1, the patient may maintain the same dose level or reduce to the next lower dose level as assessed by the investigator (at this time, the dose has been explored at the time of dose escalation, 8 mg, 6 mg, and 4 mg, respectively). The lowest dose is not less than 4 mg. If discontinuation for more than 28 days due to an AE, study treatment needs to be permanently discontinued.<br>**Tumor assessment:**<br>Histiocytic tumor lesions will be evaluated by whole-body 18FDG-PET/CT scan or MRI/CT scan according to PET Response Assessment Criteria (PRC) and RECIST v1.1, respectively. Tumor Response will be evaluated once at screening, at the end of 3rd, 6th, 12th treatment cycles (± 7 days), and at the end of treatment by IRC and investigator according to PET Response Criteria (PRC). Patients evaluated as CMR or PMR had confirmed response by repeated assessments at least 4 weeks later. Tumor assessments will be performed by the IRC and the investigator at screening, at the end of the 3rd, 6th, 9th, 12th treatment cycles (± 7 days), and at the end of treatment according to RECIST 1.1 criteria. If the imaging results are considered to be consistently stable by the Principal Investigator after 12 treatment cycles, tumor assessments will be performed every 4 treatment cycles (± 14 days) according to RECIST 1.1, if the imaging results do not reach sustained stability in the opinion of the investigator after 12 treatment cycles, tumor assessments will be performed every 3-6 treatment cycles (± 14 days) according to PRC criteria, and tumor assessments will be performed every 3 treatment cycles (± 7 days) according to RECIST 1.1. FCN-159 treatment lasted up to 2 years. If a patient discontinues treatment, tumor assessments should continue as scheduled until disease progression, death, withdrawal of informed consent, loss to follow-up, start of new anti-tumor therapy, or end of the study.<br>**End of study:**<br>The end of study is defined as 2 years after the last patient ’ s first dose of FCN-159 or the last patient ’ s last dose of FCN-159, whichever occurs earlier.
**Study subjects:**	Patients with LCH, ECD, and other histiocytic neoplasms.

**Inclusion criteria:**	The following conditions must be met for patient enrollment:<br>1. Age > 16 years.<br>2. Patients with histologically confirmed LCH, ECD, or other histiocytic tumors.<br>3. A sufficient number of histopathological slides or tumor tissue samples and peripheral blood will be available for central laboratory testing of the following biomarkers: including but not limited to ERBB3, RAF-1, BRAF, ARAF, HRAS, KRAS, NRAS, MEK (MAP2K1 and MAP2K2), and other MEK upstream genes.<br>4. Patients with LCH require multisystem involvement (greater than one system) or single-system multifocal disease (greater than one lesion).<br>5. Presence of evaluable lesions based on PET response criteria (PRC).<br>6. Patients with treatment-naïve or relapsed, refractory LCH, ECD, or other histiocytic tumors may be included.<br>7. Expected survival is at least 3 months.<br>8. ECOG performance score 0-2, see Attachment 4.<br>9. Ability to understand and voluntarily sign written informed consent.<br>10. Premenopausal women had a negative serum human chorionic gonadotropin (HCG) pregnancy test within 7 days prior to initiation of dosing, and a serum human chorionic gonadotropin (HCG) pregnancy test may not be performed in surgically infertile or non-fertile women ≥ 1 year postmenopausal.<br>11. For patients of childbearing potential: patients should agree to use effective contraception, double-barrier contraception, condoms, oral or injectable contraceptives, intrauterine devices, etc. during treatment and for at least 90 days after the last dose of study treatment. Male patients should agree to refrain from donating sperm for at least 90 days after the last dose.<br>12. Adequate bone marrow function: absolute neutrophil count ≥ 1.5 × 109/L, hemoglobin ≥ 90 g/L, and platelets ≥ 100 × 109/L in the absence of blood transfusion, blood products, and granulocyte colony-stimulating factor. Patients with cytopenia due to the disease itself below these thresholds may be waived from enrollment based on the investigator ' s comprehensive judgment.<br>13. Adequate liver and kidney function: Aspartate aminotransferase (AST), Alanine aminotransferase (ALT) ≤ 2.5 × Upper limit normal (ULN), and ≤ 5 × ULN if liver invasion occurs; serum total bilirubin ≤ 1.5 × ULN, ≤ 5 × ULN in patients with Gilbert ' s syndrome or liver involvement; alkaline phosphatase (AKP) ≤ 2.5 × ULN, or ≤ 10 × ULN if liver invasion occurs; serum creatinine ≤ 1.5 × ULN, and if serum creatinine > 1.5 × ULN, creatinine clearance (CCr) ≥ 50 mL/min (see Attachment 6 based on Cockcroft-Gault formula); albumin ≥ 3 g/dL.<br>14. Coagulation function: international normalized ratio (INR) and activated partial thromboplastin time (APTT) ≤ 1.5 ULN.
**Exclusion criteria:**	Patients who meet any of the following conditions cannot be

	enrolled in this clinical study:<br>1.Patients with one of the following prior therapies:<br>a. Chemotherapy, targeted therapy, immunotherapy, biotherapy, investigational therapy, or herbal anti-tumor therapy for histiocytic tumors within 4 weeks or < 5 half-lives (whichever is longer) before starting study drug.<br>b. Received strong CYP3A4, CYP2C8, CYP2C9 inhibitors or inducers within 14 days prior to starting study drug, except for topical use in the skin.<br>c. Use of growth factors that promote platelet or leukocyte numbers or function within 7 days prior to starting study drug.<br>d. Radiation therapy and surgery within 4 weeks prior to starting study drug.<br>e. Patients who have participated in other interventional clinical trials within 4 weeks prior to starting study drug.<br>f. Prior treatment with MEK 1/2 inhibitors.<br>g. High-dose chemotherapy and stem cell transplant salvage (autologous stem cell transplant) or allogeneic stem cell transplant within 90 days prior to enrollment. Patients receiving post transplant anti-Graft Versus Host Disease (GVHD) drugs such as cyclosporine, tacrolimus, or other drugs to prevent GVHD after BMT cannot participate in this trial.<br>h. For patients with brain tumors (intracranial masses), anticoagulants were used within 7 days prior to starting study drug.<br>i. Prednisone treatment < 0.5 mg/kg/day (or equivalent dose of other corticosteroids) within one month prior to enrollment is allowed, but must be stopped 14 days before starting study drug. Patients with brain lesions treated with corticosteroids for brain edema must remain on a stable dose for 14 days prior to enrollment. Hormone replacement therapy is permitted in patients with hypopituitarism due to primary pituitary involvement.<br>2.The patient had a history or concurrent malignancy of other malignancies (excluding cured non-melanoma basal cell carcinoma of the skin, carcinoma in situ of the breast or carcinoma in situ of the cervix, and other malignancies without evidence of disease within 5 years).<br>3.Uncontrolled stable hypertension (with medical therapy): systolic blood pressure > 160 mm Hg and/or diastolic blood pressure > 100 mm Hg.<br>4.The patient had dysphagia, active digestive disease, malabsorption syndrome, or other conditions that affected the absorption of study drug.<br>5.Prior or current retinal vein occlusion (RVO), retinal pigment epithelial detachment (RPED), glaucoma, and other significant abnormalities.<br>6.Interstitial pneumonia, including clinically significant radiation

	pneumonitis. Interstitial pneumonia due to primary pulmonary involvement is excluded.<br>7.Cardiac function or comorbidities that meet one of the following conditions will be excluded:<br>a. Three 12-lead Electrocardiogram (ECG) measurements were performed at the study site during the screening period, and the mean of the three times is calculated according to the QTcF formula of the instrument, and the QTc F is > 470 milliseconds; for patients with risk factors for QTcF prolongation, such as inability to correct hypokalemia, hereditary long QT syndrome; or receive drugs that prolong the QTcF interval (mainly class Ia, Ic, III antiarrhythmic drugs). Drugs with the potential to prolong the QTcF interval are listed in Attachment 8.<br>b. New York Heart Association (NYHA) grade ≥ 3 congestive Heart failure, see Annex 5;<br>c. Clinically significant arrhythmias, including, but not limited to, complete left bundle branch conduction abnormalities, second-degree atrioventricular block;<br>d. Known concurrent clinically significant coronary heart disease, cardiomyopathy, severe valvular disease.<br>e. Echocardiography revealed a left ventricular ejection fraction (LVEF) < 50%.<br>8.With active bacterial, fungal, or viral infection, including active hepatitis B (hepatitis B virus surface antigen positive and hepatitis B virus DNA more than 1000 IU/ml or meeting site criteria for diagnosis of active hepatitis B infection) or hepatitis C (hepatitis C virus RNA positive), human immunodeficiency virus infection (HIV positive).<br>9.Pregnant or lactating women.<br>10.Known hypersensitivity to study drug, other MEK1/2 inhibitors, or excipients thereof.<br>11.Clinically significant circumstances that, in the opinion of the investigator, would preclude participation in the study or inability to comply with safety requirements.
**Study Drug and Dosage and Administration:**	FCN-159 tablets: strengths: 1.0 mg/tablet and 4.0 mg/tablet.<br>The drug will be given as a single agent orally once daily at 8 mg/day for a 28-day treatment cycle.
**Safety evaluation:**	It mainly includes monitoring and recording AEs and SAEs, performing protocol-specified laboratory tests (including hematology, blood biochemistry, urinalysis, etc.), 12-lead ECG, cardiac ultrasound or MUGA, ophthalmic examination, vital signs examination, physical examination, etc. AEs will be graded according to NCI-CT CA E Version 5.0 criteria.
**Efficacy evaluation:**	Tumor Response will be evaluated once at screening, at the end of 3rd, 6th, 12th treatment cycles (± 7 days), and at the end of treatment by IRC and investigator according to PET Response Criteria (PRC). Patients evaluated as CMR or PMR had confirmed

	response by repeated assessments at least 4 weeks later. Tumor assessments will be performed by the IRC and the investigator at screening, at the end of the 3rd, 6th, 9th, 12th treatment cycles (± 7 days), and at the end of treatment according to RECIST 1.1 criteria. If the imaging results are considered to be consistently stable by the Principal Investigator after 12 treatment cycles, tumor assessments will be performed every 4 treatment cycles (± 14 days) according to RECIST 1.1, if the imaging results do not reach sustained stability in the opinion of the investigator after 12 treatment cycles, tumor assessments will be performed every 3-6 treatment cycles (± 14 days) according to PRC criteria, and tumor assessments will be performed every 3 treatment cycles (± 7 days) according to RECIST 1.1. FCN-159 treatment lasted up to 2 years. Patients who discontinue treatment due to toxicity should continue to undergo disease status assessments per schedule until disease progression or other criteria listed above are met.
**Quality of life assessment:**	Quality of life assessments will be performed in parallel with RECIST 1.1 assessments, once at screening, at the end of treatment cycles 3, 6, 9, 12 (± 7 days), and at the end of treatment. If the imaging results are considered to be consistently stable by the Principal Investigator after 12 treatment cycles, every 4 treatment cycles (± 14 days), and every 3 treatment cycles (± 7 days) if, in the opinion of the investigator, the imaging results do not reach sustained stability after 12 treatment cycles.
**Pharmacokinetic evaluation:**	To evaluate the PK profile of FCN-159, plasma samples were collected at the following time points, the concentrations of FCN-159 in plasma were measured, and the corresponding population pharmacokinetic parameters were calculated.<br>C1D1: pre-dose<br>C2D1: pre-dose; 0.5-1 hour, 1.5-3 hours, 4-6 hours after dosing;<br>C4D1, C7D1, C10D1: pre-dose.<br>(Blood sampling time points may be adjusted based on available PK data)
**Statistical Analysis:**	
**Sample Size:**	This study is a single-arm clinical study of rare diseases, assuming that the proportion of patients achieving CMR and PMR is equal to 80% (95% CI 59.3%, 93.2% when 25 evaluable patients are enrolled), considering a dropout rate of 10%, approximately 28 patients are planned to be enrolled in the study.
**Analysis Population:**	• Intent-to-Treat (ITT): patients who signed informed consent and took at least one dose of FCN-159.<br>• Modified Intent-to-Treat (mITT): patients who took at least one dose of FCN-159, had baseline tumor assessment data and at least one post-baseline tumor assessment data, and had no major protocol violations.<br>• Safety Analysis Set: any patient who took at least one dose of FCN-159 and had at least 1 safety assessment.<br>• PK Analysis Set: includes patients who took at least one dose of

	FCN-159 and had at least 1 PK blood sample collected as planned and had plasma concentration data of study drug.
**Safety analysis:**	Safety will be evaluated by summaries of AEs, laboratory tests (including hematology, blood chemistry, urinalysis, etc.), 12-lead ECG, ophthalmology, vital signs, physical examination, ECOG, etc. AEs will be summarized separately during the first cycle of the continuous monotherapy period and throughout the treatment period, and treatment-related AEs, SAEs, AEs with toxicity grade ≥ 3, and AEs leading to discontinuation will be summarized.<br>A TEAE is defined as an adverse event that occurs from the first dose of study drug to 30 days after the last dose, and an AE/SAE judged to be related to the investigational drug after 30 days after the last dose. Adverse events will be coded using MedDRA and graded using NCI-CT CA E version 5.0.<br>Laboratory changes will be summarized. For laboratory variables, maximum toxicity occurring during the study will be summarized by count and percentage. Descriptive statistics will be performed for changes from baseline in vital signs, physical examination, 12-lead electrocardiogram, and ECOG score.
**Efficacy analysis:**	To assess treatment response, the number and percentage of subjects with ORR (defined as CMR + PMR/CR + PR) will be calculated, and the 95% confidence interval of ORR will be calculated using the Clopper-Pearson method.<br>For dichotomous endpoints such as DCR, CBR, descriptive analyses will be performed and 95% confidence intervals will be calculated using the Clopper-Pearson method. For endpoints such as PFS, DOR, TTR, and OS, the Kaplan-Meier method will be used to estimate survival curves with median time and 95% confidence intervals.
**Quality of life analysis:**	Patient-reported changes in quality of life were analyzed. There are no established valid boundaries for the clinical significance of changes in quality of life for patients with histiocytic tumors. The study will summarize the effect of treatment on quality of life over time.
**Pharmacokinetic analysis:**	FCN-159 plasma concentration data obtained in this study and data from previous studies will be included in the population PK analysis. The population PK model will be described in a separate population PK analysis plan and a separate summary report will be provided and will not be included in the clinical report for this study.
**Biomarker Analysis:**	Biomarker measurements, including MEK and other upstream gene mutation burden on the MAPK pathway in plasma cfDNA These measurements are taken periodically before and during treatment. The evolution of these measurements and their correlation with the treatment effect will be illustrated graphically.
**Protocol Date:**	09-Mar-2023

Abbreviations

Abbreviations	Chinese translation
AE	Adverse Events
AKP	Alkaline phosphatase
ALT	Alanine aminotransferase
APTT	Activated partial thromboplastin time
AST	Aspartate aminotransferase
AR	Accumulation factor
AUC	Area under the concentration-time curve
AUC(0-last)	Area under the concentration-time curve from time point 0 (pre-dose) to the last measurable time of concentration
AUC(0-∞)	Area under the concentration-time curve from time point 0 (predose) extrapolated to time infinity
AUC(0-tau)	Area under the plasma concentration-time curve over a dosing interval after reaching steady state
BSA	Body surface area
DOR	Duration of response
cNF	Cutaneous neurofibroma
NMPA	National Medical Products Administration
CBR	Clinical Benefit Rate
CCr	Creatinine clearance
cfDNA	Plasma free DNA
CI	Confidence interval

Abbreviations	Chinese translation
Cmax	Maximum plasma concentration
Cmin	Blood trough concentration
CMR	Complete metabolic response
CNS	Axial nervous system
Css, min	Trough concentration at steady state
Css, avg	Mean plasma concentration at steady state
COA	Clinical Outcome Assessment
CPK	Creatine phosphokinase
CK-MB	Creatine phosphokinase MB
CR	Complete response
CRO	Contract Research Organization
CT	Computed tomography
CL	Creatinine clearance
CL/F	Drug clearance
CLss/F	Steady-state drug clearance
CYP	Cytochrome P450 enzymes
DCR	Disease control rate
DOR	Duration of response
DLT	Dose Limiting Toxicity
EC	Ethics Committee
ECG	Electrocardiogram
ECOG	Eastern Cooperative Oncology Group
EDC	Electronic Data Acquisition
ERK	Extracellular signal-regulated kinase
ECD	Erdheim-Chester disease
eCRF	Electronic Case Report Form
FDA	Food and Drug Administration
FDG	Deoxyglucose
GCP	Good Clinical Practice
GGT	Glutamyl transpeptide
GVHD	Received anti-graft versus host disease
HBcAb	Hepatitis B virus core antibody
HBeAb	Hepatitis B virus e antibody
HBeAg	Hepatitis B virus e antigen
HBsAb	Hepatitis B virus surface antibody
HBsAg	Hepatitis B virus surface antigen
HBV	Hepatitis B virus
HCV	Hepatitis C virus
HCG	Serum human chorionic gonadotropin
HIV	Human immunodeficiency virus
HLH	Hemophagocytic lymphohistiocytosis
HNSTD	Maximum No-Serious Adverse Effect Level
HS	Histiocytic sarcoma
IC50	Half maximal inhibitory concentration
ICF	Informed Consent Form
ICH	Indeterminate cell histiocytosis
IEC	Independent Ethics Committee

Abbreviations	Chinese translation
Ig	Administration by gavage
INR	International Standardized Ratio
IRB	Institutional Review Board
IRC	Independent Review Committee
IRT	Interactive Response Technology System
ITT	Intent-to-treat Analysis Set
JXG	Juvenile xanthogranulomatous disease
LC	Langerhans cells
LCH	Langerhans cell histiocytosis
LSGM	Least Squares Geometric Mean
LVEF	Left ventricular ejection fraction
LYM	Percentage of lymphocytes
MAPK	Mitogen-activated protein kinase
MEK	Mitogen-activated protein kinase
MTD	Maximum tolerated dose
MONO	Monocyte count
MPNST	Malignant peripheral nerve sheath tumor
MPS	Mononuclear phagocyte system
MRI	Magnetic resonance imaging
mITT	Modified Intent-to-Treat Analysis Set
MUGA	Multigated angiograms
NCI-CTCAE	National Common Toxicity Criteria for Adverse Events in Cancer Research
NEU	Neutrophils
NOAEL	No toxic effect level
NRAS	Neuroblastoma murine sarcoma oncogene
NF1	Neurofibromatosis type 1
NYHA	New York Heart Association
ORR	Objective response rate
OS	Overall survival
PD	Progressive disease
PRC	PET Response Assessment Criteria
PET-CT	Positron emission computed tomography
PFS	Progression-free survival
PK	Pharmacokinetics
PKCS	Pharmacokinetic concentration set
PMD	Increased metabolism of lesions
PMR	Partial metabolic response
PN	Plexiform neurofibroma
PT	Prothrombin time
PR	Partial response
PRO	Patient-reported outcomes
QD	Once a day
QOL	Quality of life

**Abbreviations**	**Chinese translation**
RAS	Murine sarcoma oncogene
RDD	Rosai-Dorfman disease
RET	Reticulocytes
RNA	Ribonucleic acid
ROI	Region of Interest of Lesions
RP2D	Recommended Phase 2 Dose
RPED	Detachment of retinal pigment epithelium
RVO	Retinal vein occlusion
SAE	Serious Adverse Events
SAR	Structure-activity relationship
SAS	Safety Analysis Set
SD	Stable disease
SMD	Stable metabolic response
SS	Safety Analysis Set
SULpeak	Peak Standard Uptake Values Based on Lean Body Weight
SUSAR	Suspected unexpected serious adverse reaction
SUV	Standard Uptake Value
SUVmax-BW	Maximum UVS observed within the region of interest of each target lesion after normalization to body weight
Tmax	Time to maximum plasma concentration
t1/2	Half-life
TEAE	Treatment-emergent AEs
TTP	Time to progression
TTR	Time to response
ULN	Upper limit of normal
Vd/f	Apparent volume of distribution of drug
WBC	White blood cell count

1.Study Background

1.1 Medical Background

Histiocytosis is a group of diseases defined by the pathological infiltration of normal tissues by Mononuclear phagocyte system (MPS) cells. Because of the biological variability of MPS cells and their tissues, histiocytic tumors are one of the most complex fields in modern hematology, and diagnosis is very difficult. Histiocytic neoplasms are a heterogeneous group of clonal hematopoietic diseases marked by different mutations in genes in the Mitogen-activated protein kinase (MAPK) pathway.

According to the fourth edition of the World Health Organization classification, histiocytic diseases can be divided into two broad categories according to the phenotype of the cells within the lesion: 1) Langerhans cell histiocytosis (LCH) and 2) non-Langerhans cell histiocytosis (non-LCH).

In the WHO classification system, Langerhans cell (LC) lesions are divided into two subgroups according to the degree of cytological atypia and clinical aggressiveness: LCH and Langerhans cell sarcoma. In contrast, non-LCH is a heterogeneous group of diseases, including Erdheim-Chester disease (ECD), Juvenile xanthogranulomatous disease (JXG), Rosai-Dorfman disease (RDD), Histiocytic sarcoma (HS), Indeterminate cell histiocytosis (Indeterminate cell histiocytosis,ICH) and other diseases defined by histiocytic aggregation that do not meet the diagnostic criteria for LCH, LC sarcoma, or hemophagocytic lymphohistiocytosis (HLH) [1].

In addition to the WHO classification, the Histiocytic Society has in recent years proposed a new classification of histiocytosis that combines clinical pathology, prognosis, and new genetic findings that are not taken into account in the WHO classification. This new classification system divides histiocytic tumors into 5 groups: "L"(Langerhans), "C"(skin and mucocutaneous), "M"(malignant), "R"(Rosai-Dorfman), and "H"(hemophagocytic) groups. An important motivation for regrouping histiocytic tumors is the inclusion of a large number of new molecular genetic information that reveals genetic similarities between LCH and non-LCH tumors. Thus, in the revised Histiocytic Society classification, Group L includes LCH and ECD, which share MAPK pathway gene mutations in more than 80% of cases and may coexist [1-2].

Langerhans cell histiocytosis (LCH) and Erdheim-Chester disease (ECD) are rare diseases, and are included in the first catalogue of rare diseases published in China in June 2018, and the incidence is very low. LCH and ECD in adults are both histiocytoproliferative tumors.

Abnormally proliferating histiocytes affect all organs of the body, and the more common sites of involvement include bone, skin, lung and pituitary gland, so the heterogeneity of clinical symptoms is very strong, ranging from indolent disease of single lesions to invasive disease of life-threatening systemic and multisystem involvement, with a proportion of 82%-92.8% [3-5]. Patients with multisystem involvement often have multiple clinical symptoms, such as bone pain, diabetes insipidus, dyspnea, and require active systemic treatment.

1.1.1 Epidemiology of LCH:

The reported incidence of LCH is 2.6 to 8.9 per million children under 15 years of age per year, with a median age at diagnosis of 3 years [7]. The exact incidence of LCH in adults is unclear, and a study based on the SEER database showed an incidence of 0.07 parts per million per year (95% CI 0.05-0.1) [6].

1.1.2 Clinical manifestations of LCH:

LCH is an inflammatory myeloid tumor caused by clonal expansion of myeloid precursor cells that differentiate into CD1a +/CD207 + cells in lesions, resulting in a range of organ involvement and dysfunction. LCH patients of all ages have varying degrees of systemic involvement, and severe long-term neurological or endocrine complications may affect quality of life [7]. LCH is characterized by abnormalities in cell function, differentiation, or proliferation of the mononuclear phagocyte system. In LCH, granulomatous lesions consisting of langerin-positive (CD207 +) histiocytes and inflammatory infiltrates may occur in almost any organ system, but changes are prone to bone, skin, lung, and pituitary. LCH is characterized by a continuous spectrum of diseases ranging from solitary lesions to extensive disseminated disease with organ dysfunction [8]. The current classification system is based on the site of the lesion, the number of sites involved (single- or multisystem/local or multifocal), and whether the disease involves organs at risk (hematopoietic system, liver, or spleen).

Bone is the most commonly affected system because bone lesions are present in approximately 80% of patients with LCH, half of whom have a single lesion [9]. The most common sites of bone involvement are the skull, followed by the spine, extremities, and pelvis [10].

Skin involvement is also common, particularly in infants, manifesting as seborrheic eczema. In adults, refractory eczema may manifest in the intertendinous and genital areas [7].

In children, lung involvement usually occurs in the context of multisystem disorders, with lung involvement reported in up to 35% of patients [11]. Imaging findings were typical of reticulonodular changes with alveolar formation. In the absence of other risk organ

involvement, lung disease is not a predictor of poor prognosis [12]. Solitary pulmonary involvement is a rare manifestation that occurs almost exclusively in adults with smoking habits [7].

Hematopoietic dysfunction in the form of cytopenia is a poor prognostic marker. It occurs in cases of multisystem involvement, usually in very young children. Its pathophysiology is multifactorial, including direct involvement of the bone marrow and destruction of blood cells due to hypersplenism due to LC infiltration of the spleen [7].

Prognosis of liver involvement is also poor. Patients presented with hypoalbuminemia, hepatomegaly, or hyperbilirubinemia. In children and adults, sclerosing cholangitis and hepatic fibrosis are rare complications that usually progress to end-stage liver failure [7].

Patients with LCH reactivation or chronic disease may experience serious permanent consequences that decrease the quality of life of patients, particularly when they affect the central nervous system or lungs, leading to hormone deficiency, neurodegenerative syndromes, pulmonary fibrosis, etc.

1.1.3 Epidemiology of ECD:

The incidence of ECD is very low, with a total of approximately 1500 cases reported worldwide since the disease was first reported in 1930 [3].

1.1.4 Clinical manifestations and diagnosis of ECD:

Erdheim-Chester disease (ECD) is a very rare non-Langerhans cell histiocytosis with unknown etiology and pathogenesis. It has been reported that the disease occurs predominantly in adult males over 40 years of age, but it can also occur in females and is uncommon in children. Clinical manifestations of ECD range from asymptomatic to multisystem involvement. ECD typically affects bones, kidneys, retroperitoneal spaces, skin, and brain. Without treatment, disease progression is rapid, with fatal consequences due to severe lung disease, chronic renal failure, cardiomyopathy, and other complications.

Diagnosis of ECD relies on imaging and biopsy pathology of affected organs, i.e. fibrosis and infiltration of foamy histiocytes, lymphocytes, and plasma cells in tissues. Immunohistochemistry showed that CD68 and CD163 were positive, CD1a was negative, and S-100 positive rate was 20%.

1.1.5 Treatment of LCH and ECD in Adults

At present, there is no standard treatment regimen for adult patients with LCH, and clinically, cytotoxic drugs are selected for chemotherapy, such as cytarabine, vinbine + prednisone, cladribine, methotrexate + cytarabine, etc. [4-5, 13-15]. There is no standard

treatment regimen for patients with ECD, and commonly used chemotherapeutic drugs are: interferon-α, cladribine, etc. The efficacy of different chemotherapy regimens varied, with ORR values ranging from 54% to 87.9%, with an average of about 73%. Although the ORR value of methotrexate + cytarabine chemotherapy regimen in adult patients with LCH was up to 87.9%, it had high toxic and side effects, with the proportion of Grade 3 and 4 adverse reactions up to 94%, of which the incidence of Grade 4 adverse reactions was 42% [2]. When concurrent chemotherapy regimens were used, a subset of patients failed to respond to chemotherapeutic agents and disease could not be controlled, resulting in disease progression, occurring in 6% -41.6% of patients.

1.2 Study Mechanism

Extracellular signal-regulated protein kinase (ERK), RAS-RAF-Mitogen-activated protein kinase (MEK), is a widely activated Mitogen-activated protein kinase (MAPK) pathway, which can transmit Extracellular signals into the nucleus and cause changes in the expression profile of specific proteins, thus affecting cell fate.These include proliferation, differentiation, apoptosis and metabolism. In many tumors, protein members of this pathway are overexpressed or mutated. In this signaling pathway, only ERK1 and ERK2 are known substrates of MEK proteins, and activated MEK can phosphorylate the major downstream target ERK. ERK is a critical regulator of cell growth and proliferation. Once the pathway is abnormally and continuously activated, uncontrolled cell proliferation and apoptosis are blocked, leading to tumorigenesis. The mechanism of the study is detailed in Figure 1.

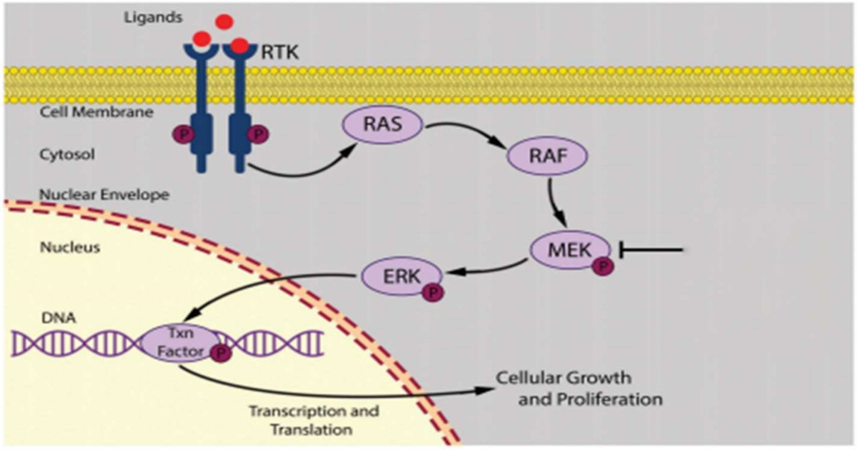

Figure 1 Study Mechanism Chart

Somatic mutations in mitogen-activated protein kinase (MAPK) signaling pathway genes and LCH:

Recent studies have shown that LCH and ECD are characterized by a significant dependence on MAPK signaling. Badalian-Very et al found the BRAF V600E mutation in 57% of LCH lesions [16]. The mutation frequency of BRAF V600E in ECD is 50% -70% [17]. Professor Li Jian ' s team from Peking Union Medical College Hospital summarized the data of 155 adult patients with histiocytic tumors who visited the center from January 2005 to December 2020 and found that the most common kinase mutations in adult histiocytic tumors included BRAF V600E (52/155, 33.5%), other BRAF mutations (25/155, 16.1%), and MAP2K1 mutations (23/155, 14.8%) [18]. BRAF is a central kinase in the RAS – RAF – MEK signaling pathway involved in a variety of cellular functions. The BRAF V600E mutation results in constitutive activation of the MAPK pathway. In addition to BRAFV600E, other activating mutations in BRAF, including in-frame deletions, fusions, and duplications, have been reported in LCH lesions [8]. Mutations in MAP2K1, the gene encoding MEK1, or mutations in other genes in the MAPK signaling pathway can be found in 33-50% of patients with BRAF wild-type LCH. Approximately 40% of BRAF wild-type ECD patients carry MAP2K1 mutations [19]. These mutations result in sustained activation of the MAPK signaling pathway.

1.3 Introduction of Study Drug

Based on the structure of Trametinib, a candidate drug candidate for MEK1/2 inhibitor, FCN-159, was designed, synthesized and screened using "structure-based drug design" technology based on the structure-activity relationship (SAR), and a PCT patent (WO 2014169843) was applied for its compound and use, which has been licensed in China and the United States (Chinese Patent No.: 201480021868.8; U.S. Patent No.: US 9,556,171 B2).

Preclinical studies have demonstrated that FCN-159 is an orally available, highly potent MEK1/2 selective inhibitor with promise as a targeted agent for the treatment of advanced solid tumors, neurofibroma type 1, and histiocytic tumors. At present, FCN-159 and its tablets are not marketed in any country, and no other countries or institutions have been reported to study this product. The first-in-human study (Protocol No.: FCN-159-001) evaluating the safety, tolerability, pharmacokinetic profile, and preliminary antitumor activity of FCN-159 in patients with NRAS variant advanced melanoma (Phase 1a) and advanced melanoma with NRAS mutation or NF1 mutation (Phase 1b) has been conducted in China (Protocol No.: FCN-159-001) is a multicenter, open-label, single-arm Ia dose-finding and Ib dose expansion study. A multicenter, open-label, single-arm Phase 1 dose-finding and Phase 2 dose expansion study was also conducted to evaluate the safety, tolerability, pharmacokinetic profile, and antitumor

activity of FCN-159 in patients with neurofibromatosis type 1 (Protocol No.: FCN-159-002). This study will conduct a multicenter, open-label, single-arm Phase 2 study to evaluate the efficacy, safety, and pharmacokinetic profile of FCN-159 in patients with histiocytic tumors (Protocol No.: FCN-159-005).

1.4 Preclinical Studies of FCN-159

To evaluate the in vitro kinase inhibitory activity and selectivity of FCN-159; the in vitro antitumor activity of FCN-159 was evaluated using cell lines with Ras or Raf mutations, and its effects on the proliferation of Ras/Raf wild-type tumor cells and normal cells were also observed; the anti-tumor mechanism of FCN-159 was preliminarily explored; the in vivo antitumor efficacy and preliminary safety of FCN-159 were evaluated in 5 human tumor xenograft models in nude mice. Specific study results are presented below.

1.4.1 Pharmacodynamic Study Results of FCN-159

In vitro kinase activity demonstrated that FCN-159 inhibited activated human MEK1 and MEK2 kinases with IC50 values of 79 and 159.96 nM, respectively, compared to 6.8 and 11.73 nM for Trametinib; fCN-159 inhibited unactivated MEK1 with IC50 values of 5.06 nM and Trametinib of 1.89 nM, respectively. FCN-159 showed weak or no inhibition of the other 40 related kinases tested. FCN-159 is an effective inhibitor of MEK, which mainly inhibits the phosphorylation of MEK, which is weaker than that of activated MEK, and has no inhibitory effect on other kinases.

Mechanism of action studies showed that FCN-159 achieved anti-tumor effects mainly by inhibiting tumor cell growth and proliferation through pharmacological mechanisms such as arresting cell cycle in G0/G1 phase and inducing apoptosis, and the mechanism and effect were similar to those of Trametinib. In human colon cancer Colo205 cells, FCN-159 inhibits or blocks downstream pathways by inhibiting the phosphorylation of MEK kinase, blocks the phosphorylation of ERK proteins in cells, and blocks the G0/G1 phase. FCN-159 induced apoptosis in human colon cancer Colo205 cells in a dose-dependent manner.

In vitro antitumor activity assays showed that FCN-159 had significant in vitro proliferation inhibition on selected tumor cell lines with Raf mutations such as A-375, HT-29, Colo-829, Colo205, SK-MEL-28 or Ras mutations such as SW620, HL-60, Calu-6, A549, with IC50 values ranging from 0.66 to 50 nM, comparable to Trametinib. Among them, A-375, HT-29, Colo-829, Colo205, SK-MEL-28, SW620 and HL-60 were the most sensitive, with IC50 values around 1 nM. At the same time, similar to Trametinib, FCN-159 weakly inhibited the proliferation of Ras/Raf wild-type tumor cell BxPC-3 in vitro, whereas it did not significantly

inhibit the same Ras/Raf wild-type tumor cell H1975. In addition, FCN-159 did not significantly inhibit MRC-5 in normal cells and was similar to Trametinib.

In vivo antitumor activity test showed that FCN-159 significantly inhibited the growth of human tumor xenografts in nude mice in five human tumor xenograft models of human colon cancer COLO205, human melanoma A375, human colon cancer HT-29, human lung cancer Calu-6 and human leukemia HL-60 in a dose-dependent manner. There were some differences in the onset of action doses of FCN-159 in different tumor models, with the most sensitive in the COLO205 model, which was effective at 0.05 mg/kg QD and 0.1 or 0.3 mg/kg QD in other tumor models. In COLO205 and A375 models, FCN-159 partially regressed tumorigenesis at medium and high doses (0.3, 0.6, or 1 mg/kg). The control sample Trametinib (0.6 or 1 mg/kg, ig, QD) showed some anti-tumor effect in the above models and significantly inhibited the growth of human tumor xenografts in nude mice.

The PK/PD correlation study showed that the drug concentration trend of FCN-159 in tumor tissues showed a time-dependent relationship with the inhibition effect of phosphorylation of the downstream kinase ERK, indicating a good correlation between pharmacokinetics and pharmacodynamics; within the tested dose range, the inhibitory effect of FCN-159 on ERK phosphorylation was dose-dependent, consistent with the in vivo anti-tumor drug-efficacy relationship at the same dose.

In summary, FCN-159 selectively inhibited MEK kinase activity, inhibited the proliferation of Ras/Raf mutant tumors, and inhibited the growth of subcutaneous xenografts of multiple human tumors in nude mice, showing good PK/PD correlation; its antitumor effect is comparable to that of Trametinib and has a similar mechanism of action.

1.4.2 Safety Study Results of FCN-159

In the safety pharmacology study, after a single oral gavage administration of FCN-159 to Sprague-Dawley rats, a functional observation battery (FOB) was used to observe the possible effects of FCN-159 on neurobehavior in SD rats. The No Observed Adverse Effect Level (NOAEL) of FCN-159 in rats in the 3 mg/kg group was 1 mg/kg. Single oral gavage administration of FCN-159 to conscious non-restrained SD rats at doses of 0.3, 1, and 3 mg/kg had no significant effect on respiratory function, and the NOAEL of FCN-159 on respiratory function in rats was greater than 3 mg/kg. Single oral gavage administration of FCN-159 to conscious non-restrained Beagle dogs at 0.05, 0.15, and 0.5 mg/kg had no significant effects on 12-lead electrocardiograms and blood pressure. Therefore, the NOAEL of FCN-159 on cardiovascular system function in dogs was greater than 0.5 mg/kg in this study. FCN-159

inhibited the hERG potassium current with an IC50 value of 3.73 μM on CHO cells stably expressing the hERG potassium channel.

A single-dose toxicity study showed that the Maximum Tolerated Dose (MTD) of FCN-159 was 3 mg/kg after a single oral gavage administration to Sprague-Dawley rats. Six (6/10 animals, 1 male and 5 female) and 10 (10/10, 5 male and 5 female) rats in the 10 and 30 mg/kg groups died successively from Day 3 to Day 8 of the study. The above-mentioned dead rats had symptoms of hypoactivity, hunched posture, piloerection, emaciation, yellow discharge in the anogenital area successively from Day 3 of the study. No obvious abnormal changes were observed in any organs or tissues of the above-mentioned dead rats by gross anatomy. Abnormal symptoms associated with dosing were bowed back, piloerection, decreased food consumption, decreased body weight, decreased% LYM, LYM, and increased% NEU, RET, RET. A single oral gavage dose of FCN-159 in Beagle dogs resulted in an MTD of 1 mg/kg. Three dogs (3/4 animals, 1 female and 2 males) in the 10 mg/kg group died successively from Day 3 to Day 6 after administration. Slight to severe hypoactivity, prone position, decreased or no food consumption, and yellow unformed, watery or red/dark red feces were gradually observed in these dead dogs from Day 2 postdose, and vomiting (small amounts of yellow food-like material) was occasionally observed in individual dogs. Gross necropsy showed dark reddish changes in the right anterior, middle and posterior lobes of the lung in 1 (1/3 ratio, male) dog, and moderate hemorrhage, edema and congestion were observed in the lungs of this dog by histopathological examination. Abnormal symptoms related to administration were hypoactivity, prone position, decreased or no food consumption, vomiting and yellow unformed stools, watery or red/dark red/black unformed feces, decreased body weight, decreased thymus volume, severe thymic cortical lymphopenia, increased WBC (mainly NEU increased), decreased RET% and RET, significantly prolonged APTT, and partial abnormalities in blood chemistry parameters, etc.

Repeat-dose toxicity studies showed that FCN-159 was administered once daily by oral gavage to Sprague-Dawley rats at doses of 0.075, 0.15, and 0.3 mg/kg for 4 weeks with a 4-week treatment-free recovery. The lethal dose was 0.3 mg/kg. Its HNSTD was 0.15 mg/kg (AUClast was 2330 h · ng/mL in females and 2390 h · ng/mL in males at this dose). The main target organs of toxicity were immune system (decreased and necrosis of lymphocytes in thymus, spleen, lymph nodes, increased plasma cells and thrombus were observed in lymph nodes, necrosis of red pulp of spleen), hematopoietic system (extramedullary hematopoiesis in spleen, bone marrow necrosis, granulocytosis, erythropenia), digestive system (hepatocellular

necrosis, vacuolar degeneration, atrophy, mineralization of glandular gastric mucosa, dilatation of gastric glands), blood vessels (heart, lung, thrombus), adrenal gland (hypertrophy/degeneration/degeneration of zona fasciculata cells), kidney (tubular mineralization), reproductive system (degeneration/necrosis of spermatogenic cells in testis, cell debris in epididymal duct, atrophy of prostate and seminal vesicle glands, decreased number of corpora luteum luteum), bone (thickening of epiphyseal plate) and skin (skin (skin ulcer, acanthous hypertrophy).At the same time, it was accompanied by the correlative changes of hepatobiliary and renal function indexes, the increase of NEU and MONO in peripheral blood, the decrease of LYM and the symptoms of anemia.)All the above changes were observed in rats in 0.3 mg/kg group; some of the above changes were observed in the 0.075 and 0.15 mg/kg groups, which were milder or occurred in a lower proportion, and generally recovered after 4 weeks off-treatment. In the dose range of 0.075-0.3 mg/kg, the increase in exposure in male rats after the last dose was higher than that after the dose, and was generally dose-proportional in both sexes after the first dose and in female rats after the last dose; there was no significant sex difference in exposure of FCN-159 between male and female rats, and there was significant accumulation after 4 weeks of continuous administration. FCN-159 was administered once daily by oral gavage to Beagle dogs at 0.04, 0.08, and 0.12 mg/kg for 4 weeks with a 4-week treatment-free recovery. Dogs died at 0.08 mg/kg and above with an HNSTD of 0.04 mg/kg (AUClast of 308 h ng/mL in females and 324 h ng/mL in males at this dose). The main target organs of toxicity were immune system (thymus, spleen, lymphoblasts increased plasma cells in lymphoid tissue, decreased lymphocytes and necrosis), digestive system (cholestasis in liver, vacuolar degeneration of hepatocytes, esophageal erosion and ulcer, congestion/hemorrhage in stomach and intestine, glandular cell hypertrophy/hyperplasia, mucosal erosion, intestinal gland dilatation, inflammation), bone marrow (decreased hematopoietic cells, increased granulocyte lineage, decreased erythroid lineage, megakaryocytes), main bronchus (ulcer), skin (hair follicle atrophy), and heart hemorrhage, thrombus and pulmonary thrombus were also observed; at the same time, the serum hepatobiliary, renal and pancreatic indicators were correlated with the increase of NEU, MONO in peripheral blood and anemia symptoms. Some of the above changes were observed in the 0.04 mg/kg group, which were milder and occurred in a lower proportion, and recovered after 4 weeks of drug withdrawal. In addition, no obvious abnormal changes were observed in body temperature, 12-lead electrocardiogram, blood pressure, ophthalmological examination, other hematology, blood biochemistry, urine, bone marrow smear, organ weights and

coefficients, and histopathological examinations of other organs. In the dose range of 0.04 to 0.12 mg/kg, the increase in exposure of FCN-159 in female and male dogs was generally dose proportional, with no significant gender difference, and there was significant accumulation after 4 weeks of continuous administration.

The genotoxicity assay showed that FCN-159 was negative in the Salmonella typhimurium reverse mutation assay (Ames test), the CHL chromosome aberration assay, and the mouse micronucleus assay.

1.4.3 Pharmacokinetic Study Results of FCN-159

FCN-159 was a low clearance drug in both rats and dogs with CLs of 0.22 and 0.08 L/h/kg, respectively. The apparent volume of distribution, Vz, was 7.82 and 5.81 L/kg in rats and dogs, respectively, both exceeding the total volume of fluid in vivo, indicating widespread distribution to tissues. After gavage administration, the absolute bioavailability of FCN-159 in rats (0.075-0.6 mg/kg) and dogs (0.03-0.09 mg/kg) was 56.1% and 61.2%, respectively. The increase in AUC of FCN-159 was generally dose-proportional from 0.075 to 0.2 mg/kg (rats) and 0.03 to 0.09 mg/kg (dogs). The plasma elimination half-lives of FCN-159 in both species were approximately 22.89 to 57.0 h and 42.68 to 64.16 h, respectively. After continuous administration, there was a tendency to accumulate in rats and dogs, which correlated with a long half-life.

After gavage administration of FCN-159 to rats, tissues were widely distributed, with concentrations higher than plasma concentrations in the tissues examined except brain and testes. The main tissues of distribution were stomach, small intestine, large intestine, lung, liver, kidney, spleen, brown fat and white fat, and the exposure of FCN-159 in these tissues exceeded 5 times the plasma exposure; exposure was minimal in brain tissue, only 1/10 of plasma exposure, and exposure in other tissues was 0.4- to 3.5-fold higher than plasma exposure. The plasma protein binding of FCN-159 was 99.598%, 99.597%, 99.694%, 99.643% and 99.653% in human, monkey, dog, rat and mouse, respectively, with no obvious concentration dependence or species difference.

After gavage administration of FCN-159 to rats, extensive metabolism occurred in vivo, with a total of 3 metabolites detected, the main metabolic pathway being monooxidation. Within 120 h after gavage administration, the recoveries of unchanged FCN-159 in feces and urine were 35.93% and 0.00%, respectively, with a total of 35.93%, suggesting that it was mainly excreted as metabolites.

Four metabolites were detected after incubation of FCN-159 in human, monkey, dog, rat

and mouse liver microsomes, respectively; monooxidation was the main metabolic pathway.; the major metabolites in the human liver microsome incubation system were detected in both rat and dog liver microsomes.

FCN-159 strongly inhibited CYP2D6 and did not inhibit other major CYP450 enzymes. FCN-159 did not induce CYP1A2 and CYP2B6 enzymes on human primary hepatocytes, but may have an induction potential towards CYP3A4 enzymes.

FCN-159 has low permeability, and neither Pgp nor BCRP transporters are involved in the transport of FCN-159 on Caco-2 cells.

All chemical, pharmacological, pharmacokinetic and toxicological data are detailed in the Investigator ' s Brochure.

1.5 Clinical Studies of FCN-159

1.5.1 FCN-159-001 Study

FCN-159-001 is the first-in-human (FIH) clinical trial of FCN-159. A total of 9 dose groups of 0.2 mg, 0.5 mg, 1 mg, 2 mg, 4 mg, 6 mg, 8 mg, 12 mg, and 15 mg were explored in the Phase 1a dose escalation part of the trial, and a total of 33 subjects were enrolled. One DLT was observed in the 15 mg dose group, which was Grade 3 folliculitis; no DLTs were observed in the other dose groups. Combined with efficacy and PK data, the RP2D in solid tumors was determined to be 12 mg. In terms of efficacy, confirmed PR was observed from 6 mg, and a total of 21 subjects were included in 6 mg-15 mg, with an ORR of 19% (4/21). Two cohorts were set up in Phase 1b to initially explore efficacy in NRAS-mutated and NF1-mutated melanoma, and the Phase 1b trial is still ongoing.

Preliminary pharmacokinetic results showed that the median time to maximum concentration (Tmax) was 1.00-3.00 h after single doses of 0.2 mg-15 mg. After a single dose, the mean half-life (t1/2) was 31.8-57.4 h in each dose group. Mean clearance (CL/F) ranged from 10.8 to 13.5 L/h (slightly higher CL/F at 0.2 mg and 0.5 mg, possibly related to lower terminal concentrations in both dose groups), with CL/F at essentially the same level across the different dose groups. The mean apparent volume of distribution (Vd/F) ranged from 491 to 1372 L, suggesting that FCN-159 is widely distributed. The Cmax and AUC0-24 of FCN-159 increased generally dose-proportionally from 0.2 mg to 15 mg.

After multiple daily oral doses of 0.2 mg-15 mg, the median Tmax of FCN-159 was 1.25-3.00 h. Mean Cmax and AUC0-τ ranged from 1.17 to 120.17 ng/mL and 12.34 to 1131.77 h * ng/mL, respectively. The drug reached steady state from 8 consecutive days of dosing. The terminal elimination profiles were similar in each dose group after single and multiple doses,

with AUC increased after multiple doses reaching steady state compared with single doses, with an accumulation ratio of approximately 1.5 to 2.8.

1.5.2 FCN-159-002 Study

The Phase 1 dose escalation trial in the NF1 indication was subdivided into two cohorts of adults and children, the first of which was the adult cohort, bridging the results of the FIH trial, exploring a total of 4 mg, 6 mg, 8 mg, and 12 mg dose groups. A total of 3 DLTs occurred in the 12 mg group, all of which were Grade 3 folliculitis, so 12 mg was considered intolerable; no DLTs were observed in the other dose groups and the MTD was determined to be 8 mg. Based on the results of efficacy, PK and E-R analyses, the RP2D of adult NF1 was determined to be 8 mg after discussion at the SMC meeting. Two dose groups of 4 mg/m2 and 5 mg/m2 were explored in the pediatric cohort using the adult RP2D to bridge the dose in the pediatric cohort. Based on safety, efficacy and PK data, 5 mg/m2 was determined as the RP2D for pediatric patients.

Preliminary efficacy data are currently available for the adult NF1 indication, with a total of 19 subjects enrolled in Phase 1 and 63 subjects enrolled in Phase II. As of 28 October 2022, 78 patients had at least one post-treatment response assessment, resulting in an ORR of 30.8% (24/78). The trial is still under further follow-up. The Pediatric NF1 cohort is ongoing.

In terms of safety, safety data up to 09 Aug 2022 in the adult NF1 indication are summarized: of the 82 subjects enrolled, all subjects (82/82, 100%) reported at least one TEAE, with the highest Grade 3, and no Grade 4 or higher TEAEs. The common study drug-related TEAEs (incidence ≥ 20%) were: folliculitis in 56 subjects (68.3%), oral ulcer in 30 subjects (36.6%), diarrhea in 27 subjects (32.9%), alopecia in 22 subjects (26.8%), paronychia in 21 subjects (25.6%), blood lactate dehydrogenase increased in 21 subjects (25.6%), blood alkaline phosphatase increased in 19 subjects (23.2%), tricuspid regurgitation in 17 subjects (20.7%), and mitral valve insufficiency in 17 subjects (20.7%). Study drug-related TEAEs of ≥ Grade 3 were folliculitis in 20 subjects (29.3%).

1.5.3 FCN-159 Food Effect Study

A food effect study of FCN-159 has been conducted in healthy subjects to evaluate the pharmacokinetic (PK) profile of a single oral dose of FCN-159 tablets in healthy subjects under fasting and different meal types and the effect of a high- and low-fat diet on PK. The results showed that the low-fat diet had no effect on the PK of FCN-159, and the 90% confidence intervals (CIs) of the geometric mean ratios (low-fat diet/fasting) of Cmax, AUC0-t, AUC0- ∞

were all within the range of 80% to 125%. Cmax decreased by 15%, but AUC did not change, and the above-mentioned decrease in plasma concentrations was not clinically significant when administered with a high-fat meal compared to fasted subjects. These results support the fasting or fed administration of FCN-159.

1.6 Theoretical Basis of this Clinical Study

FCN-159 has been studied for the first time in humans in Chinese patients with advanced melanoma (Protocol No.: FCN-159-001). 12 mg is the recommended Phase 2 clinical dose for adults with melanoma as assessed by the integrated efficacy, safety, and PK data. In studies in adults and children with neurofibromatosis type 1, 8 mg was designated as the RP2D for adult patients with neurofibromatosis type 1 based on an integrated evaluation of efficacy, safety, and PK data (Protocol No.: FCN-159-002). The recommended adult dose from the neurofibroma study will be used in this study as the therapeutic dose for patients in this study.

Given the important role of MAPK pathway activation in the pathogenesis of LCH and ECD, targeted inhibition of the MAPK pathway may be an effective therapeutic strategy for LCH and ECD. A general metabolic response has been reported in Phase 1-2 clinical trials using the BRAF V600E inhibitor Vemurafenib and in a series of adult LCH and associated Erdheim-Chester disease (ECD) disease (ORR 43% using RECIST v1.1 criteria and 100% using metabolic response criteria for positron emission tomography PET) [20-22]. Discontinuation of treatment often resulted in relapse, and in the LOVE study, 75% of adult patients with ECD progressed after discontinuation of treatment [23]. Importantly, the significant persistence of the response to Vemurafenib in this study suggests that inhibition of the MAP kinase pathway may have a much greater impact on the natural history of histiocytic disease than similar targeted therapies in solid tumors. However, the remaining 50% of LCH patients without BRAFV600 mutations have an unmet need for continued unmet treatment.

Early clinical studies have reported significant efficacy with BRAF or MEK inhibitors in patients with relapsed and refractory LCH and ECD. Patients treated with MAPK pathway inhibitors in early trials of LCH and ECD in adults approached a universal response [22]. Two recently reported retrospective pediatric studies also reported high response rates in cohorts, including children with high-risk LCH, patients with multiple treatment failures, and patients with LCH-ND. However, most patients experience rapid reactivation of disease after cessation of treatment, and reintroduction of BRAF inhibitors is generally effective [24]. Prospective trials are needed to determine the optimal treatment duration and the potential to combine with other targeted or cytotoxic therapies.

FCN-159 is a highly potent and selective MEK1/2 selective inhibitor. FCN-159 can inhibit ERK phosphorylation and RAS-RAF-MEK signaling pathway. The growth of human tumor xenografts in nude mice was significantly inhibited in five human tumor xenograft models of human colon cancer COLO205, human melanoma A375, human colon cancer HT-29, human lung cancer Calu-6 and human leukemia HL-60 in nude mice in a dose-dependent manner, and the efficacy was comparable to that of trimetinib.

In a Phase 2 clinical study (NCT02649972) of the similar product Cobimetinib in the treatment of histiocytic tumors, 18 patients (12 ECD, 2 LCH, 2 Rosai-Dorfman ' s disease, 2 mixed histiocytosis) treated with Cobimetinib resulted in an overall Response rate of 89% (90% confidence interval 73-100) using PET Response Criteria (PRC). The response was durable and no acquired resistance developed. After one year of treatment, 100% of the patients who achieved a response remained durable and 94% were progression-free. Cobimetinib treatment was effective regardless of genotype, with responses observed in patients with mutations in ARAF, BRAF, RAF1, NRAS, KRAS, MEK1 (also known as MAP2K1), and MEK2 (also known as MAP2K2) [25]. The response of histiocytic tumors to Cobimetinib in this study was not only nearly universal (regardless of histiocytic tumor pathological subtype and genotype), but was durable, and no acquired resistance was observed as of study publication. This finding suggests that histiocytic tumors may lack the ability to adapt to MEK1/2 inhibition, whereas MEK1/2 inhibitors may significantly alter the natural history of these diseases. Cobimetinib (Cotellic) received FDA breakthrough treatment designation on 04 October 2019 for the treatment of adult patients with histiocytic tumors that do not carry the BRAF V600E mutation.

Based on the pathogenesis of LCH and ECD, mechanism of action/preclinical pharmacodynamic data of FCN-159 and clinical data of similar products in LCH and ECD, we propose to conduct this clinical study as a multicenter, open-label, single-arm Phase 2 study to evaluate the efficacy, safety and PK of FCN-159 in patients with histiocytic tumors.

The study will be conducted in accordance with the protocol, Good Clinical Practice (GCP), and local regulatory requirements.

2 Study Objectives and Study Endpoints

2.1 Study Objectives

2.2.1 Primary Objective

- To evaluate the efficacy of FCN-159 in patients with Langerhans cell histiocytosis (LCH) and Erdheim-Chester disease (ECD) and other histiocytic tumors.

2.2.2 Secondary Objectives

- To evaluate the safety of FCN-159 in patients with LCH, ECD, and other histiocytic tumors.
- To further assess the efficacy of FCN-159 in patients with LCH, ECD, and other histiocytic tumors in addition to ORR assessed by IRC based on PET Response Criteria (PRC).
- The PK profile of FCN-159 was further evaluated.
- To evaluate the improvement of FCN-159 on quality of life in patients with LCH, ECD, and other histiocytic tumors compared with baseline examinations.

2.2.3 Exploratory Objectives

- To analyze the relationship between MAPK pathway related gene mutation and therapeutic effect.

2.2 Study Endpoints

2.2.1 Primary Endpoints

Objective response rate (ORR) assessed by an Independent Review Committee (IRC) based on PET Response Assessment Criteria (PRC).

2.2.2 Secondary Endpoints

- ORR assessed by the investigator based on PRC response assessment criteria;
- ORR, DCR, CBR, TTR, PFS and OS assessed by IRC and investigator based on RECIST version 1.1 response evaluation criteria.
- To evaluate the frequency and severity of adverse events, serious adverse events, deaths, and safety test abnormalities (e.g., laboratory tests, vital signs, physical examinations, electrocardiograms, ECOG, etc.) according to the National cancer institute common terminology criteria for adverse events (NCI-CTCAE) version 5.0; proportion of patients with dose modification or discontinuation due to drug toxicity.
- Changes in Quality of Life (QOL) EORTC-QLQ-C30 (Version 3.0).
- Population PK parameters of FCN-159.

2.2.3 Exploratory Endpoints

To evaluate MAPK pathway gene mutations and to analyze the correlation with efficacy.

3 Study Plan

3.1 Overall Study Design

A total of approximately 28 patients will be enrolled in this study. This is a single-arm, open-label, multicenter Phase 2 clinical study to evaluate the efficacy, safety, and PK profile of

FCN-159 as a single agent in patients with Langerhans cell histiocytosis (LCH), Erdheim-Chester disease (ECD), and other histiocytic tumors.

It is recommended that patients provide a sufficient number of histopathological slides or tumor tissue samples and peripheral blood for central laboratory testing of the following biomarkers: including but not limited to ERBB3, RAF-1, BRAF, ARAF, HRAS, KRAS, NRAS, MEK (MAP2K1 and MAP2K2), and other MEK upstream genes.

A total of 28 patients with LCH, ECD, and other histiocytic tumors will be included in this study, and the diagnosis will be reviewed by the central pathology of the leading unit. FCN-159 will be administered at a dose of 8 mg/day orally once daily as a single agent in a 28-day treatment cycle until disease progression, death, intolerable toxicity, patient withdrawal of consent, or end of study.

Tumor Response will be evaluated once at screening, at the end of 3rd, 6th, 12th treatment cycles (± 7 days), and at the end of treatment by IRC and investigator according to PET Response Criteria (PRC). Patients evaluated as CMR or PMR had confirmed response by repeated assessments at least 4 weeks later. At screening, at the end of the 3rd, 6th, 9th, 12th treatment cycles (± 7 days), and at the end of treatment as assessed by the IRC and investigator according to RECIST v1.1. If the imaging results are considered to be consistently stable by the Principal Investigator after 12 treatment cycles, tumor assessments will be performed every 4 treatment cycles (± 14 days) according to RECIST 1.1, if the imaging results do not reach sustained stability in the opinion of the investigator after 12 treatment cycles, tumor assessments will be performed every 3-6 treatment cycles (± 14 days) according to PRC criteria, and tumor assessments will be performed every 3 treatment cycles (± 7 days) according to RECIST 1.1. FCN-159 treatment lasted up to 2 years. Quality of life and MAPK pathway gene mutation detection will be assessed at the same time as RECIST 1.1 tumor assessment.

The study will also evaluate patient safety, mainly including monitoring and recording AEs and SAEs, performing protocol-specified laboratory tests (including hematology, blood biochemistry, urinalysis, etc.), 12-lead ECG, ophthalmology, vital signs, physical examination, etc. The follow-up period includes safety follow-up and survival follow-up. This study will also further evaluate the PK profile of FCN-159 in patients with histiocytic tumors.

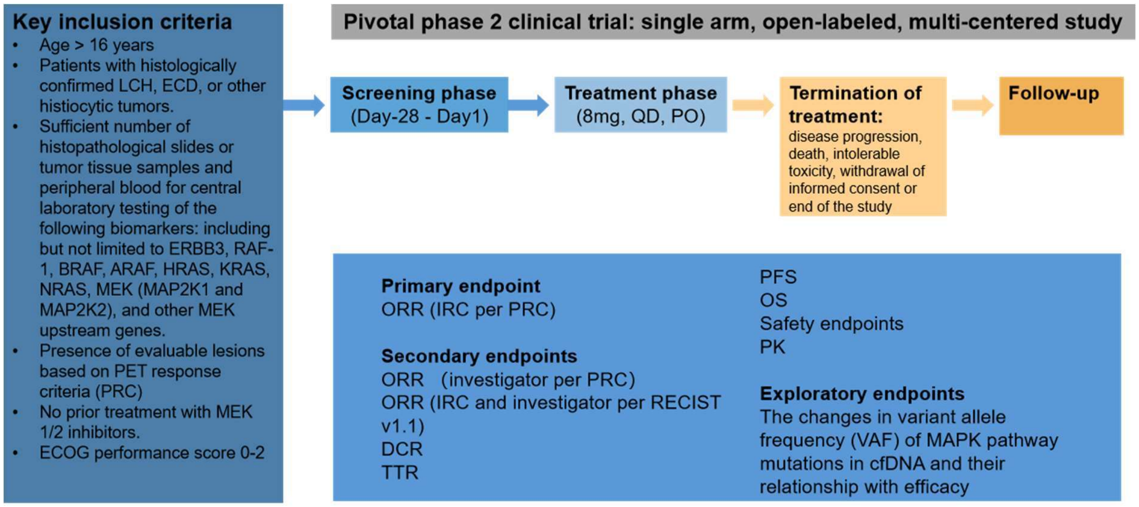

Figure 2 Overall Study Design Protocol

3.2 Tumor Assessment

Histiocytic tumor lesions will be evaluated by whole-body 18FDG-PET/CT scan or MRI/CT scan according to PET Response Assessment Criteria (IRC) and RECIST v1.1, respectively. Tumor response will be evaluated once at screening, at the end of 3rd, 6th, 12th treatment cycles (± 7 days), and at the end of treatment by IRC and investigator according to PET response assessment criteria. Patients evaluated as CMR or PMR had confirmed response by repeated assessments at least 4 weeks later. Tumor assessments will be performed by the IRC and the investigator at screening, at the end of 3rd, 6th, 9th, 12th treatment cycles (± 7 days), and at the end of treatment according to RECIST 1.1 criteria. If the imaging results are considered to be consistently stable by the Principal Investigator after 12 treatment cycles, tumor assessments will be performed every 4 treatment cycles (± 14 days) according to RECIST 1.1, if the imaging results do not reach sustained stability in the opinion of the investigator after 12 treatment cycles, tumor assessments will be performed every 3-6 treatment cycles (± 14 days) according to PRC criteria, and tumor assessments will be performed every 3 treatment cycles (± 7 days) according to RECIST 1.1. FCN-159 treatment lasted up to 2 years. If a patient discontinues treatment, tumor assessments should continue as scheduled until disease progression, death, withdrawal of informed consent, loss to follow-up, start of new anti-tumor therapy, or end of the study.

3.3 End of Study

The end of study is defined as 2 years after the last patient ' s first dose of FCN-159 or the last patient ' s last dose of FCN-159, whichever occurs earlier.

4 Selection of Study Subjects

4.1 Inclusion Criteria

The following conditions must be met for patient enrollment:

1. Age > 16 years.
2. Patients with histologically confirmed LCH, ECD, or other histiocytic tumors.
3. A sufficient number of histopathological slides or tumor tissue samples and peripheral blood will be available for central laboratory testing of the following biomarkers: including but not limited to ERBB3, RAF-1, BRAF, ARAF, HRAS, KRAS, NRAS, MEK (MAP2K1 and MAP2K2), and other MEK upstream genes.
4. Patients with LCH require multisystem involvement (greater than 1 system) or single-system multifocal disease (greater than 1 lesion).
5. Presence of evaluable lesions based on PET response criteria (PRC).
6. Patients with treatment-naïve or relapsed, refractory LCH, ECD, or other histiocytic tumors may be included.
7. Expected survival is at least 3 months.
8. ECOG performance score 0-2, see Attachment 4.
9. Ability to understand and voluntarily sign written informed consent.
10. Premenopausal women had a negative serum human chorionic gonadotropin (HCG) pregnancy test within 7 days prior to initiation of dosing, and a serum human chorionic gonadotropin (HCG) pregnancy test may not be performed in surgically infertile or non-fertile women ≥ 1 year postmenopausal.
11. For patients of childbearing potential: patients should agree to use effective contraception, double-barrier contraception, condoms, oral or injectable contraceptives, intrauterine devices, etc. during the treatment period and for at least 90 days after the last dose of study treatment. Male patients should agree to refrain from donating sperm for at least 90 days after the last dose.
12. Adequate bone marrow function: absolute neutrophil count ≥ 1.5 × 109/L, hemoglobin ≥ 90 g/L, and platelets ≥ 100 × 109/L without transfusion and blood products and granulocyte colony-stimulating factor. Patients with cytopenia due to the disease itself below these

thresholds may be waived from enrollment based on the investigator ’ s comprehensive judgment.

13. Adequate liver and kidney function: Aspartate aminotransferase (AST), Alanine aminotransferase (ALT) ≤ 2.5 × Upper limit normal (ULN), and ≤ 5 × ULN if liver invasion occurs; serum total bilirubin ≤ 1.5 × ULN, ≤ 5.0 × ULN in patients with Gilbert ’ s syndrome or liver invasion; alkaline phosphatase (AKP) ≤ 2.5 × ULN, or ≤ 10 × ULN if liver invasion occurs; serum creatinine ≤ 1.5 × ULN, and if serum creatinine > 1.5 × ULN, creatinine clearance (CCr) ≥ 50 mL/min (based on Cockcroft-Gault formula, see Attachment 6); albumin ≥ 3 g/dL.

14. Coagulation function: international normalized ratio (INR) and activated partial thromboplastin time (APTT) ≤ 1.5 ULN.

4.2 Exclusion Criteria

1.Patients with one of the following prior therapies:

a. Chemotherapy, targeted therapy, immunotherapy, biotherapy, investigational therapy, or herbal anti-tumor therapy for histiocytic tumors within 4 weeks or < 5 half-lives (whichever is longer) before starting study drug.

b. Received strong CYP3A4, CYP2C8, CYP2C9 inhibitors or inducers within 14 days prior to starting study drug, except for topical use in the skin.

c. Use of growth factors that promote platelet or leukocyte numbers or function within 7 days prior to starting study drug.

d. Radiation therapy and surgery within 4 weeks prior to starting study drug.

e. Patients who have participated in other interventional clinical trials within 4 weeks prior to starting study drug.

f. Prior treatment with MEK 1/2 inhibitors.

g. High-dose chemotherapy and stem cell transplant salvage (autologous stem cell transplant) or allogeneic stem cell transplant within 90 days prior to enrollment. Patients receiving posttransplant anti-Graft Versus Host Disease (GVHD) drugs such as cyclosporine, tacrolimus, or other drugs to prevent GVHD after BMT cannot participate in this trial.

h. For patients with brain tumors (intracranial masses), anticoagulants will be used within 7 days prior to starting study drug.

i. Prednisone treatment < 0.5 mg/kg/day (or equivalent dose of other corticosteroids) within one month prior to enrollment is allowed, but must be stopped 14 days before

starting study drug. Patients with brain lesions treated with corticosteroids for brain edema must remain on a stable dose for 14 days prior to enrollment. Hormone replacement therapy is permitted in patients with hypopituitarism due to primary pituitary involvement.

2.The patient had a history or concurrent malignancy of other malignancies (excluding cured non-melanoma basal cell carcinoma of the skin, carcinoma in situ of the breast or carcinoma in situ of the cervix, and other malignancies without evidence of disease within 5 years).

3.Uncontrolled stable hypertension (with medical therapy): systolic blood pressure > 160 mm Hg and/or diastolic blood pressure > 100 mm Hg.

4.The patient had dysphagia, active digestive disease, malabsorption syndrome, or other conditions that affected the absorption of study drug.

5.Prior or current retinal vein occlusion (RVO), retinal pigment epithelial detachment (RPED), glaucoma, and other significant abnormalities.

6.Interstitial pneumonia, including clinically significant radiation pneumonitis. Interstitial pneumonia due to primary pulmonary involvement is excluded.

7.Cardiac function or comorbidities that meet one of the following conditions will be excluded:

a. Three 12-lead Electrocardiogram (ECG) measurements will be performed at the study site at screening, and the mean of three times will be calculated according to the QTcF formula of the instrument, and the QTcF is > 470 milliseconds; for patients with risk factors for QTcF prolongation, such as inability to correct hypokalemia, hereditary long QT syndrome; or receive drugs that prolong the QTcF interval (mainly class Ia, Ic, III antiarrhythmic drugs). Drugs with the potential to prolong the QTcF interval are listed in Attachment 8.

b. New York Heart Association (NYHA) grade ≥ 3 congestive Heart failure, see Annex 5;

c. Clinically significant arrhythmias, including, but not limited to, complete left bundle branch conduction abnormalities, second-degree atrioventricular block;

d. Known concurrent clinically significant coronary heart disease, cardiomyopathy, severe valvular disease.

e. Echocardiography revealed a left ventricular ejection fraction (LEFV) < 50%.

8.With active bacterial, fungal, or viral infection, including active hepatitis B (hepatitis B virus surface antigen positive and hepatitis B virus DNA more than 1000 IU/ml or meeting site criteria for diagnosis of active hepatitis B infection) or hepatitis C (hepatitis C virus RNA positive), human immunodeficiency virus infection (HIV positive).

9.Pregnant or lactating women.

10.Known hypersensitivity to study drug, other MEK1/2 inhibitors or other excipients.

11.Clinically significant circumstances that, in the opinion of the investigator, would preclude participation in the study or inability to comply with safety requirements.

4.3Treatment Discontinuation and Patient Withdrawal from the Study

4.3.1 Reasons for Discontinuation

Patients have the right to discontinue treatment at any stage of the study. Patients may be discontinued from treatment at any stage of the study for any of the following reasons:

(1) Disease progression;
(2) Death;
(3) Pregnancy, breastfeeding or intentional breastfeeding;
(4) Intolerable toxicity related to study treatment;
(5) Other adverse events;
(6) The investigator requests that the patient discontinue treatment prematurely in the best interest of the patient;
(7) Withdrawal of consent or request by his/her legal representative to discontinue treatment;
(8) Patients were lost to follow-up for more than 3 months;
(9) Notification by the Sponsor of the termination of the study;
(10) Another systemic anti-tumor therapy was used;
(11) Major protocol violation.

4.3.2 Management of Patient Discontinuation

The reason for premature discontinuation should be recorded in the original medical record and in the eCRF.

If a patient discontinued treatment prematurely due to an AE (including an SAE) or a clinically significant abnormal laboratory finding, the investigator had to follow the patient until the AE or abnormal value resolved or stabilized or could not be followed.

Subsequent anti-tumor therapy and survival will be collected every 6 months (± 14 days) with the first safety visit or end of treatment visit as the starting point.

After discontinuation of treatment, patients should follow the protocol-specified follow-up schedule to complete efficacy and safety assessments for discontinuation and complete the safety follow-up visit and the outcome of all AEs, concomitant medications, and treatment.

After discontinuation of treatment, tumor assessments will continue as scheduled until disease progression, death, withdrawal of informed consent, loss to follow-up, start of new

anti-tumor therapy, or the end of the study.

4.3.3 Replacement of patients who discontinued study treatment

Patients enrolled in this study will not be replaced.

4.3.4 Withdrawal of Subjects from the Study

Reasons for withdrawal may include:

(1) Death;

(2) Withdrawal of consent or withdrawal by his/her legal representative;

(3) Lost to follow-up for more than 3 months;

(4) The investigator requests that the patient discontinue the study prematurely in the best interest of the patient;

(5) Notification by the Sponsor of the termination of the study;

5 Study Procedures

5.1 Screening/Baseline Period (Day -28 to -1)

Patients will be required to sign and date a written ICF before any study-specific procedures, sampling, and analyses are performed. Reexamination may be performed during the screening period, with the results of the last reexamination as the basis for enrollment. If a corresponding repeat result is obtained during the screening period, no new patient screening number is required; repeat screening test results will be recorded on the appropriate CRF (unscheduled laboratory forms). If a corresponding repeat laboratory result is not available during the screening period, the patient is considered a screening failure and all screening procedures and tests need to be repeated using a new patient screening number.

- Demographic data: including date of birth, gender, race, ethnicity, alcohol consumption history, and smoking history.
- Study Inclusion/Exclusion Criteria Assessment.
- Past medical history.
- Past tumor history: including date of tumor diagnosis, start/end date of prior regimen, date of disease progression. Previous meaningful procedures should be recorded in the eCRF, including start and end dates, name and site of the procedure.
- Physical examination: including height, weight, head, eyes, ears, nose, throat, neck, heart, chest, abdomen, extremities, skin, lymph nodes, nervous system and general condition of the patient.
- Vital signs: blood pressure, heart rate, respiration, body temperature. Blood pressure

should be measured after the patient has been sedentary for 5 minutes.

- Performance score: ECOG score, see Attachment 4.
- Laboratory tests: including hematology, blood chemistry, urinalysis, and coagulation tests.
    - Hematology: red blood cell count, hemoglobin, hematocrit, white blood cell count and differential (neutrophils, lymphocytes, eosinophils, monocytes, basophils, and other cells) and platelet count.
    - Blood chemistry: total protein, albumin, blood glucose, total cholesterol, triglycerides, urea/urea nitrogen, creatinine, alkaline phosphatase, lactate dehydrogenase, gamma-glutamyl transpeptidase, total bilirubin, direct bilirubin, AST, ALT, calcium, phosphorus, magnesium, potassium, sodium, chloride, serum amylase, erythrocyte sedimentation rate, C-reactive protein, creatine phosphokinase (CK), CK-MB, uric acid.
    - Urinalysis: specific gravity, pH, urine glucose, urine protein, ketones, red blood cells, and white blood cells. If urine protein is positive at screening, a quantitative 24-hour urine protein test is required.
    - Coagulation tests: PT, APTT, INR.
- Echocardiography or MUGA: particular attention should be paid to evaluation of left ventricular ejection function.
- 12-lead ECG: after resting for at least 5 minutes in semi-recumbent or supine position, ECGs will be performed by qualified central staff and three consecutive ECGs are required.
- HBV, HCV, and HIV testing: testing for HBV, HCV, and HIV is required. Five items of hepatitis B and five items of hepatitis B should be tested for HBV: HBsAg, HBsAb, HBeAg, HBeAb and HBcAb. HBV DNA copy number testing is required for patients who are positive for HBsAg, HBeAg, HBeAb, and HBcAb, and hepatitis C virus RNA (HCV RNA) is required for HCV-IgG positive patients.
- Blood HCG test (if applicable).
- PRC standard assessment: 18FDG-PET/CT12 examination range from skull top to plantar. Tumor imaging will be assessed by the investigator and IRC according to PRC, respectively. The number of tumor assessments may be increased by the investigator as clinically indicated. The investigator should make subsequent treatment decisions based on his/her response evaluation results. Tumor assessments may be used as baseline tumor

assessments if they have been performed within 28 days prior to the first dose and the same method and machine are used in the same hospital.

- RECIST v1.1 criteria: including MRI/CT examinations of the head, neck, chest, abdomen and pelvis. Target lesions are suggested by PET-CT or new lesions during treatment are selected or added, but the same imaging assessment method (MRI/CT) should be used for each patient throughout the treatment. Tumor imaging will be assessed by the investigator and IRC according to RECIST v1.1, respectively. Tumor assessments may be performed more frequently by the investigator as clinically indicated. The investigator should make subsequent treatment decisions based on their own response evaluation results. Tumor assessments may be used as baseline tumor assessments if they have been performed within 28 days prior to the first dose and the same method and machine are used in the same hospital.
- Quality of life assessment: the quality of life scale is presented in Attachment 3.
- Ophthalmologic examination: including visual acuity, visual field, slit lamp fundus examination, intraocular pressure, fundus photography/scanning laser fundus examination, and ophthalmologic B-ultrasound.
- Prior to enrollment, patients should provide a sufficient number of histopathological slides or tumor tissue samples for the purpose of MAPK pathway related gene mutation testing, including but not limited to ERBB3, RAF-1, BRAF, ARAF, HRAS, KRAS, NRAS, MEK (MAP2K1 and MAP2K2), and other MEK upstream genes. Additionally, peripheral blood should also be collected for the detection of MAPK and other pathway gene mutations in cfDNA.
- Concomitant Medications/Concomitant Therapy: record from 28 days prior to the first dose until 30 days after the last dose or until the start of other anti-tumor therapy, whichever occurs first.
- Adverse Events: collect from signing the informed consent form until 30 days after the last dose or until the start of other anti-tumor therapy, whichever occurs first. Study drug-related AEs/SAEs should also be collected and reported after the endpoints of AE/SAE collection.

5.2 Continuous Dose Period (CXD1 ± 3 days)

- Physical examination.
- Vital signs.
- Performance score.

- Hematology, blood biochemistry, urinalysis.
- Coagulation tests: as clinically indicated.
- Echocardiography or MUGA with special attention to evaluation of left ventricular ejection function: as clinically indicated.
- 12-lead ECG: three ECGs will be performed by qualified center staff after the patient has rested for at least 5 minutes in semi-recumbent or supine position.
- Ophthalmologic examination: perform in parallel with RECIST 1.1 tumor assessment.
- Assessment of PRC criteria: tumor response will be evaluated once at screening, at the end of 3rd, 6th, and 12th treatment cycles (± 7 days) and at the end of treatment by IRC and investigator according to PRC criteria, respectively. Patients evaluated as CMR or PMR had confirmed response by repeated assessments at least 4 weeks later. Tumor assessments per PRC criteria will be performed every 3-6 treatment cycles (± 14 days) if, in the opinion of the investigator, the imaging results are not consistently stable after 12 treatment cycles. If a patient discontinues treatment, tumor assessments should continue as scheduled until disease progression, death, withdrawal of informed consent, loss to follow-up, start of new anti-tumor therapy, or end of the study.
- RECIST Version 1.1 Criteria Assessment: tumor assessments will be performed by the IRC and the investigator at screening, at the end of 3rd, 6th, 9th, 12th treatment cycles (± 7 days), and at the end of treatment according to RECIST v1.1 criteria, respectively. Tumor assessments will be performed according to RECIST 1.1 every 4 treatment cycles (± 14 days) if the imaging results are considered to be consistently stable by the Principal Investigator after 12 treatment cycles, and every 3 treatment cycles (± 7 days) if the imaging results do not reach sustained stabilization in the opinion of the investigator after 12 treatment cycles. If a patient discontinues treatment, tumor assessments should continue as scheduled until disease progression, death, withdrawal of informed consent, loss to follow-up, start of new anti-tumor therapy, or end of the study.
- Quality of life assessment: the assessment is the same as the RECIST 1.1 tumor assessment visit timepoint.
- Concomitant medications/concomitant therapies.
- Evaluation of AEs and SAEs.
- PK blood sample collection, see Attachment 1.
- Peripheral blood will be collected at the same time point as RECIST v1.1 tumor assessment visit for the detection of MAPK and other pathway gene mutations in cfDNA.

- Dispensing and recovery of study drug: the study drug should be dispensed to the patient on the first day of each cycle. In the new cycle, the study drug should be dispensed after completing assessments including laboratory tests, etc., and it is confirmed that the patient is safe to continue to take the study drug. The patient should retain all unused drugs and bottles and return them at the next visit.

5.3 End of Treatment (± 7 days)

- Physical examination.
- Vital signs.
- Performance score.
- Hematology, blood chemistry, urinalysis.
- 12-lead ECG: patients will undergo 3 ECGs by qualified center staff after resting for at least 5 minutes in semi-recumbent or supine position.
- Echocardiography or MUGA with special attention to evaluation of left ventricular ejection function: as clinically indicated.
- Coagulation tests: as clinically indicated.
- Blood HCG test (if applicable).
- Ophthalmologic examination: perform in parallel with RECIST 1.1 tumor assessment.
- Assessment of PRC criteria: at the End of Treatment Visit, if tumor imaging has been performed in the first 8 weeks, tumor imaging may not be repeated at the End of Treatment Visit.
- RECISIT 1.1 Criteria Assessment: if tumor imaging has been performed in the first 4 weeks at the End of Treatment Visit, tumor imaging may not be repeated at the End of Treatment Visit.
- Quality of life assessment.
- Peripheral blood will be collected for the detection of MAPK and other pathway gene mutations in cfDNA.
- Recovery of study drug.
- Concomitant medications/concomitant therapies.
- Adverse events.

5.4 Safety Follow-up

The Safety Follow-up Visit is within 30 days (± 7 days) after the last dose, and if the patient starts subsequent anti-tumor therapy within 30 days after the last dose, the post-treatment Safety Follow-up Visit should be completed before the patient starts a new anti-

tumor therapy. If a patient is unable to return to the study site for a safety visit due to physical condition, a remote visit may be performed and the local test results may be acceptable.

- Physical examination.
- Vital signs.
- Hematology, blood chemistry, urinalysis.
- 12-lead ECG: after resting for at least 5 minutes in semi-recumbent or supine position, ECGs performed 3 times by qualified central staff.
- Coagulation tests: as clinically indicated.
- Color echocardiography or MUGA: as clinically indicated.
- Ophthalmic examination.
- Concomitant medications/concomitant therapies.
- Adverse events.

5.5 Survival Follow-up

Subsequent anti-tumor treatment information and survival will be collected every 6 months (± 14 days) until the end of the study, starting from the end of treatment or the safety follow-up visit, whichever occurs later.

6 Study Drug and Study Methods

6.1 Study Drug

The clinical dosage forms of FCN-159 are tablets in strengths of 1.0 mg/tablet and 4.0 mg/tablet and are administered orally.

6.2 Packaging and Labeling of Study Drug

Drug labels will be designed in accordance with national drug labeling requirements and will include information such as drug packaging, name, strength, storage conditions and manufacturing date, but will not contain patient privacy information.

6.3 Provision and Storage of Study Drug

The sponsor will provide study drug. The study drug can only be used for patients enrolled in the study.

The study site will delegate a dedicated person to manage the study drug. The study drug should be stored in tightly closed containers, protected from light, and stored below 25 °C. The investigator/pharmacist is responsible for the storage of the study drug and performs temperature testing.

6.4 Drug Compliance Assessment

Drug counts and patient compliance will be assessed by maintaining complete "Drug

Dispensing" and "Return Record Sheets".

At the end of each cycle of treatment (i.e., at the next visit), patients will be asked to return all used and unused drug packages as an assessment of compliance. A patient study medication diary will be provided to record medication details of the study drug.

Drug dispensing records must be kept up to date and contain the following:

- Determine the identity of the patient before dispense study drug
- Date and number of study drug dispensed to patients
- Date and number of study drug returned by patient

Inventory records must be provided for inspection by the monitor. At the end of the study, all drug supplies, including partially used or empty packages and dosing logs, must be returned for inspection by the monitor.

6.5 Doses and Cycles Administered

Patients will receive oral FCN-159 tablets, bridging the recommended adult dose of 8 mg in the neurofibroma study as the therapeutic dose for this study once daily in a 28-day cycle.

6.6Method of Administration

Patients must take the drug in accordance with the following requirements:

(1) The drug should be taken at the same time every day as possible, and on the day of PK blood sampling, the patient must come to the study site and take the drug under the guidance of the study personnel;

(2) Take FCN-159 tablets with about 200 mL of warm water each time and swallow all tablets as soon as possible;

(3) Patients must swallow intact tablets to avoid chewing;

(4) If vomiting occurs after taking the drug, no additional medication is required, and wait until the next dose is taken;

(5) If you miss the scheduled time, you should take it as soon as possible on the same day. Missed scheduled doses will not be resupplied if ≤ 8 hours from the next scheduled dose. Missed doses must be reported to the investigator and recorded in the medication diary card;

(6) Patients will be required to record daily dosing on a dosing diary card and return to the study personnel at the return visit.

The investigator must instruct the patient to take the study drug. All prescribed and dispensed medications and dose modifications must be documented in the designated case records and entered in the eCRF.

6.7Concomitant Medications

All concomitant medications and treatments (including prophylactic medications) and reasons for use during the study must be recorded in the original medical records. Antineoplastic therapy prior to screening, including drug therapy, radiotherapy, and surgery, will be recorded in the medical history.

Necessary supportive care and medications, such as antiemetics, antidiarrheals, etc., are allowed. Any concomitant medications and non-drug therapies (including physical therapy or blood transfusions) should be recorded in the medical records during the study.

6.8 Permitted and Prohibited Medications and Non-pharmacotherapies

6.8.1 Permitted Medicinal and Non-pharmacological Therapies

Patients should receive adequate supportive care during the study, including transfusions and blood products, antibiotic therapy, antiemetics, antidiarrheals, analgesics, and other applicable therapies that meet local treatment guidelines.

6.8.2 Prohibited Medications and Non-pharmacotherapies

Patients should be prohibited from receiving other anti-tumor therapy while on study treatment. Patients should not receive any other anti-tumor drugs within 28 days or 5 half-lives, whichever is longer, prior to the first dose.

Preclinical pharmacokinetic studies showed that the major metabolic pathway of FCN-159 in human liver microsomes was monooxidation, catalyzed by CYP3A4, CYP2C8, and CYP2C9 enzymes. Therefore, the use of strong inducers or inhibitors of CYP3A4, CYP2C8, and CYP2C9 in clinical trials may affect the in vivo metabolic process of FCN-159 and should therefore be prohibited. Drugs that potentially prolong the QTc interval should be avoided during the study unless there is no alternative treatment. If such drugs must be used, written approval from the study and/or the sponsor is required. Drugs with the potential to prolong the QTc interval are listed in Attachment 8.

Table 3 Study Prohibited Medications

Drug Classification	Specific Drug	Washout Period
CYP3A4 Strong inhibitor	Amprenavir, atazanavir, boceprevir, clarithromycin, conivaptan, delaviridine, diltiazem, erythromycin, furosanavir, indinavir, ketoconazole, itraconazole, lopinavir, mibefradil, miconazole, nefazodone, nelfinavir, posaconazole, ritonavir, saquinavir, telithromycin, verapamil, voriconazole, ritonazole, ritonavir, telithromycin	14 days
CYP3A4 Strong inducers	Carbamazepine, felbamate, nevirapine, phenobarbital, phenytoin, primidone, rifabutin, rifampin, rifapentine	14 days

CYP2C8 Strong inhibitor	Clopidogrel, gemfibrozil	14 days
CYP2C8 Moderate inducer	Carbamazepine, Avonib, Rifampin	14 days
CYP2C9 Strong inhibitor	Sulfaphenazole, tasolam	14 days
CYP2C9 Moderate inducer	Carbamazepine, Enzalutamide, Rifampin, Ritonavir	14 days

Note: this list does not contain all drugs and if future changes are made to the list of prohibited drugs, they will be kept in the form of an official document along with the study file.

6.8.3 COVID-19 Treatment and Vaccination

Treatment with COVID-19:

Emergency use of licensed interventions (redesivir, convalescent plasma, monoclonal antibodies, etc.) is permitted. Antibodies and plasma may interact less with the study drug, and redecivir (theoretically) may result in an increased risk of allergic reactions. Therefore, these treatments should be used with caution and adverse reactions monitored.

COVID-19 Vaccination:

There are currently no data supporting the interaction of the COVID-19 vaccine with the FCN-159 drug. Considering the current types of COVID-19 vaccines and the provisions of the clinical center COVID-19 guidelines, it is recommended to consider comprehensively the effect of the investigational drug on the immune function of patients and the requirements for an appropriate population to be vaccinated.

1.Screening: patients should not receive live vaccines (e.g., adenoviral vector vaccines) within 28 days prior to the first dose of study drug. Inactivated vaccines and mRNA vaccines should not be administered within 14 days prior to the first dose of study drug.

2.During study treatment: patients are not recommended to receive any form of novel coronavirus vaccine during the course of study treatment. In particular, live vaccines should be avoided.

3.Vaccination should be recorded on the concomitant medications page of the eCRF.

Other vaccinations:

All vaccines are available except live vaccines. It should be recorded on the concomitant medications page of the eCRF. Vaccination should preferably be scheduled before enrollment or after the end of study drug treatment.

6.9Treatment Compliance

The investigator should timely and accurately record the quantity, date and actual dose of study drug dispensed/recovered by each patient, and the actual dose taken should be consistent with the dose required by the protocol. Medication compliance will be judged based on the number of medications dispensed and returned to the patient at the end of each treatment cycle and at the time of withdrawal from the study, and the number of drugs lost by the patient. and comprehensive judgment of missed/overdose/drug loss reported by the patient.

Patients will be asked to return all used or unused study drug bottles to the study site after the end of treatment to evaluate compliance. At the end of the study, all remaining items and drugs must be returned to the Sponsor.

Noncompliance is defined as patients who receive less than 80% or greater than 120% of planned medications during study treatment.

6.10Management Principles of Adverse Reactions Occurring During the Study

Patients with NCI-CTCAE Version 5.0 Grade 3 or higher and/or unacceptable toxicity events will be considered at least possibly related to study treatment. Study medication needs to be discontinued and supportive care will be given as per local treatment routine. If the toxicity recovers to ≤ NCI-CTCAE Grade 2 or lower within 28 days of onset and the patient has not progressed or the investigator believes the patient can benefit from study treatment, the original dose of study drug may be continued or the dose may be reduced after discussion with medical monitoring. If the patient recovers within 28 days and does not progress, the study drug may be re-administered at a reduced dose at the time of Grade 3 toxicity, at which time, the dose that has been explored at the time of dose escalation will be reduced to 8 mg, 6 mg, and 4 mg, respectively. In the event of dose interruption or dose reduction, the cycle will not be adjusted.

If a patient experiences relevant toxicities and side effects during the study, the following principles should be followed for the management of toxic and side effects, dose interruption and dose reduction.

6.10.1 Dermal toxicity

Skin toxicity is one of the most common adverse reactions of MEK inhibitors in adults and children, and skin toxicity is generally observed within 2 weeks of starting treatment with Trametinib. In the METRIC study [26-27], the overall incidence of any skin toxicity was 87% in patients treated with Trametinib, the most common of which were rash, dermatitis acneiform, palmar-plantar erythrocyte dysesthesia syndrome, and erythema. Serious skin toxicities

occurred in 12% of patients treated with Trametinib, with 6% requiring hospitalization for dermatologic toxicity. Dose reduction was required in 12% of patients with skin toxicity and permanent discontinuation of Trametinib was required in 1% of patients with skin toxicity.

Selmetinib, a MEK1/2 inhibitor, is the first molecule to be shown to have the ability to inhibit PN growth. In the SPRINT study, rash occurred in 91% of 74 pediatric patients who received selumetinib. The most common rash included rash acneiform (54%), maculopapular rash (39%), and eczema (28%). Grade 3 rash occurred in 8% of patients. Rash led to dose interruption in 11% of patients and dose reduction in 4% of patients [28].

Preventive and control measures:

- Avoid excessive sun exposure and use sufficient sunscreen in anticipation of sun exposure;
- Physical examinations, including skin, will be performed continuously in clinical studies to pay attention to skin abnormalities;
- For Grade 2 intolerable or Grade 3 or higher skin toxicity, consult dermatology and/or consider interruption; if the patient cannot recover to Grade 2 or lower within 4 weeks, the investigator will decide whether to permanently discontinue the drug after comprehensively judging the benefits and risks of the patient;
- Management of skin toxicity is shown in the following figure:

General skin care recommendations (all patients)

- Bath twice daily with a mild bath and high-quality skin emollients and apply to the entire body within 5 minutes of each bath.
- Bleach baths 3 to 4 times a week (1/4 to 1/2 cups of bleach poured into the bathtub, or 1 teaspoon per gallon, soak for 5-10 minutes, then rinse with water and apply emollients).
- Avoid fragrant products such as soaps, detergents, and scented creams.
- Sunscreen: apply zinc oxide or titanium dioxide sunscreen outdoors every 2-3 hours using sunscreens, such as wide-brimmed hats and light clothing, and apply sunscreen to your face every day, even if you do not intend to go outdoors.

Acne prophylaxis (all postpubertal patients)

Pimecrolimus and clindamycin 1% cream twice daily were applied to zone T.

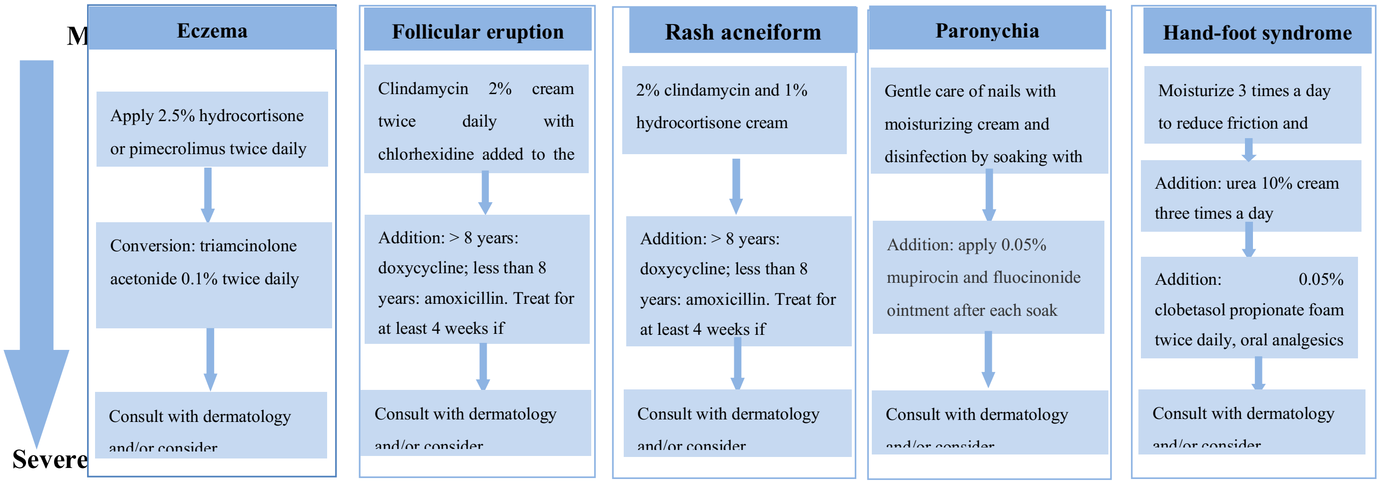

6.10.2Serious Hepatotoxicity

Preclinical studies with FCN-159 have shown that FCN-159 is hepatotoxic, such as hepatocellular necrosis, vacuolar degeneration, atrophy, and hepatobiliary function-related changes.

Hepatic adverse events have been reported in clinical trials of Trametinib [26-27]. Of the hepatotoxicity events, ALT and AST increased were the most common events, with more than 90% of hepatic events occurring within the first 6 months of treatment with Trametinib monotherapy. Currently, few adverse events of liver enzyme increased and bilirubin increased have been reported in the Phase 1 clinical study of FCN-159, and no Grade 3 or higher adverse events have been reported.

Once Grade 3 ALT/AST increased occurs, interrupt dosing until recovery to below Grade 2. If resolved within 7 days, resume treatment at the same dose level. If recovery occurs between 7 and 21 days, continue dosing until recovery is reduced to the next dose level studied. Permanently discontinue if not recovered within 21 days. If Grade 3 ALT/AST increased recurs, hold until recovery to Grade 2 (permanently discontinue if not resolved within 7 days). In the event of > Grade 3 ALT/AST elevation, assessment is required:

- Detailed medical history: collect relevant information such as alcohol, drugs, nutrients, family history, sexual history, travel history, contact with patients with jaundice, surgery, blood transfusion, liver disease history or allergic diseases and work environment.
- Physical examination and consideration of the possibility of liver metastases from the tumor.
- Liver imaging (eg, biliary tract).
- Laboratory tests: AST and ALT, alkaline phosphatase, GGT, albumin, total bilirubin, direct bilirubin, prothrombin time, factor V, CPK, lactate dehydrogenase, and troponin. Testing for hepatitis A, hepatitis B, hepatitis C and E, CMV, EBV, blood glucose, and triglycerides is also recommended.

Permanently discontinue once ALT > 3 × ULN is accompanied by total bilirubin > 2 × ULN, no hemolysis, alkaline phosphatase < 2 × ULN, or no testing. Once other abnormal laboratory values (including blood glucose and triglycerides) judged by the investigator to be clinically significant, dosing should be withheld until recovery to ≤ Grade 1 or baseline. If recovery to Grade 1 is complete or within 7 days, resume treatment at the same dose.

6.10.3 Gastrointestinal Events

Diarrhea has been reported to be a common adverse reaction to treatment with Trametinib with an incidence of greater than 20%, predominantly mild to moderate. Patients taking Trametinib may also experience colitis and gastrointestinal perforation, including death. In clinical trials with Trametinib (METRIC Study, NCT01245062), the incidence of colitis was 0.6% and the incidence of gastrointestinal perforation was 0.3% [28].

Preventive and control measures:

- If the patient experiences recurrent abdominal pain, diarrhea and other symptoms, the investigator should be contacted promptly, and relevant examinations should be performed to determine the etiology;
- If patients experience Grade 1-2 diarrhea, drugs that improve bowel function may be closely observed or used; in the event of Grade 3 diarrhea, symptomatic supportive drugs to improve intestinal function may be used, which generally recover within 3 days, and may be continued; if diarrhea has been treated for more than 3 days, the study drug needs to be discontinued;
- In the event of ≥ Grade 3 colitis or gastrointestinal perforation, hold until recovery to ≤ Grade 1 or baseline, and resume the same dose if resolved within 7 days. If recovery occurs between 7 and 21 days, down-titrate to the next dose group after recovery. Discontinue treatment if 2 dose modifications have been made or have not recovered within 21 days.

6.10.4 Decreased Left Ventricular Ejection Fraction

Cardiotoxicity, such as left ventricular ejection fraction, has been observed in early Trametinib trials in adult patients with melanoma [29]. Trametinib monotherapy has been reported to reduce left ventricular ejection fraction. In clinical trials of Trametinib monotherapy (METRIC study, NCT01245062), 11% of patients had a ≥ 10% decrease from baseline in left ventricular ejection fraction and 5% had a ≥ 20% decrease from baseline. In clinical trials, the mean time to first onset of left ventricular dysfunction, heart failure, and decline in left ventricular ejection fraction was 2-5 months [26-27, 30-31].

Cardiomyopathy, defined as left ventricular ejection fraction (LVEF) ≥ 10% below baseline, occurred in 23% of 74 pediatric patients who received selmetinib in the SPRINT study. One patient experienced a Grade 3 decrease in LVEF that led to a dose reduction. All patients with reduced LVEF were asymptomatic and confirmed by routine echocardiography. Left ventricular ejection fraction was decreased in 71% of patients [12].

Routine cardiac monitoring should be performed during treatment with the MEK inhibitor FCN-159. Patients should be monitored for cardiac symptoms such as murmurs, peripheral edema, and dyspnea. Once cardiac symptoms and signs are identified, cardiac echocardiography should be performed to further assess cardiac function [26, 32-34].

Symptoms of HF or radiographic changes from baseline should be managed according to the following guidelines:

No symptoms of HF and LVEF decreased to the lower limit of the normal range but ≥ 50%<br><br>Or < 10% absolute decrease in LVEF from baseline	Continue the same dose and repeat LVEF within a maximum of 4 weeks
Absolute decrease in LVEF ≥ 10% from baseline to the lower limit of the normal range without symptoms of HF.<br><br>Or symptomatic HF with 40% < LVEF < 50%	≤ 4 weeks off; if LVEF values improve to normal, recover at a lower dose; permanently discontinue if not improved to normal.
Symptomatic HF and LVEF < 40%<br><br>Or an absolute decrease in LVEF of ≥ 20% from baseline to the lower limit of the normal range.	Permanently discontinue the drug and follow-up the patient

6.10.5 Other cardiac events

ECG, echocardiography, or MUGA scan is mandatory once cardiac adverse events (e.g. ischemic heart disease) are suspected; troponin, NT-proBNP, and potassium may also be considered.

6.10.6 Proteinuria

According to data from the preclinical study of FCN-159, the kidney is one of the target organs of toxicity for FCN-159, manifested by tubular mineralization and renal function-related changes. In the SPRINT study, proteinuria was reported in 22% of pediatric patients treated with selmetinib, but no Grade 3 or higher proteinuria was observed [28].

Once proteinuria ≥ 2 + occurs, interrupt dosing and concurrently perform 24-hour urine protein quantification. Resume if proteinuria is ≤ Grade 1; if 24-hour urine protein quantitation ≥ Grade 2 or urine protein ≥ 2 +, continue withhold medication and actively administer treatment. Once proteinuria recovers to ≤ Grade 1, resume treatment at the same dose and continue to monitor urine protein. If it is interpreted as drug-related Grade 2 or higher

proteinuria lasting more than 1 treatment cycle, permanent discontinuation is recommended. If Grade 2 proteinuria recurs again, when proteinuria recovers to ≤ Grade 1, continue dosing at a reduced dose and pay attention to qualitative proteinuria testing. For Grade 3 proteinuria or other risk factors (eg, hematuria, hypertension), the investigator should consult a nephrologist.

6.10.7Acute hypertension

Hypertension of all grades was reported in 32 (15%) of 211 patients in the Trametinib treatment group and Grade 3 or higher in 26 (12%) patients in METRIC clinical studies receiving Trametinib [26].

Uncontrolled hypertension should be excluded from this study and, for patients with a history of hypertension, hypertension should be closely monitored and appropriately treated according to clinical practice. In the opinion of the investigator, in the event of an adverse event or co-occurrence of hypertension (e.g., proteinuria), a cardiologist and nephrologist should be consulted as soon as possible.

Table 4 Treatment of Hypertension

Grade 2 hypertension	Antihypertensive therapy should be actively administered and monitored. The investigator may continue to give the same dose of treatment or interrupt the drug according to the blood pressure condition, and continue after recovery to Grade 1.
Grade 3 hypertension	Antihypertensive treatment should be actively administered and monitored. The drug should be continued after recovery to Grade 1. If Grade 3 hypertension occurs again, the dose should be reduced after recovery to Grade 1.
Grade 4 hypertension	The drug was permanently discontinued and the patient was actively treated with antihypertensive therapy and closely followed up.

6.10.8 Pneumonia/Interstitial Pneumonia (ILD)

Patients treated with Trametinib monotherapy may develop ILD or pneumonia. In the Phase 3 study (METRIC Study, NCT01245062), 2.4% (5/211) of patients treated with Mekinist ® (Trametinib) monotherapy experienced ILD or pneumonia. The median time to first onset of ILD or pneumonia was 160 days [32].

Preventive and control measures:

- Patients with interstitial pneumonia, including clinically significant radiation pneumonitis, were excluded from the study;
- Ongoing physical examination including respiratory examination and vital signs monitoring during the study;
- Monitor the respiratory symptoms and signs of the patient. If chest tightness, tachypnea,

cyanosis, Velcro rales (continuous, high-pitched crackles) are heard in the middle and lower parts of both lungs, pulmonary function and pulmonary imaging should be performed promptly;

- Permanently discontinue treatment once Grade 2 interstitial pneumonia occurs.

6.10.9 Ocular toxicity

Clinical trials with the MEK inhibitor Trametinib have reported ocular events such as blurred vision, decreased vision, serous retinopathy, and chorioretinopathy. RVO occurred in 0.2% (4/1749) of clinical trials with Trametinib, and retinal pigment epithelial detachment (RPED) also occurred with Trametinib, but the true incidence is not known because Trametinib was not routinely monitored to monitor asymptomatic RPEDs in melanoma and NSCLC studies [26-27, 32, 35-37].

In the SPRINT clinical study, blurred vision, photophobia, cataracts, and ocular hypertension occurred in 15% of 74 pediatric patients treated with selumetinib. Blurred vision led to dose interruption in 2.7% of patients. Ocular toxicity disappeared in 82% of 11 patients [12].

No ocular toxicity was observed in the Phase 1 and preclinical studies of FCN-159.

Preventive and control measures:

- Patients with previous or current retinal vein stenosis, retinal detachment, central retinal vein occlusion, and glaucoma were excluded from the study;
- Avoid excessive use of eyes and pay attention to eye hygiene;
- Ophthalmologic examination: visual acuity, visual field, fundus examination under slit lamp, intraocular pressure, fundus photography/scanning laser fundus examination, ophthalmic B-ultrasound;
- Inform the patient that if eye discomfort or abnormality such as blurred vision occurs, the investigator should be contacted promptly, and an ophthalmologic examination is required by an ophthalmologist when ocular clinical symptoms occur;
- If Grade 2 or higher ocular toxicity occurs, such as retinal vein occlusion (RVO) and retinal pigment detachment (RPED), retinal detachment, permanent discontinuation is required.
- Retinal vein occlusion was reported and the patient-reported vision loss was assessed urgently (within 24 hours).

6.10.10 CK increased

Cardiac causes (combined with medical history, symptoms, signs and tests such as electrocardiogram, echocardiography, serum troponin, and CK-MB) and rhabdomyolysis (combined with physical activity, blunt trauma, or recent history of intramuscular injection, etc., symptoms, signs, and laboratory tests such as serum creatinine, potassium, calcium, phosphorus, uric acid, albumin, urinary myoglobin, etc.) were excluded. Consider permanent discontinuation of FCN-159 if there is evidence of clinically significant cardiac injury or rhabdomyolysis.

For asymptomatic Grade 1-2 CK elevations, if not considered clinically significant, continue FCN-159 at the current dose and cycle and repeat CK at regular intervals.

For asymptomatic Grade 3 CK elevations, if not considered clinically significant, continue FCN-159 at the current dose and cycle and recheck CK at least once weekly, and continue at the current dose and cycle if CK remains Grade 3 or downgraded.

For asymptomatic Grade 4 CK elevations, withhold FCN-159 and repeat CK within 3 days if not considered clinically significant. FCN-159 can be continued at a reduced dose level when CK elevation returns to ≤ Grade 3. If not resolved, continue withholding and monitor CK until recovery to ≤ Grade 3 and resume at a reduced dose level. If a Grade 4 CK increase reappears after 1 dose reduction, reduce the dose level again. Permanently discontinue FCN-159 if Grade 4 CK increase occurs again after 2 dose reductions.

7 Study Assessments

7.1Safety Assessments

Evaluate the frequency and severity of adverse events, serious adverse events, deaths and safety test abnormalities (e.g., laboratory tests, vital signs, physical examination, 12-lead electrocardiogram, ECOG, etc.) according to NCI-CTCAE; proportion of patients with dose modification or discontinuation due to drug toxicity.

7.1.1 Definition of Adverse Events

Adverse Event (AE): it refers to all untoward medical events that occur in a patient or a patient in a drug clinical study after receiving the investigational product. Symptoms, signs, diseases, or laboratory abnormalities may be present, but are not necessarily causally related to the investigational product. This includes any new or worsening in severity or frequency from baseline, including laboratory abnormalities.

This includes any new or worsening in severity or frequency from baseline, including laboratory abnormalities.

AEs do not include:

•Medical or surgical procedures should not be captured as AE terms (e.g., surgery, endoscopy, tooth extraction, infusion), whereas diseases leading to these procedures should be reported as AEs;

•Pre-existing diseases or conditions, including laboratory abnormalities, that were present or detected before the start of study drug but did not worsen;

Serious Adverse Event (SAE): a Serious Adverse Event is defined as the following untoward medical Event (at any dose):

•Leading to death

•Life-threatening

(Means that the patient was under threat of death at the time of the event.Excludes events that theoretically could have caused death if the adverse event was more severe)

•Requires hospitalization or prolongation of hospitalization

Note: in general, inpatient hospitalization refers to observation (usually at least overnight) in a hospital or emergency ward and/or treatment that is inappropriate in the physician's office or outpatient setting. Complications that occurred during hospitalization were adverse events (AEs). If a complication prolongs hospitalization or meets any other serious criteria, the event is serious. This AE should also be considered serious when it is uncertain whether "hospitalization" or "hospitalization" is required. Hospitalization for elective treatment or a pre-existing condition that has not worsened since baseline is not an AE.

•Resulting in permanent or significant disability/loss of function

Note: the term disability refers to a serious impairment of an individual's ability to exercise normal life functions. This definition does not include events of relatively minor clinical significance, such as simple headache, nausea, vomiting, diarrhea, influenza, and accidental trauma (e.g., sprained ankle) that may affect daily functioning but do not result in significant loss of function.

•Resulting in congenital malformations/birth defects in offspring

•Other medically significant events

Other medically significant events: in some cases, medical and scientific judgment must be used to decide whether to expedite reporting. For example, an important medical event may not be immediately life-threatening, result in death, or hospitalization, but if medical measures are required to prevent one of the above situations, it is usually considered serious.

Note: the following hospitalizations are not considered serious adverse events because there are no "adverse events" associated with hospitalization (i.e., no adverse medical events):

- Admission to temporary care;
- Hospitalization for social reasons, e.g. admission for convenience of care;
- Planned hospitalizations required by the protocol, e.g., for study drug administration or for insertion of study drug or protocol-required laboratory tests;
- Hospitalization, elective surgery, or examination of a pre-existing condition planned prior to informed consent (in which case, the condition requiring hospitalization does not worsen or progress to a new disease after administration of the study drug and is documented in the archiving of the original document);
- Hospitalization for routine maintenance (e.g. battery replacement) of equipment in place prior to participation in the study.

Adverse Drug Reaction (ADR): it refers to any Adverse or undesired Reaction that may be related to the investigational Drug in a clinical trial. There is at least one reasonable possibility that the causal relationship between the investigational product and the adverse event cannot be ruled out.

7.1.2 Collection and Recording of Adverse Events

Start point of AE/SAE collection records: from signing the ICF.

Note: clinical adverse events occurring after signing the ICF until before the first dose are recorded in the CRF as medical history/concomitant diseases and will not be recorded as AEs unless one of the following is met:

- Any adverse event related to procedures specified in the clinical study protocol (e.g., exercise testing, laboratory tests, etc.);
- Adverse events associated with discontinuation of treatment related to the protocol, such as changes or discontinuations of prior/concomitant therapies;
- Adverse events caused by a drug other than the investigational product taken as part of the treatment regimen.

Endpoints recorded for AE/SAE collection: 30 days after the last dose of study drug or initiation of other anti-tumor therapy by the patient, whichever occurs first.

Note: aEs/SAEs that are related to the study drug after the AE/SAE collection and recording endpoints should also be collected and reported.

Abnormal laboratory findings (e.g., blood chemistry or hematology) or other abnormal assessments (e.g., ECG or vital signs) that are known to be not concomitant with the disease

under study are judged by the investigator to be clinically significant and must be recorded as AEs if they meet the definition of AEs or SAEs if serious criteria are met. During the trial, patients are advised to report any adverse events, and trained staff should ask patients regularly for any adverse events in a non-inductive manner. All adverse events directly observed by the investigator or reported by the patient were reported in concise language.

It is the responsibility of the investigator to examine all laboratory results for all patients and to determine whether they constitute adverse events. Medical and scientific judgment should be exercised in deciding whether separate laboratory abnormalities should be classified as adverse events.

Adverse events should be truthfully reported during the study, including the name, onset and end date, severity, whether the AE is a serious adverse reaction, the investigator's causal relationship between the study drug and the event, the action taken and the outcome of the adverse event, etc., and the relationship to the study drug should be evaluated on the basis of comprehensive consideration of comorbidities and concomitant medications. All adverse events occurring during the study should be recorded in the CRF.

In addition, SAEs will collect the following additional data elements: date of AE escalation to SAE, date of investigator becoming aware of AE escalation to SAE, cause of AE as SAE, date of hospitalization and discharge, possible cause of death, date of death, necropsy findings, assessment of causal relationship of SAE to study procedures, event description of AE, etc.

7.1.3 Assessment of Adverse Events

7.1.3.1Assessment of Severity of Adverse Events

The severity of adverse events was assessed using the NCI-CT CA E 5.0 Adverse Event Severity Grading Scale.

Table 5 Adverse Event Severity Grading Scale

Grade	Severity
Grade 1	Mild; asymptomatic or mild; clinical or diagnostic findings only; no treatment required.
Grade 2	Moderate; requires small, local, or noninvasive treatment; age-appropriate instrumental activities of daily living are limited *.
Grade 3	Serious or medically significant but not immediately life-threatening; leading to hospitalization or prolongation of stay<br>Hospital time; disabling; self-care activities of daily living are limited * *.
Grade 4	Life-threatening; urgent treatment is required.
Grade 5	Deaths related to AEs.

* Instrumental activities of daily living refer to cooking, shopping for clothes, using the telephone, managing money, etc.

* * Self-care activities of daily living refer to bathing, dressing and undressing, eating, washing, taking medicine, etc., and are not bedridden.

7.1.3.2 Assessment of Relationship between Adverse Events and Study Drug

The investigator should assess whether the adverse event is related to the study drug based on his/her knowledge of the patient and the background of the event, as well as any potential possible cause. The causal relationship of the AE to the study drug is as follows:

- Definitely related: there is a reasonable temporal relationship between drug use and the occurrence of adverse events; the reaction disappeared or rapidly alleviated and improved after discontinuation of the drug (i.e., positive dechallenge); adverse events of rechallenge reappear (i.e., positive rechallenge) and may worsen significantly; supported by the Investigator ' s Brochure or literature data at the same time; other confounding factors such as the underlying disease have been excluded.
- Probably related: there is no history of repeated medication, the rest is "definitely related", or although there are concomitant medications, the possibility of adverse reactions caused by concomitant medications can be basically ruled out.
- Possibly related: the drug has a close relationship with the onset time of the adverse event, which is supported by literature data; however, there is more than one drug that causes adverse events, or the progression of the underlying disease cannot be ruled out.
- Unlikely related: the adverse event is not closely related to the time of administration, the clinical manifestations are not consistent with the known adverse reactions of the drug, and the development of the underlying disease may have similar clinical manifestations.
- Not related: no medication was administered, or there was no correlation between the medication and the time of onset of the adverse event, or there was another clear cause of the adverse event.

Definitely related, probably related and possibly related are listed as adverse drug reactions.

7.1.3.3 Assessment of expectedness of adverse events

Unexpected adverse reactions refer to the nature, severity, consequences, or frequency of adverse reactions that are different from the expected risks described in the current relevant

information of the investigational product (e.g., Investigator's Brochure, etc.). The Investigator ' s Brochure serves as the primary document to provide reference safety information to determine whether an adverse reaction is expected or unexpected.

Suspected Unexpected Serious Adverse Reaction (SUSAR): it refers to a Suspected and Unexpected Serious Adverse Reaction in which the nature and severity of clinical manifestations exceed the available information such as the Investigator's Brochure of the investigational product, the package insert of a marketed drug, or a summary of product characteristics.

7.1.4 Serious Adverse Event Reporting

The investigator should actively take appropriate measures to ensure the safety of any SAE during the trial, whether related to study drug or not. The investigator should report the SAE to the sponsor within 24 hours of becoming aware of the SAE (e-mail fsadedesk @ fosunpharma.com).

For reports involving death events, the investigator should provide the sponsor and the Ethics Committee with other required information, such as an autopsy report and a final medical report.

The investigator should sign and read the relevant safety information of the clinical trial provided by the sponsor in a timely manner, consider whether the patient's treatment is adjusted accordingly, communicate with the patient as soon as necessary, and report suspected and unexpected serious adverse reactions provided by the sponsor to the Ethics Committee.

The investigator should follow up the SAEs as required by the protocol, and provide a detailed and written follow-up report within 24 hours of being informed of the follow-up information in the same manner as above.

The sponsor or its representative shall evaluate and report SAEs (including SUSARs) in clinical trials in accordance with the latest applicable regulatory requirements for drug clinical trials.

7.1.5 Follow-up of Adverse Events

The investigator should follow up each adverse event, and all SAEs and related AEs that are ongoing at the time of AE/SAE collection and recording endpoints should be followed up until:

- Resolution or return to baseline status or stabilization of the event;
- The investigator determined that there would be no further improvement;

- When more information is not possible (the patient refuses to provide further information, or there is evidence that the patient is still lost to follow-up after doing his best efforts).

The recovery time of adverse events (dated) should be recorded in the Adverse Event eCRF and patient ' s medical records during the study to verify the raw data.

For serious adverse events, adverse events of special interest, and pregnancy events, the sponsor or other designee may obtain additional case information by telephone, fax, email, and/or monitoring for independent medical assessment of these reported cases.

The updated AE raw data after database lock should be recorded for backup. The SAE information should also be filled in the Serious Adverse Event Report Form as detailed as possible and submitted to PV Department.

7.1.6 Outcome of Adverse Events

The investigator should determine the outcome of the adverse event based on the outcome of the patient ' s adverse event. The outcome of an adverse event has the following:

- Recovered: the patient fully recovered from the AE without any residual effects or harms.
- Recovering/resolving: signs and symptoms associated with the event have decreased but not completely resolved.
- Recovered with sequelae: the patient has recovered but is still associated with residual effects or injuries. These residual effects may be temporary but still present at the time of reporting. If sequelae are not considered permanent, additional information is required at follow-up when the event changes.
- Unaltered: the signs and symptoms associated with the event did not diminish and the patient ' s condition remained unchanged.
- Disease worsening: the event-related signs and symptoms did not decrease and the patient ' s condition worsened.
- Death: only SAEs leading to death can select "death" as the outcome. All other AEs/SAEs present at the time of death should be reported.
- Unknown: when the patient was lost to follow-up, the investigator was unable to determine the outcome.

7.1.7 Pregnancy Reporting

For patient safety reasons, if A female patient or A female partner of A male patient becomes pregnant while on study drug treatment, pregnancy report form A needs to be

completed to the sponsor within 24 hours of awareness, and the pregnancy must be followed up to clarify the outcome of the pregnancy (if the female partner of the male patient agrees to collect information), including spontaneous or induced abortion, birth details, congenital malformations, or maternal or neonatal comorbidities, etc., and pregnancy report form B must be completed for reporting.

Reports of spontaneous abortion, ectopic pregnancy, induced abortion due to medical and health reasons, stillbirths, and SAEs of newborns (not limited to neonatal death, all congenital malformations/birth defects) are considered serious adverse events (SAEs), and SAEs should be reported along with the completion and reporting of the pregnancy report form. Selective abortion without complications is not considered an adverse event. If other SAEs occur during pregnancy, they must also be reported.

7.1.8 Overdose

overdose in this trial is defined as follows: the total amount of drug taken per day or the number of days used exceeds the protocol.

If a subject experiences symptoms due to an overdose, the overdose and all associated symptoms should be recorded in the eCRF and reported to the sponsor within 24 hours of awareness according to the SAE procedure. Simple overdose events (no associated AEs or SAEs) do not need to be reported as SAE procedures, but overdose information needs to be recorded in the eCRF.

7.1.9 Disease progression

Progressive disease is defined as worsening of a patient ' s condition due to disease treated with study drug. Disease progression may be an increase in severity and/or worsening of disease symptoms of the disease under study. Enlargement of the primary tumor or metastasis under study meeting the PRC Response Criteria/RECIST v1.1 criteria for progression, or the appearance of new metastases, should be considered disease progression rather than AEs or SAEs. Events determined to be due to disease progression during the study should not be reported as AEs or SAEs.

For deaths due to unequivocal disease progression, the date of death and cause of death should be recorded in the eCRF and should not be reported as SAEs.

7.1.10 New Cancer

Emerging cancer should be considered an SAE. A new primary cancer is one that is not the primary reason for study treatment and is found after the patient is enrolled in the study,

and the investigator should provide appropriate imaging or pathological examination as the basis for the new cancer.

7.2 Efficacy Assessment

7.2.1PRC Criteria

Histiocytic infiltration in histiocytic tumors is usually multifocal and may involve bone, soft tissue, and central nervous system (CNS) structures. Since the lesion consists of histiocytic, inflammatory interstitial, and mixed fibrosis heterogeneity, only the measured tumor size may not reflect a favorable treatment response. In contrast, serial metabolic assessments of 18F-FDG PET/CT have been found to be the most reliable radiographic marker of response to treatment in these diseases. PERCIST criteria are a system based on FDG-PET metabolism to assess treatment response. However, since SUVmax is more commonly used in the clinic than SULpeak, the modified PET Response Assessment Criteria (PRC) based on the PERCIST criteria will be used in this study to measure metabolic response in histiocytic tumors, replacing SULpeak with SUVmax in the PERCIST criteria. The PRC criteria are similar to the RECIST method, with up to 5 target lesions selected based on reproducibility in the FDG affinity assessment to reflect the involvement of no more than 2 per organ. Target lesions should meet the following requirements: the sum of SUVmax > 1.5 times the mean standard uptake value of liver (SUVmean) and 2 times the standard deviation of hepatic SUVmean for non-intracranial/skull lesions. If liver lesions are involved, the sum of SUVmax > 2 times the mean standard uptake value of mediastinal blood pool (SUVmean) and 2 times the standard deviation of SUVmean in the mediastinal blood pool should be used as the standard deviation. The sum of intracranial/skull lesion SUVmax > 1.5 times the mean standard uptake value in white matter (SUVmean) and 2 times the standard deviation of SUVmean in white matter.

The 18F-FDG uptake value (SUV) for each non-intracranial/skull target lesion is calculated as target lesion SUVmax – hepatic SUVmax (if liver lesions are involved, target lesion SUVmax – mediastinal blood pool level SUVmax) and intracranial/skull target lesion SUV = target lesion SUVmax – brain white matter SUVmax. If the result is negative, it is defined as 0. The PRC response criteria are shown in the following table:

Table 6Definition of PET Response Assessment Criteria (PRC)

Response Category	Criteria based on sum of SUVs of up to 5 target lesions
Complete Metabolic Response (CMR)	Normalization of SUVmax to background or below background for all lesions (target and non-target lesions) (SUVmax for non-intracranial/skull lesions liver/mediastinal blood pool, SUVmax for intracranial/skull lesions in white matter)
Partial Metabolic Response (PMR)	≥ 50% decrease from baseline in sum of SUV for all target lesions

Progressive Metabolic Disease (PMD)	≥ 50% increase in the sum of SUV from nadir for all target lesions, with an absolute increase in SUV of at least 3 units per target lesion (e.g., SUV 3 to SUV 6); or a new evaluable lesion considered to represent unequivocal disease progression
Stable Metabolic Disease (SMD)	Other criteria not met

The response to treatment will be assessed by the IRC and the investigator according to the PET response assessment criteria and RECIST v1.1 criteria, respectively. 18FDG-PET/CT scans ranged from the skull top to the plantar. Tumor assessments may be increased by the investigator as clinically indicated, and the investigator should make subsequent treatment decisions based on his/her response assessment results. Tumor assessments may be used as baseline tumor assessments if they have been performed within 28 days prior to the first dose and the same method and machine are used in the same hospital. Tumor assessments will be performed once at screening, at the end of treatment cycles 3, 6, 12 (± 7 days), and at the end of treatment. Patients evaluated as CMR or PMR had confirmed response by repeated assessments at least 4 weeks later. Tumor assessments per PRC criteria will be performed every 3-6 treatment cycles (± 14 days) if, in the opinion of the investigator, the imaging results are not consistently stable after 12 treatment cycles. If a patient discontinues treatment, tumor assessments should continue as scheduled until disease progression, death, withdrawal of informed consent, loss to follow-up, start of new anti-tumor therapy, or end of the study. At the End of Treatment Visit, if tumor imaging has been performed in the first 8 weeks, tumor imaging may not be repeated at the End of Treatment Visit.

7.2.2RECIST Criteria

The RECIST-based assessment criteria are presented in Attachment 7. Patients were assessed for response to treatment by IRC and investigator per RECIST v1.1 assessment criteria. MRI/CT includes MRI/CT of the head, neck, chest, abdomen, and pelvis. Target lesions are suggested by PET-CT or new lesions during treatment are selected or added, but the same imaging assessment method (MRI/CT) should be used for each patient throughout the treatment.

Tumor imaging will be assessed by the investigator and the IRC according to RECIST version 1.1, respectively. Tumor assessments may be increased by the investigator as clinically indicated. The investigator should make subsequent treatment decisions based on their own response evaluation results. Tumor assessments may be used as baseline tumor assessments if they have been performed within 28 days prior to the first dose and the same method and machine are used in the same hospital. Tumor assessments will be performed once at screening, at the end of treatment cycles 3, 6, 9, 12 (± 7 days), and at the end of treatment. Tumor

assessments will be performed according to RECIST 1.1 every 4 treatment cycles (± 14 days) if the imaging results are considered to be consistently stable by the Principal Investigator after 12 treatment cycles, and every 3 treatment cycles (± 7 days) if the imaging results do not reach sustained stabilization in the opinion of the investigator after 12 treatment cycles. If a patient discontinues treatment, tumor assessments should continue as scheduled until disease progression, death, withdrawal of informed consent, loss to follow-up, start of new anti-tumor therapy, or end of the study. At the End of Treatment Visit, if tumor imaging has been performed in the first 4 weeks, tumor imaging may not be repeated at the End of Treatment Visit.

7.2.3 Definition of Tumor Assessment Endpoints

Objective response rate (ORR) is defined as the proportion of patients who achieved confirmed CMR and PMR based on the PRC response evaluation criteria, respectively, and the proportion of patients who achieved confirmed CR and PR as assessed by RECIST v1.1 response evaluation criteria, respectively.

DCR is defined as the proportion of patients who achieved confirmed CMR + PMR + SMD based on the PRC response evaluation criteria and the proportion of patients who achieved confirmed CR + PR + SD according to RECIST v1.1 response evaluation criteria, respectively.

Time to Response (TTR) is defined as the time to first CMR or PMR (whichever is recorded first) based on the PRC Response Evaluation Criteria and the time to first CR or PR (whichever is recorded first) based on RECIST v1.1 Response Evaluation Criteria, respectively.

PFS rate is defined as the proportion of patients who have not progressed (PD/PMD) or died, whichever occurs first, after enrollment.

OS is defined as the proportion of patients who survived after the first dose of study drug. Patients without events were censored at the last date known to be alive.

CBR is defined as the proportion of patients with confirmed CMR + PMR + SMD per PRC criteria and confirmed CR + PR + SD lasting ≥ 24 weeks as assessed by RECIST v1.1 Response Evaluation Criteria.

7.2.4Independent Imaging Assessments

The blinded IRC will conduct a retrospective review of radiographic images and clinical information collected during the study to validate protocol-defined endpoints such as investigator-assessed disease response and progression. See IRC Charter for details.

7.3Patient-Reported Outcomes

Assessed using the EORTC-QLQ-C30 scale (V3.0), see Attachment 3.

7.4 Pharmacokinetic Evaluation

7.4.1 Blood Sample Collection

To evaluate the population pharmacokinetic profile of FCN-159, plasma samples were collected at the following time points, the concentrations of FCN-159 in plasma were measured, and the corresponding population pharmacokinetic parameters were calculated.

C1D1: pre-dose

C2D1: pre-dose; 0.5-1 hour, 1.5-3 hours, 4-6 hours after dosing;

C4D1, C7D1, C10D1: pre-dose.

Refer to a separate laboratory manual for specific procedures, storage conditions, and shipping instructions for blood sample collection at each blood collection time point. Plasma concentrations of FCN-159 will be measured by a designated project central laboratory. Results are presented as bioanalytical reports.

7.4.2 Blood Sample Processing and Transportation

The blood samples taken will be anticoagulated, centrifuged, plasma will be separated, aliquoted, stored in a cryogenic or ultra-deep freezer below -20 °C for testing. (Biological sample processing shall be performed in accordance with the Operating Manual of the Project Central Laboratory). Blood sample shipment: samples will be shipped in dry ice packages that can be maintained for 3 days and shipped in cold chain from the sampling points to the project central laboratory in duplicate.

8 Data Management

This study is based on the requirements in the Technical Guidelines for Clinical Trial Data Management promulgated by NMPA in 2016, the Guidelines for Planning and Reporting of Data Management and Statistical Analysis of Drug Clinical Trials, and Technical Guidelines for Electronic Data Collection of Clinical Trials. The main items are described below:

8.1 Data Management System

An electronic data capture system (EDC) will be used for this study. The EDC system is a computerized data management system including data transmission, database creation, data entry, data quality verification and data reporting. Its main working principle is direct electronic data acquisition (EDC) via the Internet.

8.2 Data Management Quality Control

Data management personnel will develop a Data Management Plan to standardize the data

management process and technical standards for the entire study, and to ensure the authenticity, completeness, accuracy, timeliness and traceability of the clinical trial data. At the end of the study, the actual situation of data management will be summarized according to the specific completion of the study and in accordance with the contents and procedures specified in the Data Management Plan, and various records and supporting materials will be summarized.

8.3 Structure and Specification of Database

The database access rights of the EDC users are set according to the study project work function role, and all operations of each user are traced to ensure that every modification to the data and queries by all users is fully documented. The database designers will archive the database design documents in accordance with regulatory requirements.

8.4Database Testing and Review

The data manager will establish an eCRF according to the protocol, including: page design, visit period setting, order of entry forms at visits, and the order of each data point; accuracy of browsing permissions for different users, etc.

8.5 Data Completion Requirements

The investigator or authorized personnel are responsible for completing the eCRF, and the items in the eCRF should be carefully and detailedly recorded, and no empty items or missing items should be allowed (UK/NA/ND should be filled in if there is no recorded space); all data in the eCRF must be reconciled with the data of the patient's original data to ensure accuracy.

The investigator must keep all original documents; for abnormal laboratory or test data, the investigator should verify and indicate whether it is clinically significant; the investigator should strictly follow the eCRF completion guidelines for data completion; for data correction in the eCRF, the reason for the data modification should be filled in according to the system requirements.

8.6 Query Generation and Answer

The EDC system will automatically calibrate the entered data according to the data verification plan. The problems found are questioned in the system. Data management personnel will also manually generate queries in the EDC according to manual verification procedures. The monitor and medical monitor will also manually generate queries based on the data. The investigator should promptly respond to queries from the system and data reviewers.

The data manager, monitor and medical monitor review the investigator ’ s answers to queries, and may issue them again if necessary until the data is "clean".

8.7Medical Review of Data

The medical monitor will conduct medical review of the study data, focusing on the study data related to the study inclusion/exclusion criteria, the rationality of clinical significance determination of clinical trial test parameters, recording and reporting of adverse events and serious adverse events, medical history information, prior and study concomitant medication information, study drug-related information, and protocol deviations, etc.

8.8 Data Locking

When the following conditions are met (including but not limited to), the database is locked according to the requirements of database lock.

- All data have been entered into the database;
- All queries have been resolved;
- The statistical analysis population has been identified and judged.

The locked data files will not be changed without authorization from the sponsor. The specific data management process will be described in detail in the Data Management Plan.

9 STATISTICAL AND STATISTICAL ANALYSIS

The statistical analysis plan should be finalized after the protocol is finalized and finalized prior to database lock. All planned statistical analyses will be specified and described in detail in the statistical analysis plan according to the main characteristics of the protocol. Descriptive statistical analysis is predominant in this study. All statistical analyses were calculated by the Sponsor ' s Clinical Data Analysis Department using SAS statistical analysis software.

All variables obtained at each observation time point were statistically described in this study, unless otherwise specified. In general, continuous variables (e.g. age) will be statistically described using the number of observations, mean, median, standard deviation, minimum, and maximum; categorical variables will be statistically described using frequencies and their percentages for each category. Statistical methods will be detailed in the statistical plan.

9.1 Sample Size Calculation

This study is a single-arm clinical study of rare diseases, and it is planned to enroll approximately 25 evaluable patients, considering a dropout rate of 10%, and a total of approximately 28 patients will be enrolled. Assuming that the proportion of patients achieving CMR and PMR is 80%, the following table presents the ORR and 95% confidence interval corresponding to the different number of responders.

Relationship between ORR, 95% CI and number of responders in 25 evaluable patients

Number of responders	18	19	20	21	22

Estimated ORR	72%	76%	80%	84%	88%
95% confidence interval (%)	(50.6, 87.9)	(54.9, 90.6)	(59.3,93.2)	(63.9, 95.5)	(68.8,97.5)
95% Confidence Interval Width (%)	37.3	35.7	33.9	31.6	28.7

9.2Analysis Set

- Intent-to-Treat (ITT): patients who signed informed consent and took at least one dose of FCN-159.
- Modified Intent-to-Treat (mITT): patients who took at least one dose of FCN-159, had baseline tumor assessment data and at least one post-baseline tumor assessment data, and had no major protocol violations.
- Safety Analysis Set: any patient who took at least one dose of FCN-159 and had at least 1 safety assessment.
- PK Analysis Set: includes patients who took at least one dose of FCN-159 and had at least 1 PK blood sample collected as planned and had plasma concentration data of study drug.

9.3Safety Analyses

Safety will be evaluated by summaries of AEs, laboratory tests (including hematology, blood chemistry, urinalysis, etc.), 12-lead ECG, ophthalmology, vital signs, physical examination, ECOG, etc. AEs will be summarized separately during the first cycle of the continuous monotherapy period and throughout the treatment period, and treatment-related AEs, SAEs, AEs with toxicity grade ≥ 3, and AEs leading to discontinuation will be summarized.

A TEAE is defined as an adverse event that occurs from the first dose of study drug to 30 days after the last dose, and an AE/SAE judged to be related to the investigational drug after 30 days after the last dose. Adverse events will be coded using MedDRA and graded using NCI-CT CA E version 5.0.

Laboratory changes will be summarized. For laboratory variables, maximum toxicity occurring during the study will be summarized by count and percentage. Descriptive statistics will be performed for changes from baseline for vital signs, physical examination, 12-lead ECG, and ECOG score.

9.4 Efficacy Analysis

Descriptive statistical methods were mainly used for the test results. Measured data generally need to be presented: number of observations, mean, standard deviation, median,

quartile, maximum and minimum. Counting data are presented for frequencies and frequencies (constituent ratios). Unless otherwise specified, two-sided tests with 95% confidence intervals were used for all statistical tests. Statistical analysis will be performed using SAS version 9.4 and above.

Descriptive statistics were used to describe the number and proportion of patients enrolled in each analysis set, the number and proportion of patients who completed and withdrew from the trial, and the reason for withdrawal (and the proportion of participants); and descriptive demographic and other baseline characteristics.

Confirmed ORR (CMR + PMR/CR + PR) will be evaluated by PRC and RECIST 1.1, respectively. Based on the efficacy evaluation results of each cycle, the final efficacy evaluation results of each patient will be determined, ORR will be calculated and its 95% confidence interval will be calculated using the Clopper-Pearson method. Descriptive statistics were performed for DCR and CBR. At the same time, survival analysis summaries (including time, status, cumulative survival and standard error, cumulative events and remainder; and mean and median survival time with standard error and 95% confidence interval) will be performed using the Kaplan-Meier method based on investigator assessment.

9.5 Pharmacokinetic Analyses

FCN-159 plasma concentration data obtained in this study and data from previous studies will be included in the population PK analysis. The population PK model will be described in a separate population PK analysis plan and a separate summary report will be provided and will not be included in the clinical report for this study.

9.6 Analysis of Clinical Outcomes

Observed values as well as changes from baseline will be summarized using descriptive statistics by the global health status/quality of life, function, and symptom scales of the EORTC-QLQ-C30 and individual item scores.

9.7 Interim Analyses

There were no interim analyses in this study.

10 Study Management

10.1 Ethical Considerations

This study will be conducted in compliance with the ethical requirements of the Declaration of Helsinki (Fortaleza 2013 Edition), the provisions of the International

Conference on Harmonization (ICH) E6 Good Clinical Practice, the national laws and regulations related to clinical studies, and the conduct of this protocol.

The protocol, informed consent form, medical record report form and other materials must be submitted to the Ethics Committee for review and approval before the start of the study. The Ethics Committee will review and approve these materials in strict compliance with the requirements of relevant laws and regulations. The study may not commence until the approval of the Ethics Committee has been received.

Any modifications made to the protocol during the course of the study must also be reviewed and approved by the Ethics Committee before they can be implemented specifically.

In addition, the Ethics Committee will approve all clinical trial protocol amendments (except administrative changes approved by the sponsor), informed consent forms and updates, patient recruitment procedures, written information provided to patients, available safety information, information related to remuneration and subsidies available to patients, curricula vitae and/or other certificates of eligibility of investigators, and any other documents required by the Ethics Committee and regulatory authorities, as applicable.

10.2 Informed Consent

The investigator or his/her designated representative will be responsible for explaining the study background, the pharmacological characteristics of the study medication, the protocol, and the benefits and risks of participating in the study to each patient, the patient ’ s legally acceptable representative or independent witness. Patients must be given sufficient time and opportunity to inquire about the details of the trial, to have their questions answered satisfactorily, and decide whether to participate in the study. Written informed consent will be obtained from the patient or his/her legal representative and the investigator or his/her representative before entering the study (prior to the screening examination).

The final informed consent form text should contain the following: study background, study objectives, study process, patient cooperation matters, risks and discomfort of participating in the study, possible damage caused by trial-related procedures, preservation and handling of biological samples, benefits of participating in the study, alternative treatment, and costs related to participation in the study; treatment and appropriate insurance compensation available to the patient in the event of study-related injury; access to study data and confidentiality of patient information, etc. This informed consent form should be written in non-technical language and approved by the Institutional Review Board/Independent Ethics Committee.

The informed consent form must be signed and dated by the patient or his/her legal representative, the investigator performing the informed consent process, or his/her representative. One copy of the informed consent form should be retained by the investigator and the patient. If important new data related to the study drug are found, or new information that will affect the willingness of the patient to continue to participate in the study, the patient or his/her legal representative should be informed promptly, and the patient's informed consent should be obtained again.

10.3 Protocol Amendments

Any significant amendments to this protocol should be submitted to the Ethics Committee for approval by a written amendment with the consent of the sponsor and the investigator before implementation, and submitted to regulatory authorities for filing.

Any changes to the protocol will require written protocol amendments and must be approved by the sponsor before administrative changes are implemented. If the change has a special impact on patient safety, the scope of the study, or the scientific quality of the study, an application needs to be submitted to the regulatory authorities and approval should be obtained from the appropriate ethics committee of each test facility. These requirements shall not preclude immediate action by the investigator or sponsor to protect the safety and interests of all patients. If, in the opinion of the investigator, immediate changes or deviations from the protocol are required for safety reasons to exclude harm to the patient, the sponsor ’ s medical monitor and the Ethics Committee of the test facility should be notified immediately. The sponsor must notify the regulatory authorities in accordance with local regulations.

Protocol amendments involving only administrative or administrative aspects of the study need not be submitted to regulatory authorities or ethics committees, but should be notified to regulatory authorities or ethics committees in accordance with local regulations.

10.4 Protocol deviations

The investigator must not deviate from the protocol if no formal amendment to the clinical trial protocol has been identified and approved by the appropriate ethics committee, unless it is to eliminate a direct hazard to the patient or when the change involves only the administrative or administrative aspects of the study and is approved by the medical monitor and/or the sponsor.

All requirements specified in the protocol must be strictly followed. Any intentional or unintentional deviation or violation of the protocol and GCP principles can be classified as a deviation from the protocol or a violation of the protocol. The investigator or investigator ’ s

designee should record and explain the details and reasons for the protocol deviation/violation and inform the regulatory authorities or ethics committee in accordance with local regulations.

Specific protocol deviations or protocol violations are described in the Medical Monitoring Plan.

10.5Patient Confidentiality and Privacy

The patient/patient ’ s legal representative agrees that the patient ’ s information is collected and processed by signing the informed consent form, and the patient ’ s personal information and privacy will be kept strictly confidential, and the collection and processing will comply with the Personal Information Protection Law of the People ’ s Republic of China and other relevant regulations. Personal information collection and processing of patients in this study will be limited to those data (e.g., copies of identity cards, bank card numbers, medical health information, etc.) necessary to investigate the efficacy, safety, tolerability, quality and utility of the study drug. Excessive collection of personal information is prohibited, and only investigators, monitors, other authorized representatives of the sponsor, ethics committees, and representatives of regulatory authorities will be allowed direct access to the patient ’ s personal information. During the study, each patient will have a unique study identification number, and personal information will not be transferred to third-party institutions (e.g., contract research organization CROs, bioanalytical testing, clinical testing, clinical systems, SMO, warehousing/cold chain/logistics, etc.) until personal information is de-identified, and it is strictly prohibited for anyone to provide patient information directly to other individuals or organizations by any means. The results of the study may be published in journals but will not disclose the patient ’ s personal identifiable information.

10.6 Monitoring

The investigator should allow the clinical monitor to inspect outpatient, laboratory, and pharmacy facilities and access the medical record report form, informed consent form, and all raw materials to ensure that the study complies with Good Clinical Practice and local regulatory requirements.

The designated clinical monitor will conduct monitoring visits to each test facility in accordance with the monitoring plan. On-site visits will be conducted prior to the start of the study. Periodic visits are required during the conduct of the study. Contacts may be made by telephone, fax, or email as needed as a supplement to on-site visits.

The investigator will be notified of the expected frequency of monitoring visits prior to the start of this study. In addition, during the course of the study, the investigator will be

notified in advance of each monitoring visit. The purpose of the visit is to ensure strict adherence to the protocol for the conduct of the clinical study; the completeness and accuracy of the medical record report form can be confirmed from the original documents.

The clinical monitor shall verify that all medical record report forms are correctly completed and complete and consistent with the original data; all errors or omissions have been corrected or noted, signed and dated by the investigator. At each visit, close cooperation between the Investigator and the Clinical Monitor is required to review and confirm the Medical Record Report Form, Drug Supply and Inventory Records, Drug Dispensing and Recovery Recycling Records, and any other scheduled additional records.

10.7 Quality Assurance and Quality Control

The sponsor, investigator and contract research organization should perform their respective responsibilities in accordance with the requirements of GCP, strictly follow the study protocol and adopt corresponding standard operating procedures to ensure the quality control and implementation of quality assurance systems in clinical trials, sample testing, data statistical calculation, etc.

In order to ensure the quality of the study, all investigators must be trained in the protocol before the start of the study, and SOPs should be strictly implemented during the study. The sponsor should send qualified monitors to supervise the study process and check the study data.

The sponsor may entrust auditors to conduct systematic audits of trial-related activities and documents to review whether the study is conducted in accordance with the requirements of the protocol, standard operating procedures, and relevant regulations and technical guidelines, and whether the study data are recorded and reported in a timely, true, accurate and complete manner. Auditors should be performed by personnel independent of the clinical trial.

Relevant data and documents (including medical records) of study sites and laboratories participating in clinical trials should be inspected and verified by drug regulatory authorities.

10.8 Direct Access

The Principal Investigator will provide direct access to source data and documents to Ethics Committee personnel, monitors, and other designees of the sponsor who conduct study-related monitoring and/or review. The purpose of monitoring or auditing is to systematically and independently inspect all study-related activities and documentation, determine whether these activities have been performed, and to record, analyze, and accurately report data in accordance with the protocol, GCP, ICH guidelines, and any appropriate regulatory

requirements. The Principal Investigator will promptly inform the Sponsor if the Agency and/or Principal Investigator is contacted by a regulatory authority for verification.

The investigator must inform the patient that his/her study-related records can be reviewed by the above-mentioned individuals without violating the privacy of the patient's personal health information.

10.9 Data Recording and Storage

In order to ensure the evaluation and supervision of regulatory authorities and the sponsor, the investigator should agree to maintain all study data, including confirmation records for all patients (which can effectively reconcile all recorded data, such as medical record report forms and hospital original records), all original signed patient informed consent forms, all medical record report forms, detailed records of drug dispensing, etc. The retention period is 5 years after the study drug is approved for marketing or until the time limit agreed upon in the clinical contract and notification of destruction by the sponsor is obtained.

All data of this clinical study are the property of the sponsor. Except as required by regulatory authorities, the investigator may not provide it to third parties in any form without the written consent of the sponsor.

10.10 Insurance and Patient Compensation

The sponsor will provide insurance related to the clinical trial in accordance with all applicable laws and regulations.

The sponsor will bear the cost of treatment and corresponding financial compensation for any injury caused by the patient's participation in this study that is causally related to the study, except for those caused by medical malpractice.

10.11 Storage and Use of Biological Specimens

Biological samples for this study will be stored in the central laboratory of the designated test samples for use in this clinical study only. The test blood samples will be destroyed after the completion of the testing, and the backup blood samples will be stored until two years after the study drug is approved for marketing.

10.12Study Interruption and Early Termination

The sponsor reserves the right to discontinue the study at any time for medical reasons or for any other reason. If the study is prematurely discontinued or interrupted, the sponsor should promptly notify the investigator that the study has been discontinued or interrupted, and the reason for discontinuation or interruption should be stated. In accordance with the requirements

of relevant regulations, the sponsor or investigator should also promptly notify the Ethics Committee that the study has been terminated or interrupted, with reasons.

The investigator reserves his/her right to determine whether the study should be discontinued. If the investigator terminates or interrupts the study without prior consent from the sponsor, the investigator should promptly notify the sponsor and the Ethics Committee and provide the sponsor and the Ethics Committee with a detailed written explanation of the termination or interruption of the study. Study records must be maintained.

10.13 Study Summary Report

After the end of the study, the investigator and the sponsor objectively summarize the study results, statistically analyze the study data with appropriate statistical methods, objectively evaluate the safety of the drug based on the results, and make a written summary report of this clinical study after review and consent of the sponsor.

10.14 Information Disclosure and Data Publishing Policies

The investigator should keep the information and data related to this study confidential and may not quote or publish the relevant study results or data without the consent of the sponsor.

The sponsor has the right to publish or publish information or data related to this study or to submit it to regulatory authorities. The sponsor should obtain the consent of the investigator if the name of the investigator needs to appear in the publication, publication or advertisement.

10.15 Conflict of Interest Statements

The investigator will provide the sponsor with adequate and accurate financial information as required by the relevant regulatory authority to enable the sponsor to submit complete and accurate financial statements or disclosure forms to the relevant regulatory authorities.

11 References

[1]Swerdlow, SH., Campo, E., Harris, NL.,et al. WHO Classification of Tumours of Haematopoietic and Lymphoid Tissues. Fourth. Vol. 2. WHO Press; 2008.

[2]Hervier B, et al. Association of both Langerhans cell histiocytosis and Erdheim-Chester disease linked to the BRAFV600E mutation. Blood. 2014; 124(7):1119–26.

[3]Haroche J, Cohen-Aubart F, Amoura Z. Erdheim-Chester disease[J]. Blood,2020,135(16):1311-1318.

[4]Cao X X, Li J, Zhao A L, et al. Methotrexate and cytarabine for adult patients with newly diagnosed Langerhans cell histiocytosis: A single arm, single center, prospective phase 2 study[J]. Am J Hematol,2020,95(9):E235-E238.

[5]Goyal G, Abeykoon J P, Hu M, et al. Single-agent cladribine as an effective front-line therapy for adults with Langerhans cell histiocytosis[J]. Am J Hematol,2021,96(5):E146-E150.

[6]Goyal G, Shah MV, Hook CC, et al. Adult disseminated Langerhans cell histiocytosis: incidence, racial disparities and long-term outcomes. Br J Haematol. 2018;182(4): 579-581.

[7]Carlos Rodriguez-Galindo, Carl E Allen. Langerhans cell histiocytosis. Blood. 2020 Apr 16;135(16):1319-1331.

[8]Allen CE, Merad M, McClain KL. Langerhans cell histiocytosis. N Engl J Med. 2018;379(9): 856-868.

[9]Rigaud C, Barkaoui MA, Thomas C, et al. Langerhans cell histiocytosis: therapeutic strategy and outcome in a 30-year nationwide cohort of 1478 patients under 18 years of age. Br J Haematol. 2016;174(6):887-898.

[10]Morimoto A, Shioda Y, Imamura T, et al; Japan LCH Study Group. Intensification of induction therapy and prolongation of maintenance therapy did not improve the outcome of pediatric Langerhans cell histiocytosis with single-system multifocal bone lesions: results of the Japan Langerhans Cell Histiocytosis Study Group-02 Protocol Study. Int J Hematol. 2018;108(2):192-198.

[11]Gao Y-J, Su M, Tang J-Y, Pan C, Chen J. Treatment outcome of children with multisystem Langerhans cell histiocytosis: the experience of a single children's hospital in Shanghai, China. J Pediatr Hematol Oncol.

2018;40(1):e9-e12.

[12]Morimoto A, Shioda Y, Imamura T, et al. Intensified and prolonged therapy comprising cytarabine, vincristine and prednisolone improves outcome in patients with multisystem Langerhans cell histiocytosis: results of the Japan Langerhans Cell Histiocytosis Study Group-02 Protocol Study. Int J Hematol. 2016;104(1):99-109.

[13]Saven A, Burian C. Cladribine activity in adult langerhans-cell histiocytosis[J]. Blood,1999,93(12):4125-4130.

[14]Miao H L, Zhao A L, Duan M H, et al. Clinical presentation and prognostic analysis of adult patients with Langerhans cell histiocytosis with pulmonary involvement[J]. BMC Cancer,2020,20(1):911.

[15]Cantu M A, Lupo P J, Bilgi M, et al. Optimal therapy for adults with Langerhans cell histiocytosis bone lesions[J]. PLoS One,2012,7(8):e43257.

[16]Badalian-Very G, Vergilio JA, Degar BA, et al. Recurrent BRAF mutations in Langerhans cell histiocytosis. Blood 2010;116:1919–23.

[17]Goyal G, Heaney M L, Collin M, et al. Erdheim-Chester disease: consensus recommendations for evaluation, diagnosis, and treatment in the molecular era[J]. Blood,2020,135(22):1929-1945.

[18] Chen J, Zhao A, Duan M, et al. Diverse kinase alterations and myeloid-associated mutations in adult histiocytosis[J]. Leukemia, 2021: 1-4.

[19]Goyal G, Heaney M L, Collin M, et al. Erdheim-Chester disease: consensus recommendations for evaluation, diagnosis, and treatment in the molecular era[J]. Blood,2020,135(22):1929-1945.

[20]Hyman DM, Puzanov I, Subbiah V, et al. . Vemurafenib in multiple nonmelanoma cancers with BRAF V600 mutations. N Engl J Med. 2015;373(8):726-736.

[21]Haroche J, Cohen-Aubart F, Emile JF, et al. . Reproducible and sustained efficacy of targeted therapy with vemurafenib in patients with BRAF(V600E)-mutated Erdheim-Chester disease. J Clin Oncol. 2015;33(5):411-418.

[22]Diamond EL, Subbiah V, Lockhart AC, et al. . Vemurafenib for BRAF V600-mutant Erdheim-Chester disease and Langerhans cell histiocytosis: analysis of data from the histology-independent, phase 2, open-label VE-BASKET study. JAMA Oncol. 2018;4(3):384-388.

[23]Cohen Aubart F, Emile JF, Carrat F, et al. . Targeted therapies in 54 patients with Erdheim-Chester disease, including follow-up after interruption (the LOVE study). Blood. 2017;130(11):1377-1380.

[24]Donadieu J, Larabi IA, Tardieu M, et al. Vemurafenib for refractory multisystem Langerhans Cell Histiocytosis in children: An international observational study. J Clin Oncol. 2019;37(31):2857-2865.

[25]Eli L. Diamond, Benjamin H. Durham, Gary A. Ulaner, et al. Efficacy of MEK inhibition in patients with histiocytic neoplasms. Nature. 2019 Mar;567(7749):521-524.

[26]Flaherty KT, Robert C, Hersey P, et al. Improved survival with MEK inhibition in BRAF-mutated melanoma. N Engl J Med 367:107–14, 2012.

[27]Schadendorf D, Amonkar MM, Milhem M, et al.: Functional and symptom impact of trametinib versus chemotherapy in BRAF V600E advanced or metastatic melanoma: quality-of-life analyses of the METRIC study. Ann Oncol 25:700–6, 2014.

[28]KOSELUGO (Selumetinib) label.

[29]Falchook GS, Lewis KD, Infante JR et al. Activity of the oral MEK inhibitor trametinib in patients with advanced melanoma: A phase 1 dose-escalation trial. Lancet Oncol 2012;13:782–789.

[30]Infante JR, Fecher LA, Falchook GS, et al.: Safety, pharmacokinetic, pharmacodynamic, and efficacy data for the oral MEK inhibitor trametinib: a phase 1 dose-escalation trial. Lancet Oncol 13:773–81, 2012.

[31]Kim KB, Kefford R, Pavlick AC, et al.: Phase II study of the MEK1/MEK2 inhibitor Trametinib in patients with metastatic BRAF-mutant cutaneous melanoma previously treated with or without a BRAF inhibitor. J Clin Oncol 31:482–9, 2013.

[32]Long GV, Stroyakovskiy D, Gogas H, et al. Combined BRAF and MEK inhibition versus BRAF inhibition alone in melanoma. N Engl J Med 371:1877–88, 2014.

[33]Banks M, Crowell K, Proctor A et al. Cardiovascular effects of the MEK inhibitor, trametinib: A case report, literature review, and consideration of mechanism. Cardiovasc Toxicol 2017;17:487–493.

[34]Robert C, Grob JJ, Stroyakovskiy D et al. Five-year outcomes with dabrafenib plus trametinib in metastatic melanoma. N Engl J Med 2019;281:626–636.

[35]G. V. Long, K. T. Flaherty, D. Stroyakovskiy, et al. Dabrafenib plus trametinib versus dabrafenib monotherapy in patients with metastatic BRAF V600E/K-mutant melanoma: long-term survival and safety analysis of a phase 3 study. Ann Oncol. 2017 Jul; 28(7): 1631–1639.

[36]Jean Jacques Grob, Mayur M Amonkar, Boguslawa Karaszewska, et al. Comparison of dabrafenib and trametinib combination therapy with vemurafenib monotherapy on health-related quality of life in patients with unresectable or metastatic cutaneous BRAF Val600-mutation-positive melanoma (COMBI-v): results of a phase 3, open-label, randomised trial. Lancet Oncol 2015 Oct;16(13):1389-98.

[37]Georgina V Long, Axel Hauschild, Mario Santinami, et al. Adjuvant Dabrafenib plus Trametinib in Stage III BRAF-Mutated Melanoma. N Engl J Med. 2017 Nov 9;377(19):1813-1823.

Attachment 1 Pharmacokinetic Sample Collection Schedule

Cycle	Time from dosing (hours)	Blood Collection Window (minutes)	PK Blood Collection Volume (ml)
C1D1	Predose 1	-	2
C2D1	Predose 1	-	2
	0.5~1	-	2
	1.5~3	-	2
	4~6	-	2
C4D1	Predose 1,2	-	2
C7D1	Predose 1,2	-	2
C10D1	Predose 1,2	-	2

PK:

1. The collection of pre-dose PK blood samples must be completed within 60 minutes prior to dosing.
2. Pre-dose on C2D1/C4D1/C7D1/C10D1 is also 24 hours after the last dose in the previous cycle, and pre-dose PK blood samples were also required if the patient did not receive dosing in this cycle due to AEs or other reasons. In addition, PK blood sampling may be performed within the visit window.

Attachment 2 Study Flow Chart

	Screening D-28~D-1	Continuous dosing period 1	End of treatment (± 7 days)	Safety Follow-up (Within 30 days [± 7 days] after last dose) 21	Survival follow-up (every 6 months [± 14 days] after safety follow-up) 22
		CXD1 (± 3 days)			
Informed Consent	X				
Demographics 2	X				
Study Enrollment/Exclusion Criteria Assessment	X				
Medical History	X				
Tumor history	X				
Vital Signs 3	X	X	X	X	
Physical Examination 4	X	X	X	X	
Performance Status Score 5	X	X	X		
Hematology and Chemistry 6	X	X	X	X	
Urinalysis 7	X	X	X	X	
Serum virology (HBV, HCV and HIV) 8	X				
Coagulation 9	X	X (as clinically indicated)			
Blood HCG test (if applicable)	X		X		
12-lead ECG 10	X	X	X	X	
Echocardiography or MUGA 11	X	X (as clinically indicated)			
Ophthalmic Examination 12	X	X (to be performed in parallel with RECIST 1.1 tumor assessment)	X	X	
PRC Assessment 13	X	X (Cycle 4, Cycle 7, Cycle 13, Subsequent Disease Response) 12	X		

	Screening D-28~D-1	Continuous dosing period 1<br>CXD1 (± 3 days)	End of treatment (± 7 days)	Safety Follow-up (Within 30 days [± 7 days] after last dose) 21	Survival follow-up (every 6 months [± 14 days] after safety follow-up) 22
RECIST Assessment 14	X	X (Cycles 4, 7, 10, 13, subsequently based on disease response) 13	X		
Quality of Life Assessment 15	X	X (performed in parallel with RECIST 1.1 tumor assessment) 14	X		
Concomitant Medications/Concomitant Therapy16	X	X			
Detection of MAPK pathway gene mutations 17	X	X (performed in parallel with RECIST 1.1 tumor assessment) 16	X		
AE and SAE Evaluation 18	X	X			
PK blood collection 19		X			
Dispensing and Return of Study Drug and Dosing Diary Cards 20		X	X		
Survival and anti-tumor therapy					X

1. Continuous dosing period: the continuous dosing period is 28 days as a cycle.
2. Demographics: including date of birth, gender, race, ethnicity, alcohol consumption history, and smoking history.
3. Vital signs included: blood pressure, heart rate, respiration, and body temperature. Blood pressure should be measured after the patient has been sedentary for 5 minutes.
4. Physical examination included: height (at screening only), weight, head, eyes, ears, nose, throat, neck, heart, chest, abdomen, extremities, skin, lymph nodes, nervous system, and general condition of the patient.
5. Performance status score: ECOG score, see Annex 4.
6. Hematology: red blood cell count, hemoglobin, hematocrit, white blood cell count and differential (neutrophils, lymphocytes, eosinophils, monocytes, basophils, and other cells) and platelet count. Blood chemistry: total protein, albumin, blood glucose, total cholesterol, triglycerides, urea/urea nitrogen, creatinine, alkaline phosphatase, gamma-glutamyl transpeptidase, lactate dehydrogenase, total bilirubin, direct bilirubin, AST, ALT, calcium, phosphorus, magnesium, potassium, sodium, chloride, serum amylase, erythrocyte sedimentation rate, C-reactive protein, CK, CK-MB, uric acid. If the above test results are available within 7 days prior to Day 1, it is not necessary to repeat the test before the first dose on Day 1.
7. Urinalysis: specific gravity, pH, glucose, protein, ketones, red blood cells and white blood cells. If the above test results are available within 7 days prior to Day 1, it is not necessary to repeat the test before the first dose on Day 1.

8. Serum virology: testing for HBV, HCV, and HIV is done. Five items of hepatitis B and five items of hepatitis B should be tested for HBV: HBsAg, HBsAb, HBeAg, HBeAb and HBcAb. HBV DNA copy number testing is required for patients who are positive for HBsAg, HBeAg, HBeAb, and HBcAb, and hepatitis C virus RNA (HCV RNA) is required for HCV-IgG positive patients.

9. Coagulation tests: including PT, APTT, INR. Items considered mandatory at screening visits; if screening test results are not clinically significant, repeat testing on the day of the first dose is not required and may be performed as clinically indicated thereafter.

10. 12-lead ECG: after resting for at least 5 minutes in semi-recumbent or supine position, patients should undergo 3 examinations by qualified center staff.

11. Echocardiography or MUGA: once at screening and subsequently as clinically indicated.

12. Ophthalmologic examination: including visual acuity, visual field, slit lamp fundus examination, intraocular pressure, fundus photography/scanning laser fundus examination, and ophthalmologic B-ultrasound.

13. PRC standard assessment: 18FDG-PET/CT examination range from skull top to plantar. Tumor imaging will be assessed by the investigator and IRC according to PRC, respectively. Tumor assessments may be increased by the investigator as clinically indicated. The investigator should make subsequent treatment decisions based on his/her response evaluation results. Tumor assessments may be used as baseline tumor assessments if they have been performed within 28 days prior to the first dose and the same method and machine are used in the same hospital. Tumor assessments will be performed once at screening, at the end of treatment cycles 3, 6, 12 (± 7 days), and at the end of treatment. Patients evaluated as CMR or PMR had confirmed response by repeated assessments at least 4 weeks later. Tumor assessments per PRC criteria will be performed every 3-6 treatment cycles (± 14 days) if, in the opinion of the investigator, the imaging results are not consistently stable after 12 treatment cycles. If a patient discontinues treatment, tumor assessments should continue as scheduled until disease progression, death, withdrawal of informed consent, loss to follow-up, start of new anti-tumor therapy, or end of the study. At the End of Treatment Visit, if tumor imaging has been performed in the first 8 weeks, tumor imaging may not be repeated at the End of Treatment Visit.

14. RECIST Version 1.1: including MRI/CT of the head, neck, chest, abdomen, and pelvis. Target lesions indicated by PET-CT or new lesions during treatment were selected or added, but the same imaging assessment method (MRI/CT) should be used for each patient throughout the treatment. Tumor imaging will be assessed by the investigator and IRC according to RECIST v1.1, respectively. Tumor assessments may be performed more frequently by the investigator as clinically indicated. The investigator should make subsequent treatment decisions based on their own response evaluation results. Tumor assessments may be used as baseline tumor assessments if they have been performed within 28 days prior to the first dose and the same method and machine are used in the same hospital. Tumor assessments will be performed at screening, at the end of treatment cycles 3, 6, 9, 12 (± 7 days), and at the end of treatment. If the imaging results are considered to be consistently stable by the Principal Investigator after 12 treatment cycles, tumor assessments will be performed every 4 treatment cycles (± 14 days) according to RECIST 1.1. If the imaging results do not reach sustained stability after 12 treatment cycles, tumor assessments will be performed every 3 treatment cycles (± 7 days) according to RECIST 1.1. If a patient discontinues treatment for reasons other than disease progression, tumor assessments should continue as scheduled until disease progression, death, withdrawal of informed consent, loss to follow-up, start of new anti-tumor therapy, or end of the study. At the End of Treatment Visit, if tumor imaging has been performed in the first 4 weeks, tumor imaging may not be repeated at the End of Treatment Visit.

15. Quality of life assessment: all patients were assessed for quality of life using the EORTC QLQ-C30 scale, see Attachment 3.

16. Concomitant Medications/Concomitant Therapy: record from 28 days prior to the first dose until 30 days after the last dose or until the start of other anti-tumor therapy, whichever occurs first.

17. It is recommended that patients provide a sufficient number of histopathological slides or tumor tissue samples for central laboratory testing of the following biomarkers, including but not limited to ERBB3, RAF-1, BRAF, ARAF, HRAS, KRAS, NRAS, MEK (MAP2K1 and MAP2K2) and other MEK upstream gene mutations prior to enrollment. Peripheral blood will be collected at screening and subsequently for the detection of MAPK and other pathway gene mutations in cfDNA.

18. Adverse Events: collect from signing the informed consent form until 30 days after the last dose or until the start of other anti-tumor therapy, whichever occurs first. Study drug-related AEs/SAEs should also be collected and reported after the endpoints of AE/SAE collection.

19. PK blood sampling: refer to Appendix 1 Pharmacokinetic Sample Collection Time List.

20. Dispensing and retrieval of study drug and dosing diary cards: the study drug should be dispensed to the patient on the first day of each cycle. Dispensing of study drug in the new cycle should be performed after completion of assessments including laboratory tests, etc., to confirm that the patient is safe to continue taking the investigational product. Patients should retain all unused medications and bottles and return them at the next visit.

21. Safety Follow-up: the Safety Follow-up Visit is performed 30 days (± 7 days) after the last dose, and if the patient starts subsequent anti-tumor therapy within 30 days after the last dose, the Safety Follow-up Visit should be completed before the patient starts a new anti-tumor treatment. If a patient is unable to return to the study site for a safety visit due to physical condition, a remote visit may be performed and the local test results may be acceptable.

22. Survival follow-up: follow-up will be performed every 6 months (± 14 days) by telephone, text message, or other remote means until the end of the study, taking the end of treatment visit or the safety visit as the starting point, whichever occurs later.

Attachment 3 Quality of Life Inventory for Oncology Patients EORTC QLQ-C30 (V3)

We would like to know something about you and your health. Please answer all the questions below in person. The answers here are not "right" or "wrong", but only ask to circle the number that best reflects your situation. The information you provide will be kept strictly confidential.

Please fill in the first letter of your name:________

Date of birth: dD.M.

Today Date: dD.M.

	No	A	Equivalent	Very
1. Do you have any difficulty in engaging in some laborious activities,	1	2	3	4
2. Is it difficult for you to walk long distances?	1	2	3	4
3. Is it difficult for you to walk short distances	1	2	3	4
4. Do you need to stay in bed or chair during the day?	1	2	3	4
5. Do you need help eating, dressing, bathing or going	1	2	3	4

**During the past week:**	**No**	**A**	**Equivalent**	**Very**
6. Are you restricted in your work and other daily	1	2	3	4
7. Are you restricted in your hobbies or leisure	1	2	3	4
8. Have you had any shortness of breath?	1	2	3	4
9. Have you had pain?	1	2	3	4
10. Do you need a rest?	1	2	3	4
11. Have you had trouble sleeping?	1	2	3	4
12. Do you feel weak?	1	2	3	4
13. Do you have poor appetite (no appetite)?	1	2	3	4
14. Do you feel nauseous?	1	2	3	4
15. Have you vomited?	1	2	3	4
16. Do you have constipation?	1	2	3	4

Please proceed to next page

During the past week:	No	A	Equivalent	Very
17. Have you had diarrhea?	1	2	3	4
18. Do you feel tired?	1	2	3	4
19. Does pain affect your daily activities?	1	2	3	4
20. Have you had difficulty concentrating on things,	1	2	3	4
21. Do you feel nervous?	1	2	3	4
22. Do you feel anxious?	1	2	3	4
23. Do you feel irritable?	1	2	3	4
24. Do you feel depressed (depressed mood)?	1	2	3	4
25. Do you have trouble remembering?	1	2	3	4
26. Does your physical condition or treatment affect	1	2	3	4
27. Does your physical condition or treatment affect	1	2	3	4
28. Did your physical condition or treatment make you	1	2	3	4

For the following questions, please select a number between 1 and 7 that best suits you and circle it.

29. How do you rate your overall health over the past week?

1 2 3 4 5 6 7

Very bad very good

30. How do you rate your overall quality of life over the past week?

1 2 3 4 5 6 7

Very bad very good

Annex 4 ECOG Performance Status

Grade	ECOG
0	Completely normal, able to carry out all normal activities without restriction
1	Unable to perform strenuous physical activity, but ambulatory and able to engage in light [illegible]
2	Ambulatory, capable of self-care, but unable to carry out any work, confined to bed for no [illegible]
3	Can barely take care of oneself and need to lie in bed or sit in a chair for more than 50% of [illegible]
4	Completely incapacitated, severely incapable of self-care, confined to bed or wheelchair
5	Death

Annex 5 New York Heart Association functional class (NYHA)

Grade	Physical activity	Resting state	Symptoms (fatigue, palpitations, wheezing, or angina)
I	Not limited	Asymptomatic	Not caused by general physical activity
II	Mild limitation	Asymptomatic	Daily physical activity can cause
III	Marked limitation	Asymptomatic	Caused by less than usual physical activity
IV	Loss	Symptomatic	Worsening of any physical activity

Attachment 6 COCKCROFT-GAULT Formula

$$Ccr = \frac{(140-\text{年龄})\times\text{体重}(kg)}{72\times Scr(mg/dl)}(\text{女性}\times 0.85)\ \textbf{Or}\ Ccr = \frac{(140-\text{年龄})\times\text{体重}(kg)}{0.818\times Scr(umol/L)}(\text{女性}\times 0.85)$$

Note: units of creatinine, 1 mg/dL = 88.4 umol/L, Female result × 0.85.

Appendix 7 Response Evaluation Criteria in Solid Tumors (RECIST version 1.1)

Response Evaluation Criteria in Solid Tumors Version 1.1

(Response Evaluation Criteria in Solid Tumors RECIST Version 1.1)

Since no official Chinese version of RICIST 1.1 has been published at present, this version is an in-house translation. For more details, please refer to the English version http://ctep.cancer.gov/protocolDevelopment/docs/recist _ guideline.pdf.

Synopsis

Background Introduction

Evaluating changes in tumor burden is an important feature of the clinical evaluation of cancer therapy. Tumor shrinkage (objective response) and disease progression are both meaningful endpoints in clinical trials. Since RECIST was published in 2000, many researchers, association groups, businesses, and government authorities have adopted this criterion to evaluate treatment effect. However, some of the problems that followed led to the publication of this revised edition (version 1.1). The modifications (see topics in each chapter) were derived from assessments of large databases (over 6500 patients), simulation studies, and literature reviews.

Important revisions to RECIST v1.1

The main revisions are:

Determination of number of lesions: to facilitate analysis, the evaluation of many trial data was combined into a database from which the total number of lesions required to determine tumor burden at the endpoint was reduced from a maximum of 10 to a maximum of 5 (from a maximum of 5 to 2 per organ).

The determination of pathological lymph nodes is now combined to determine that nodes with a short axis of 15 mm are measurable as target lesions for evaluation. When determining tumor response, the short axis value (for nodal lesions) must be included in the sum of lesions (radii). Nodules can be considered normal when they shrink to a short axis of < 10 mm.

Confirmation of efficacy is required in clinical trials where response rate is the primary endpoint, but is no longer required in randomized controlled clinical trials, as the control arm has become an effective means of interpreting trial data. Progressive disease is addressed in the following ways: in addition to the original definition of a 20% increase in the sum of target lesions (radii), if the total number is small, an absolute increase of 5 mm (in the short axis of the lesion) must now be available to prevent overestimation of the extent of worsening. In addition, guidance on "unequivocal worsening" of non-measurable or non-target lesions is provided – i.e., where the original RECIST guidelines are easily confused. A final section is dedicated to the detection of new lesions, including interpretation of FDG-PET scans. Radiology Guidelines: the revised RECIST contains a new imaging appendix with updated recommendations for best anatomical assessment of lesions.

Next steps:

A key issue considered by the Working Group when revising RECIST v1.1 is whether it is appropriate to assess tumor burden from a one-dimensional anatomical assessment to a three-dimensional anatomical assessment or a functional assessment using PET and MRI. The current conclusion is that there are insufficient criteria or evidence to abandon anatomical assessment of tumor burden. The only explanation for this is the use of FDG-PET imaging as an adjunct to the judgment of worsening disease. As discussed in detail in the topics of the last chapter, the use of these latest, promising technologies requires appropriate clinical validation studies.

Keywords: response assessment criteria; solid tumors; guidelines

1. Background

1.1. history of RECIST criteria

Evaluating changes in tumor burden is an important feature of the clinical evaluation of cancer therapy. Tumor shrinkage (objective response) and time to disease progression are important adjudication endpoints in cancer clinical trials. To screen for new antineoplastic agents, years of evidence supports tumor shrinkage as an endpoint in phase II trials. These studies suggest that for multiple solid tumors, drugs that cause tumor shrinkage in some patients may later (albeit imperfect) prove to improve overall survival or have other opportunities for event evaluation in randomized phase III trials. Objective response is currently more reliable than any other biomarker in measures evaluating treatment effect in phase II screening trials. Moreover, in phase II and III clinical trials of drug development, time to progression (or PFS) is increasingly being used as an endpoint for efficacy determination in clinical trials in severely ill conditions, which are also based on anatomical measurements of tumor size.

However, objective response and time to progression, the two tumor-determining endpoints, are of value only if they are based on widely accepted and easy-to-use standard criteria based on the anatomy of tumor burden. Tumor response criteria were first published by the World Health Organization (WHO) in 1981 and are mainly used in trials where tumor response is the primary endpoint. The WHO criteria introduce the concept of an overall assessment of tumor burden by measuring the two-dimensional size of lesions and performing a total, and judgement of response to treatment by evaluating changes in baseline during treatment. However, for more than a decade after publication of the standard, collaborative groups and pharmaceutical companies using the standard have generally modified it to accommodate new technologies or to present unclear areas in the original literature, which leads to confusion in the interpretation of trial results. In fact, the application of various response criteria results in a wide variation in the treatment effect of the same treatment. In response to these issues of response, an international working group was established in the mid-19th century to standardize and simplify the response criteria.

The new criteria, also known as RECIST (Response Evaluation Criteria in Solid Tumors), were published in 2000. The initial RECIST key features include the determination of the smallest measurable lesion size, a description of the number of follow-up lesions (up to 10; maximum 5 per organ), the use of one dimension rather than two dimensions, and the overall

evaluation of tumor burden. These criteria were later widely adopted by academic groups, collaborative groups, and the pharmaceutical industry, and the initial endpoint of the criteria was objective response or disease progression. In addition, RECIST was accepted by the Agency as an appropriate criterion for these evaluations.

2. The purpose of this guideline is:

This guideline describes a standard method for measuring solid tumors and addresses objective criteria for determining changes in tumor size used in clinical trials of adult and pediatric cancer. These criteria are expected to be used in all trials with objective response as the primary endpoint, as well as trials using steady-state disease evaluation, tumor progression, or analysis of time to progression as all measures of treatment effect are based on the assessment of anatomical tumor burden and its changes in the study. There are no assumptions in this article about the proportion of patients who meet the inclusion criteria, all of whom use trial endpoints that predict the effectiveness of a drug or treatment regimen: those definitions that depend on the type of cancer in the ongoing trial and the particular drug under study. The protocol must include an appropriate statistical section defining the size of the trial sample and the efficacy parameters justified by the inclusion criteria. In addition to providing definitions and criteria for determining tumor response, this guideline also makes recommendations for standard reporting of clinical trial results using tumor response as an endpoint.

Although these guidelines can be used for the study of malignant brain tumors, separate criteria have been published in this area for the assessment of response. Since international guidelines for lymphoma response assessment have also been published separately, this guideline is not used in studies of malignant lymphomas.

Finally, many oncologists rely on multiple imaging studies in their daily clinical practice to track patients with malignant disease and decide on further treatment options based on objective and symptomatic double standards. These RECIST guidelines will play an important role in decision making only if judged reasonable by the treating oncologist.

3. Baseline Tumor Measurements

3.1 Definition

Tumor lesions/lymph nodes will be classified as measurable versus non-measurable at baseline as follows

3.1.1 Measurable

Neoplastic lesions: at least one dimension that is not less than the lower limit of (instrumental detection) must be accurately measured (the longest diameter on the measuring instrument will be recorded):

- **10 mm CT scan (CT scan slice thickness not more than 5 mm).**
- **Clinical examination 10 mm is measured with a caliper (lesions that cannot be accurately measured with calipers should be recorded as non-measurable).**
- **20 mm chest X-ray.**

Malignant lymph nodes: lymph nodes must have a short axis of 15 mm to be considered pathologically enlarged and measurable when assessed with a CT scan (CT scan slice thickness

recommended to be no greater than 5 mm). Only the short axis length was measured and tracked preoperatively and at follow-up. Lymph node measurements can also be obtained from the annotations under "Preoperative Documentation of Target and Non-Target Lesions".

3.1.2 Non-measurable (tumor)

All other lesions, including small lesions (longest diameter less than 10 mm or pathological lymph nodes with a short axis of 10 mm to less than 15 mm) as well as truly non-measurable lesions. Lesions considered truly unmeasurable include: meningeal disease identified by pharmacological examination, ascites, pleural or pericardial effusion, inflammatory breast disease, skin or lung with lymphatic involvement, abdominal mass/abdominal organomegaly that cannot be measured by reproducible imaging techniques.

3.1.3 Special Considerations for Measurable Lesions

Special attention should be paid to bone lesions, cystic lesions, and lesions previously treated locally:

Bone lesions:

• Bone scans, PET scans, or plain films are considered inadequate imaging techniques for measuring bone lesions. However, pharmacologically these techniques can be used to confirm the presence or disappearance of bone lesions.

• Osteolytic lesions with identifiable soft tissue or mixed acute lytic lesions can be considered measurable lesions when they can be assessed by cross-sectional imaging techniques such as CT or MRI, if the soft tissue portion meets the definition of measurability described above.

• Osteoblastic lesions are non-measurable.

Cystic lesions:

• X-ray-defined simple cysts that meet the inclusion criteria should not be considered malignant lesions (neither measurable nor non-measurable) as they are, by definition, simple cysts.

• "Cystic lesions" presenting as cystic metastases can be considered measurable lesions if the definition of measurable disease as described above is met. However, if a non-cystic lesion is present in the same patient, the target lesion is preferentially enrolled.

Lesions previously treated locally:

• Tumor lesions located in previously irradiated areas or sites subjected to other local therapy are generally not considered measurable unless it has been demonstrated that the lesion continues. The protocol should specify the conditions under which such lesions will be considered measurable.

3.2 Specification for Measurement Methods

3.2.1 Measurement of Lesions

Clinical assessments were measured with a caliper (caliper) and all measurements were recorded in metric units. All baseline assessments must be performed as close as possible to the start of treatment and not earlier than four weeks.

3.2.2 Measuring method

The same adjudication methods and techniques should be used to describe each reported lesion at baseline and follow-up. Unless follow-up reveals lesions that are not amenable to imaging, imaging rather than clinical examination should usually be used.

Clinical lesions: only superficial lesions (eg, subcutaneous nodules) measuring more than 10 mm in diameter using calipers are considered measurable. For cases of skin lesions, it is recommended to record with color photographs attached to measure the proportion of lesion size. As previously described, imaging should be used when lesions are available for both clinical and imaging because radiographic evaluation is more objective and can be used in the final review of clinical studies.

Chest X-ray: chest CT is preferred over chest X-ray, particularly when disease progression is used as an important endpoint because CT scans are more sensitive than X-rays in identifying new lesions. However, lesions on x-rays are considered measurable if they are clearly circumscribed and surrounded by inflated lungs.

CT, MRI: CT is currently the most effective and reproducible method for assessing lesion response. Guidelines define measurable lesions using CT scans based on slice thickness of no more than 5 mm. As shown in Appendix 2, when CT slice thickness exceeds 5 mm, measurable lesions should be at least twice the slice thickness. MRI may also be used in some circumstances (eg, whole-body scan). Further comments on the use of CT and MRI in the assessment of response in solid tumors are provided in Appendix II.

Ultrasonography: ultrasound is not indicated to assess lesion size and should not be used for measurement methods. Ultrasonography is not completely reproducible between two adjacent observations and results depend on the examiner, and the same technique and measurement cannot be guaranteed from one test to the next. If new lesions are identified by ultrasound during the course of the study, confirmation with CT or MRI is recommended. MRI may be used instead to detect lesions to be examined if radiation exposure to CT is a concern.

Endoscopy, laparoscopy: these techniques are not recommended for objective tumor evaluation. However, they are beneficial in demonstrating complete pathological response by biopsy or in determining complete response or recurrence after surgical resection.

Tumor markers: tumor markers cannot be used alone to assess objective tumor response. However, when tumor markers start to be above the upper limit of normal, markers must be standardized if used to determine complete response in patients. Because tumor markers are disease specific, the measurement technical instructions should indicate the documentation of baseline testing for a particular disease. Special guidelines on CA-125 change (in ovarian cancer recurrence) and PSA change (in prostate cancer recurrence) have been published. In addition, the International Group on Gynecologic Oncology (Intergroup) has developed CA 125 progression criteria that apply the overall first-line approach to objective evaluation of tumors in ovarian cancer trials.

Cytology, histology: these techniques can often be used to differentiate partial and complete responses in individual cases (e.g., residual benign tumor lesions of germ cell tumors) if required by clinical study protocols. When exudates are known to be potentially serious adverse outcomes of treatment (e.g., certain paclitaxel chemotherapeutic agents or angiogenesis inhibitors), attention needs to be paid to any cytologically confirmed new exudate that appears or worsens during treatment in order to distinguish between treatment response (e.g., stable

disease) and disease progression, even if measurable tumors meet criteria for efficacy or stability.

4. Tumor Response Assessment

4.1 Assessment of All Tumors and Measurable Lesions

To evaluate objective response or possible future progression, it is necessary to perform a baseline assessment of the total tumor burden of all tumor lesions as a reference for subsequent measurements. In clinical protocols with objective response as the primary treatment endpoint, only patients with measurable disease at baseline can be enrolled. Measurable disease is defined as the presence of at least one measurable lesion. For trials where disease progression (time to progression or extent of progression on a fixed date) is the primary endpoint, protocol inclusion criteria must specify whether they are limited to patients with measurable disease or no measurable disease can also be included.

4.2 Baseline documentation of target and non-target lesions

When more than one measurable lesion is present at baseline assessment, all lesions up to a total of no more than 5 lesions (no more than 2 per organ) should be recorded and measured as target lesions representing all involved organs (i.e., a maximum of two or four target lesions will be selected as baseline measurable lesions for patients with only one or two cumulative organs).

Target lesions must be selected based on size (longest diameter), representative of all involved organs, and measurements must be reproducible. Occasionally, when the largest lesion cannot be measured reproducibly, one of the largest lesions that can be measured reproducibly can be re-selected.

Lymph nodes require special attention because they are normal tissue and are radiologically detectable even in the absence of tumor metastasis. Pathological lymph nodes, defined as measurable nodes or even target lesions, must meet the following criteria: short diameter ≥ 15 mm as measured by CT. Only short diameters are required at baseline. Radiologists usually rely on the short diameter of a node to determine whether the node has metastasized. Nodal size is generally represented by two-dimensional data from imaging (CT is the axial plane, and MRI selects a plane from the axial, sagittal, or coronal planes). The smallest value is the short diameter. For example, a 20 mm × 30 mm abdominal node with a short diameter of 20 mm can be considered a malignant, measurable node. In this example, 20 mm is the measurement of the node. Nodes ≥ 10 mm in diameter but < 15 mm should not be considered target lesions. Nodules < 10 mm do not fall into the category of pathological nodules and need not be recorded and further observed.

The calculated sum of diameters for all target lesions (including the longest diameter for non-nodal lesions and the shortest diameter for nodal lesions) will be reported as the baseline sum diameters. If lymph node diameters are included, as mentioned above, only short diameters will be counted. The baseline sum of diameters will be used as the reference value for the baseline level of disease.

All remaining lesions, including pathological lymph nodes, may be considered non-target lesions and do not require measurement but should be recorded at baseline assessment. If recorded as ‘ present ’, ‘ missing ’ or rarely ‘ unequivocal progression ’. Extensive target lesions may be recorded with the target organ (e.g., extensive expansion of pelvic lymph nodes or large-scale liver metastases).

4.3 Response Assessment Criteria

Criteria used to determine ORR in tumor target lesions are defined in this section.

4.3.1 Response Evaluation of Target Lesions

Complete response (CR): disappearance of all target lesions and any pathological lymph node (whether target or not) must have a short axis value of < 10 mm.

Partial response (PR): at least a 30% decrease in the sum of diameters of all target lesions, taking as reference the total diameters at baseline status.

Progression of Disease (PD): at least a 20% increase in the sum of diameters of all target l esions, taking as reference the smallest lesion sum diameters (including the sum of diameters o f lesions at baseline if it is the nadir). In addition, in addition to a relative increase of 20% in th e sum of diameters, the absolute value of the sum must also increase by at least 5 mm (Note: t he appearance of one or more new lesions may also be considered progression).

Stable disease (SD): with reference to the smallest sum of lesion diameters during the study, lesion shrinkage is not consistent with either PR, increase in lesions, or PD.

4.3.2 Precautions for Response Evaluation of Target Lesions

When the target lesion is lymph node:

Their actual short axis measurement (in the same anatomical plane as the baseline measur ement) should usually be recorded, even if all lymph nodes on study regress below 10 mm. Th is means that when the target lesion is a lymph node, the sum of diameters of the target lesion will not be 0 even if the criteria for complete response are met, since lymph nodes with a short axis value < 10 mm are defined as normal lymph nodes. Case report forms or other data colle ction methods may be designed to record nodal target lesions individually, and each node must have a short axis value < 10 mm in order to determine complete response. For PR, SD, and P D, the sum of target lesions (diameters) will include the actual short axis of the nodes.

Target lesions too small to measure:

All lesions (nodal and non-nodal) recorded at baseline on study must have their actual measurements recorded at subsequent assessments, albeit very small (e.g., 2 mm).

However, sometimes when a lesion or lymph node is recorded at a cut-off point, the radiologist may not be happy to give an accurate measurement because the signal is too weak on CT scan and instead report it as "too small to measure".

When this occurs, it is important to include a measurement in the case report form. If, in the

opinion of the radiologist, the lesion may disappear, the measurement can be recorded as 0 mm. If the lesion is present and the signal is too weak, it can be recorded as the default value of 5 mm (this rule is not appropriate for lymph nodes because normal lymph nodes have a clear value and are frequently encapsulated by adipose tissue, such as lymph nodes in the retroperitoneal cavity; however, if lymph nodes are present but the signal is too weak to measure, it can also be recorded as the default value of 5 mm).

The default value of 5 mm is derived from the thickness of the CT scan slice (if this thickness is changed, the default value of 5 mm should not be changed). The measurement of such (too small to measure) lesions may lack reproducibility and giving a default value prevents false cure or false worsening when the measurement error is assessed. Again, if the radiologist can give an actual measurement, even if it is less than 5 mm, it should be recorded.
Lesions with treatment-emergent disintegration or fusion

When non-nodal lesions "fragment", the longest diameters of all fragments must be added together to calculate the target lesion sum diameters, as noted in Appendix II. Similarly, when lesions coalesce, the long diameter between them can be preserved, which helps to obtain the maximum diameter value of each lesion prior to merging. If lesions coalesce completely and no longer separate from each other, the vector of the longest diameter in this case is the largest longest diameter of the coalesced lesion.

4.3.3 Assessment of Non-Target Lesions

Response criteria for tumors with non-target lesions are defined in this section. Although some non-target lesions are actually measurable, they do not need to be measured and only need to be qualitatively assessed at protocol-specified timepoints.

Complete response (CR): disappearance of all non-target lesions and return of tumor markers to normal levels. All lymph nodes were of non-pathological size (< 10 mm short axis).

Non-complete response/non-progressive disease (non-CR or non-PD): presence of one or more non-target lesions and/or persistence of tumor marker levels beyond normal.

Progressive disease (PD): unequivocal progression of pre-existing non-target lesions. Note: the appearance of one or more new lesions is also considered progressive disease.

4.3.4 Special precautions regarding assessment of progression of non-target lesions

The additional explanation for the definition of progression of non-target lesions is as follows: when a patient has measurable non-target disease, the definition of unequivocal progression on the basis of non-target disease, even if the target lesion assessment is stable or partial response, must meet that the overall worsening of non-target lesions has reached the point where treatment must be discontinued. Whereas a general increase in the size of one or more non-target lesions is often not sufficient to meet the criteria for progression, it is virtually rare that overall tumor progression can be defined by changes in non-target lesions alone when target lesions are stable or partial responses.

When none of the patients had measurable non-target lesions: this occurred in some Phase 3 trials when the inclusion criteria did not specify that measurable disease had to be present. The global assessment is still based on the criteria above, but in this case there is no measurable data for the lesion. Worsening of non-target lesions is not easily assessable (by definition: all non-target lesions must be truly non-measurable), so when changes in non-target lesions result in an increase in overall disease burden equivalent to disease progression in target lesions, a clear definition of progression based on non-target lesions requires the establishment of an effective test method for assessment. As described, the increase in tumor burden corresponds to an additional 73% increase in volume (equivalent to a 20% increase in the diameter of measurable lesions). such as peritoneal exudate from "trace" to "large" ; lymphangiopathy ranged from "local" to "widespread" ; or described in the protocol as "sufficient to change treatment". Examples include pleural exudate from trace to large, lymphatic involvement spreading distally from the primary site, or may be described in protocols as "necessitating therapeutic changes". If unequivocal progression is found, the patient should be considered progression overall at that time point. It is preferable that objective criteria be available for the assessment of non-measurable lesions, noting that the added criteria must be reliable.

4.3.5 New Lesions

The appearance of new malignant lesions predicts disease progression; therefore, some evaluation of new lesions is important. There are no specific criteria for imaging lesions, however, the finding of a new lesion should be unequivocal. For example, progression cannot be attributed to differences in imaging techniques, changes in imaging morphology, or lesions other than tumors (e.g., some so-called new bone lesions are simply healing of the original lesion, or recurrence of the original lesion). This is important when a patient has a partial or complete response to a baseline lesion, e.g., necrosis of a liver lesion may be identified as a new cystic lesion on CT report rather than it is.

Lesions detected at follow-up that are not identified at baseline will be considered new lesions and suggestive of disease progression. For example, a patient who has visceral disease at baseline will be considered as evidence of disease progression when he has a CT or MRI skull examination, even if he does not have a skull examination at baseline.

If a new lesion is equivocal, e.g. due to its small morphology, further treatment and follow-up evaluation is required to confirm whether it is a new lesion. If repeat testing confirms that it is a new lesion, the time to progression should be calculated from the time of its initial finding.

FDG-PET assessment of lesions generally requires additional testing for supplemental confirmation, and a combination of FDG-PET and supplemental CT findings is reasonable to evaluate progression (particularly for new suspected disease). New lesions can be identified by FDG-PET and will be performed according to the following procedures:

Baseline FDG-PET was negative and subsequent follow-up FDG-PET was positive, indicating disease progression.

No baseline FDG-PET was performed and subsequent FDG-PET was positive:

If a new lesion identified by a positive FDG-PET at follow-up is consistent with a CT scan, disease progression will be demonstrated.

If a new lesion identified by a positive FDG-PET at follow-up is not confirmed by CT, CT is required to confirm it (if confirmed, the time to progression is calculated from the previous FDG-PET abnormality).

If a positive FDG-PET at follow-up is consistent with an existing lesion on CT that does not progress on imaging, the disease does not progress.

4.4 Best overall response evaluation

The best overall response evaluation is the best response recorded from the start of the trial to the end of the trial, taking into account any necessary conditions for confirmation. Sometimes the response occurs after the end of treatment, so the protocol should clarify whether the response evaluation after the end of treatment is considered within the best overall response evaluation. The protocol must clarify how any new treatment prior to progression affects the best response. The patient ’ s best response depends mainly on the results of target and non-target lesions and the appearance of new lesions. In addition, it relies on the nature of the trial, protocol requirements, and outcome measures. Specifically, in nonrandomized trials, response is the primary objective, and confirmation of response to PR or CR is necessary to confirm which is the best overall response.

4.4.1 Time point reaction

Efficacy responses were assumed to occur at specific time points for each regimen. Table 1 will provide a summary of the overall response at each time point for a patient population with measurable disease at baseline.

If a patient has no measurable disease (no target lesion), the assessment can be found in Table 2.

4.4.2 Missing and Non-evaluable Instructions for Assessments

If a lesion cannot be imaged or measured at a particular time point, the patient is not evaluable at that time point. If only a subset of lesions can be evaluated in an evaluation, this is generally considered not evaluable at that time point, unless there is evidence that the missing lesion does not affect the response evaluation at a given time point. This is likely to occur in the context of disease progression. For example, if a patient has 3 lesions at baseline that sum 50 mm, but then only 2 lesions are evaluable with a sum of 80 mm, the patient will be evaluated as progressive disease, regardless of the impact of missing lesions.

4.4.3 Best overall response: all time points

The best overall response can be determined once all data are available for the patient.

Best overall response assessment when confirmation of complete or partial response is not required in the study: the best response in the trial is the best response at all time points (e.g., a patient has SD at Cycle 1, PR at Cycle 2, PD at the last cycle, but has a best overall response of

PR.When the best overall response is assessed as SD, it must meet the minimum time from baseline as specified in the protocol. If criteria for the shortest time are not met, even a best overall response assessment of SD is not recognized, and the patient ' s best overall response will depend on subsequent evaluation. For example, a patient who has SD at Cycle 1 and PD at Cycle 2, but does not meet the minimum time requirement for SD, will have a best overall response of PD. The same patient lost to follow-up after assessment of SD in Cycle 1 will be considered non-evaluable.

Best overall response assessment when confirmation of complete or partial response is required in the study: complete or partial response can only be declared if each patient meets the trial-specified criteria for partial or complete response and is confirmed again at a subsequent time point (generally four weeks later) specifically mentioned in the protocol. In this case, the best overall response is described in Table 3.

4.4.4 Special hints for response assessment

When nodal lesions are included in the overall target lesion assessment and the nodes decrease in size to "normal" size (< 10 mm), they will still have a lesion size scan report. In order to avoid overestimation based on an increase in nodule size, measurements will be recorded even if the nodes are normal. As already mentioned, this means that patients with a response of complete response will not be recorded as 0 on the CRF form.

If confirmation of response is required during the trial, repeated "non-measurable" time points will complicate the best response assessment. The analysis plan for the trial must state that these missing data/assessments can be explained clearly when determining efficacy. For example, in most trials, a patient ' s response to PR-NE-PR can be considered confirmed.

Symptomatic progression should be reported when a patient experiences an overall deterioration of health requiring discontinuation of treatment without objective evidence. Every effort should be made to assess objective progression even after treatment discontinuation. Symptomatic deterioration is not an assessment description of an objective response: it is the reason for discontinuation of treatment. The objective response of such patients will be assessed by the target and non-target lesions shown in Tables 1 through 3.

Defined as early progression, early death and non-evaluable conditions are study-specific and should be clearly described in each protocol (depending on treatment interval and treatment cycle).

In some cases, it is difficult to distinguish local lesions from normal tissue. When the assessment of complete response is based on such a definition, we recommend a biopsy prior to response assessment of complete response in local lesions. FDG-PET is used as a similar assessment criterion to biopsy for confirmation of complete response when abnormalities in local lesion imaging are considered representative of lesion fibrosis or scarring in some patients. In such cases, the use of FDG-PET should be prospectively described in the protocol, supported by reports from the specialized medical literature for this condition. However, it must be appreciated that FDG-PET and biopsy itself will lead to false-positive results at the time of CR assessment due to limitations (including resolution and sensitivity of both).

Table 1 Time Point Response: patients with Target Lesions (Including or Excluding Non-Target Lesions)

Target Lesions	Non-Target Lesions	New Lesions	Overall response
CR	CR	Non	CR
CR	Non-CR/Non-PD	Non	PR
CR	Not evaluable	Non	PR
PR	Non-progression or incomplete assessment	Non	PR
SD	Non-progression or incomplete assessment	Non	SD
Not fully evaluated	Non-progression	Non	NE
PD	Any situation	Yes or No	PD
Any situation	PD	Yes or No	PD
Any situation	Any situation	Yes	PD
CR = complete response	PR = partial response	SD = stable disease	PD = progressive disease<br>NE = Not evaluable

Table 2 Time Point Response – Patients with Non-Target Lesions Only

Non-Target Lesions	New Lesions	Overall response
CR	Non	CR
Non-CR or non-PD	Non	Non-CR or Non-PD
Not fully evaluated	Non	Not evaluable
Unequivocal PD	Yes or No	PD
Any situation	Yes	PD

Note: for non-target lesions, "non-CR/non-PD" refers to efficacy superior to SD. Since SD is increasingly used as an endpoint to evaluate response, non-CR/non-PD response is developed for situations where no lesions are specified to be measurable.

Treatment for equivocal progression findings (eg, very small indeterminate new lesions; cystic or necrotic lesions of preexisting lesions) may continue until the next evaluation. If disease progression is confirmed at the next assessment, the date of progression should be the date of previous suspected progression.

Table 3 Best Overall Response Requiring Confirmation of CR and PR Response

Overall response at first timepoint	Overall response at subsequent timepoints	Best overall response

CR	CR	CR
CR	PR	SD, PD or PRa
CR	SD	SD if SD persists for sufficient time, otherwise it should be PD
CR	PD	SD if SD persists for sufficient time, otherwise it should be PD
CR	NE	SD if SD persists for sufficient time, otherwise NE
PR	CR	PR
PR	PR	PR
PR	SD	SD
PR	PD	SD if SD persists for sufficient time, otherwise it should be PD
PR	NE	SD if SD persists for sufficient time, otherwise NE
NE	NE	NE

Note: CR is complete response, PR is partial response, SD is stable disease, PD is progressive disease, and NE is not evaluable. Superscript "a" : if a CR is truly present at the first time point and any disease at a subsequent time point, the patient ' s response will be assessed as PD at a later time point (since disease will reappear after CR) even if the patient ' s response meets the criteria for PR relative to baseline. Best response depends on SD within the shortest treatment interval. However, sometimes the first assessment is CR, but subsequent time point scans suggest that small lesions appear to still be present, so in fact the patient response should be PR rather than CR at the first time point. In this case, the first CR determination should be modified to PR, and the best response is PR.

4.5 Frequency of tumor re-evaluation

The frequency of tumor re-evaluation during treatment depends on the treatment regimen and should be consistent with the type and schedule of treatment. However, in phase II trials where the benefit of treatment is unclear, follow-up every 6 to 8 weeks (time is designed at the end of a cycle) is justified, and the length of the interval can be adjusted in special regimens or circumstances. Protocols should specify which tissue sites require baseline assessments (usually those most likely to be closely related to metastatic lesions of the tumor type under study) and the frequency of evaluation repeats. Normally, target and non-target lesions should be evaluated at each assessment, and in some optional cases, some non-target lesions may be evaluated less frequently, e.g., repeat bone scans are required when response assessment of target disease is confirmed as CR or progression of bone lesions is suspected.

After completion of treatment, tumor re-evaluation depends on whether the response rate or time to an event (progression/death) is used as the endpoint of the clinical trial. If the time to an event (e.g., TTP/DFS/PFS), routine repeat evaluations as specified in the protocol are required. Particularly in randomized comparative trials, predefined evaluations should be listed

in the schedule (e.g., 6 to 8 weeks on treatment, or 3 to 4 months after treatment) and should not be affected by other factors, such as treatment delay, dosing interval, and any other events that may lead to imbalance in the treatment arm in the timing of disease evaluation.

4.6 Response Assessment/Confirmation of Response Period

4.6.1 Qualification

For nonrandomized clinical studies where efficacy is the primary endpoint, the efficacy of PR and CR must be confirmed to ensure that the response is not the result of an evaluation error. This also allows for a reasonable interpretation of the results where historical data are available, but efficacy should also be confirmed in historical data from these trials. However, in all other cases, such as randomized trials (Phase II or III) or studies with stable disease or disease progression as the primary endpoint, confirmation of efficacy is no longer required as this is not valuable for the interpretation of trial results. Cancellation of the requirement for confirmation of efficacy, however, makes central review of the role of prevention of bias even more important, particularly in unblinded experimental studies.

In the case of SD, at least one measurement met the SD criteria specified in the protocol at the shortest interval after the start of the trial (generally not less than 6 to 8 weeks).

4.6.2 Overall Response Period

The overall response period is the time from the first measurement that criteria for CR or PR (whichever is first measured) are met to the first true documentation of disease recurrence or progression (taking the smallest measurement recorded in the trial as reference for disease progression). The time to overall complete response is the time from measurement of the first time criteria for CR are met to the first true documentation of disease recurrence or progression.

4.6.3 Stable disease

Time from start of treatment to progression (in randomized trials, from time of randomization) with the smallest sum in the trial as reference (if the baseline sum is the smallest, it will be used as reference for PD calculation). The clinical relevance of stable disease varies by study and disease. If the proportion of patients maintaining the shortest period of stability is used as the endpoint in a particular trial, the protocol should specify the shortest time interval between the two measurements in the definition of SD.

Note: response, stability, and PFS are influenced by the frequency of follow-up after baseline evaluation. Defining the standard follow-up frequency is not within the scope of this guideline. The frequency of follow-up should take into account a number of factors, such as disease type and stage, treatment cycle, and standard practice. However, the limitations of the accuracy of these measured endpoints should be considered if inter-assay comparisons are required.

4.7 PFS/TTP

4.7.1 Phase 2 clinical trial

This guideline focuses primarily on the use of objective response as an endpoint in phase 2 clinical trials. In some cases, response rates may not be the optimal option to evaluate the

potential anticancer activity of new drugs/regimens. In these cases, PFS/PPF at the cut-off time point can be considered a suitable surrogate to provide the raw signal of the biological activity of the new drug. However, it is clear that in an uncontrolled trial, these evaluations will be questioned because seemingly valuable observations may be related to biological factors such as patient screening rather than the effects of pharmacological interventions. Therefore, phase II clinical trials with these as study endpoints are best designed with randomized controlled trials. However, the clinical presentation of some tumors is consistent (usually always poor) and nonrandomized trials are justified. However, in these cases, evidence of efficacy should be carefully documented when assessing the expected PFS or PPF due to the lack of an active control.

Subsequent contents are Phase 3 evaluation endpoints, independent evaluations, results report, etc., as detailed in the English version.

Attachment 8 Drugs that May Prolong QTc

The following table lists drugs known to prolong QTc for information. This list does not contain all drugs. Please refer to individual drug package inserts for specific information on whether known compounds prolong QTc.

Drug Type	Generic Drug Name
Class IA antiarrhythmic agents	Quinidine procainamide disopyramide
Class IC antiarrhythmic agents	Flecainide propafenone moricizine
Class III antiarrhythmic agents	Amiodarone sotalol benzylammonium bromide ibutilide dofetilide
Antipsychotics	Thioridazine Mesodazine Chlorpromazine Prochlorperazine Trifluoperazine<br>Fluphenazine Perphenazine Pimozide Risperidone Ziprasidone<br>Haloperidol
Tricyclic/tetracyclic antidepressants	Amitriptyline, desipramine, doxepin, dulpine, imipramine Maprotiline
Selective serotonin and norepinephrine reuptake inhibitors (SSNRIs) antidepressants	Venlafaxine
Macrolide antibiotics	Azithromycin Erythromycin Clarithromycin Dirithromycin Roxithromycin Tolamycin
Fluoroquinolones	Moxifloxacin Gatifloxacin
Azole antifungals	Ketoconazole Fluconazole Itraconazole Posaconazole Voriconazole
Antimalarials	Amodiaquine Atovaquone Chloroquine<br>Halofonite, mefloquine, chloroguanidine, primaquine<br>Pyrimethamine Quinine Sulfadoxine
Antigenic insecticide	Pentamidine
Antiemetics	Droperidol Dorasetron Granisetron Ondansetron
Antiestrogens	Tamoxifen
Immunosuppressants	Tacrolimus

Attachment 9 COVID-19

Study guidelines during the Covid-19 pandemic:

On 11 March 2020, a severe acute respiratory syndrome coronavirus type 2 (SARS-CoV-2) was declared a global epidemic. Therefore, it is recognized that the 2019 coronavirus (COVID-19) outbreak may have an impact on study behavior due to self-isolation/isolation of patients and study site staff, travel restrictions, reduced access to personal caregivers/family members, and limited access to public areas, including hospitals and clinics.

In accordance with recent health authority guidance, the Sponsor is providing options for study-related patient management in case the study is interrupted or scaled down. This guidance does not supersede local or governmental requirements, nor does it supersede the clinical judgment of investigators to protect the health and well-being of patients and field staff. At any time, study intervention will be discontinued if a patient is considered to be at safety risk and subsequent procedures will be conducted in a manner that minimizes infectious contact.

Scheduled follow-up that cannot be performed in person at the study site should be postponed until it can be resumed to central follow-up. At each visit (or remote follow-up as needed), patients will be followed up to collect safety data. Efficacy endpoints should be assessed if needed and feasible. Patients will also be asked about their general health.

Patients should make every effort to comply with protocol-specific assessments, including follow-up. Protocol-required assessments may be modified due to the COVID-19 pandemic after consultation with patients, investigators, and the sponsor. Missed assessments/visits should be documented in the clinical trial management system as protocol deviations related to COVIDs. Discontinuation/interruption of study intervention and/or withdrawal from the study should be recorded in the case report form (CRF) with the prefix "COVID9-related".

The sponsor will continue to monitor the conduct and progress of the clinical study, and any changes will be notified to the study site and health authorities in accordance with local guidelines. If a patient tested positive for COVID-19, the investigator should contact the sponsor ' s responsible medical monitoring personnel to discuss the study intervention and follow-up plan. Modifications to the study progress due to the COVID-19 epidemic should be summarized in the clinical study report.
